# Sugar Amino Acid Linkers for the Design of [^18^F]SiFA‐PSMA Radioligands

**DOI:** 10.1002/cmdc.70436

**Published:** 2026-08-12

**Authors:** Narendar R. Gade, Melinda Wuest, Justin J. Bailey, Atul Bhardwaj, Ralf Schirrmacher, Frank Wuest

**Affiliations:** ^1^ Department of Oncology Cross Cancer Institute University of Alberta Edmonton Alberta Canada; ^2^ Department of Chemistry University of Alberta Edmonton Alberta Canada; ^3^ Cancer Research Institute of Northern Alberta Edmonton Alberta Canada

**Keywords:** fluorine‐18, PET imaging, prostate cancer, PSMA, silicon‐fluoride‐acceptor, sugar amino acids

## Abstract

The pronounced lipophilicity of *tert*‐butyl‐substituted silicon‐fluoride‐acceptor (SiFA) scaffolds represents a major limitation in radiotracer development, often resulting in elevated nonspecific uptake and reduced tumor‐to‐background ratios. To address this challenge, we designed and synthesized a series of prostate‐specific membrane antigen (PSMA)‐targeted radioligands incorporating furanose‐ and pyranose‐based sugar amino acid (SAA) linkers to evaluate physicochemical properties while maintaining efficient ^18^F‐radiolabeling capability. Six compounds (**19** **–24**) were prepared via a convergent three‐step synthesis in 35%–75% overall yield. Furanose‐SAA derivatives (**19–22**) retained moderate PSMA binding affinity (IC_50_ = 5–19 µM), while pyranose‐SAA analogues **23** and **24** were inactive (IC_50_ > 100 µM). All compounds exhibited similar lipophilicity (log*P* = 3.10–3.42), indicating that the differences in biological activity are not primarily attributable to lipophilicity. Compounds **19** and **21** were radiolabeled via isotopic exchange in 25%–40% radiochemical yield with molar activity of 86 ± 3 GBq/µmol. PET imaging in LNCaP xenograft mice demonstrated that **[^18^F]21**, bearing three furanose‐SAA units, achieved SUV_60min_ = 0.64 ± 0.06 with  ~30% blocking by DCFPyL, confirming partial PSMA‐mediated uptake. This proof‐of‐concept study establishes SAA linkers as a viable strategy for pharmacokinetic optimization of SiFA‐based radiotracers, warranting further structural refinement to enhance target affinity.

## Graphical abstract

Sugar amino acid (SAA) linkers were incorporated into silicon‐fluoride‐acceptor (SiFA)‐prostate‐specific membrane antigen (PSMA) radioligands to modulate pharmacokinetic properties while preserving efficient ^18^F isotopic exchange radiolabeling. Furanose‐based SAAs outperformed pyranose analogues, enabling specific tumor visualization by PET despite reduced PSMA affinity. These findings establish SAA linkers as promising structural elements for future optimization of SiFA‐based radiotracers.

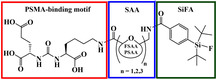

## 1 Introduction

Positron emission tomography (PET) is a cornerstone of molecular imaging, enabling noninvasive, quantitative, and highly sensitive visualization of physiological and pathological processes in vivo. In oncology, PET has revolutionized cancer diagnosis, staging, and treatment monitoring, particularly with fluorine‐18 (^18^F)‐labeled radioligands. The favorable nuclear properties of ^18^F, including a 109.8 min half‐life, low positron energy (0.64 MeV), and high positron yield, facilitate high‐resolution imaging and support centralized radiopharmaceutical production with regional distribution, thereby enabling widespread clinical adoption [1, 2]. Consequently, the development of ^18^F‐labeled radioligands remains a central focus in nuclear medicine research. A significant advancement in ^18^F radiochemistry is the silicon‐fluoride‐acceptor ([^18^F]SiFA) isotopic exchange methodology, which enables efficient radiolabeling of biomolecules through isotopic exchange at a preformed Si─^19^F bond under mild reaction conditions. Recent developments have demonstrated that this approach is compatible with reaction mixtures containing significant amounts of water, eliminating the need for rigorously anhydrous labeling conditions [3, 7].

In contrast to conventional nucleophilic substitution approaches, which often require harsh reaction conditions and multistep synthetic sequences, [^18^F]SiFA labeling proceeds rapidly at ambient temperature and is compatible with sensitive biomolecules, including peptides and peptidomimetics. The incorporation of sterically demanding *tert*‐butyl substituents enhances hydrolytic stability by reducing the Lewis acidity of silicon and providing steric protection of the Si─F bond. These methodological advances have facilitated the clinical translation of [^18^F]SiFA‐based radiotracers, such as [^18^F]SiTATE, [^18^F]Ga‐rhPSMA‐7.1, and [^18^F]Ga‐rhPSMA‐7.3 (POSLUMA) [3, 4]. Moreover, radiohybrid strategies that combine SiFA moieties with metal chelators allow a single molecular scaffold to be labeled with either fluorine‐18 or therapeutic radiometals, thereby enabling matched diagnostic and therapeutic (theranostic) applications. Despite these advantages, the pronounced lipophilicity of *tert*‐butyl‐substituted SiFA scaffolds represents a major limitation. Elevated lipophilicity can result in increased nonspecific uptake in lipid‐rich tissues, prolonged circulation times, and reduced tumor‐to‐background ratios, thereby compromising image quality and diagnostic accuracy [8].

Various strategies have been explored to mitigate these effects, including PEGylation, incorporation of hydrophilic amino acids, positively charged quaternary ammonium salts, and glycosylation [9, 11], and, more recently, structural modifications of the SiFA core itself, such as cycloSiFA and HetSiFA, which reduce the intrinsic lipophilicity of the SiFA motif while maintaining favorable radiolabeling characteristics.

**FIGURE 1:**
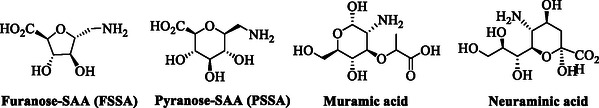
Structures of representative SAAs, including muramic acid and neuraminic acid.

Sugar amino acids (SAAs) represent a promising yet underexplored class of compounds for pharmacokinetic modulation in the context of lipophilic SiFA scaffolds. SAAs are carbohydrate‐derived amino acid analogues containing amino and carboxyl functionalities within a cyclic sugar framework. Although they lack the canonical α‐amino acid backbone, the term “sugar amino acids” is well established for this class of compounds in carbohydrate and medicinal chemistry [12, 13]. This unique architecture confers several advantageous properties, including intrinsic hydrophilicity derived from multiple hydroxyl groups, conformational rigidity, high stereochemical complexity, and versatile functionalization through orthogonal reactive sites [14]. Naturally occurring SAAs, such as muramic acid and neuraminic acid, fulfill essential biological roles, whereas synthetic SAAs have been extensively employed in medicinal chemistry as scaffolds for peptidomimetics, glycopeptides, and drug conjugates (Figure 1) [15, 16].

The rigid conformational profiles of SAAs can enhance receptor binding affinity and proteolytic stability. When incorporated into peptide sequences, SAAs can induce well‐defined secondary structures such as β‐turns and α‐helices. Beyond ligand design, SAAs have been applied in drug delivery systems, nanoparticle platforms, polymer therapeutics, and vaccine development, owing to their hydrophilicity, biocompatibility, and capacity to mimic glycan epitopes. Additionally, certain SAAs exhibit antimicrobial and antiviral activity, further underscoring their structural versatility [17, 19].

Despite their broad utility in medicinal chemistry, SAAs have remained largely unexplored in the field of radiopharmaceutical development. Their inherent structural properties, particularly their ability to modulate hydrophilicity, impose conformational constraints, and enable multivalent functionalization, are well suited to address current challenges in radiotracer optimization.

We propose that the incorporation of furanose‐ and pyranose‐based SAAs (FSAA or PSAA) into [^18^F]SiFA radioligands offers a rational and modular strategy to balance lipophilicity, improve pharmacokinetic profiles, and enhance tumor targeting (Figure 1). The intrinsic hydrophilicity of SAAs may counteract the lipophilic character of the SiFA core, while their conformational rigidity has the potential to improve binding affinity and in vivo stability. To evaluate this strategy, we investigated SAA‐modified [^18^F]SiFA radioligands using prostate‐specific membrane antigen (PSMA) as a well‐established cancer target in this proof‐of‐concept study.

## 2 Results and Discussion

**FIGURE 2:**
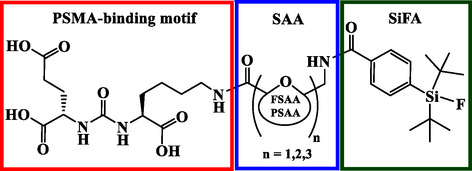
General structure of SiFA‐(F/P)SAA‐PSMA compounds.

To develop novel PSMA‐targeted radioligands with improved pharmacokinetic properties, we designed and synthesized a series of SiFA‐(F/P)SAA‐PSMA compounds that integrate three functional components: 1) a PSMA‐binding pharmacophore for tumor targeting (red), 2) a FSAA or PSAA spacer to modulate hydrophilicity and biodistribution (blue), and 3) a SiFA moiety to enable rapid ^18^F‐radiolabeling under mild conditions (green). The modular synthetic strategy was conceived to allow systematic exploration of different sugar linkers while maintaining the essential targeting and radiolabeling functionalities (Figure 2).

**FIGURE 3:**
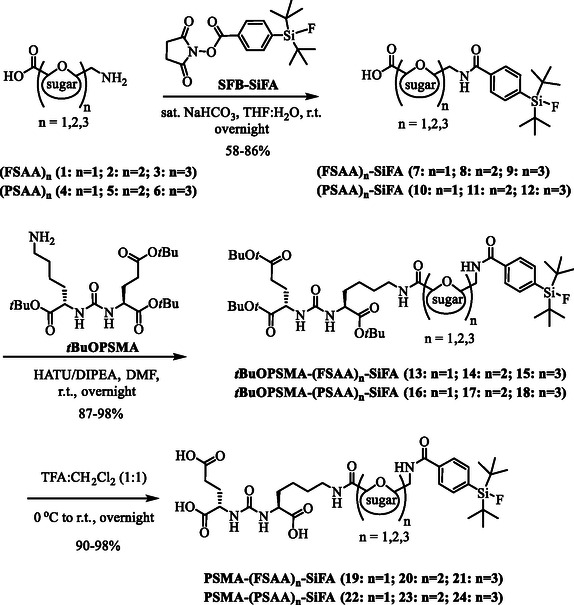
Synthesis of PSMA‐(F/P)SAA‐SiFA compounds.

The synthesis was accomplished through a convergent three‐step approach that provided high and reproducible yields across multiple analogues (Figure 3).

In the first step, various SAAs **1–** **6** were successfully coupled with the SiFA building block SFB‐SiFA [20] under mild aqueous conditions, affording the SiFA‐(F/P)SAA intermediates **7** **–12** in good to excellent yields (58%–86%). This initial coupling proceeded cleanly and established the foundation for subsequent elaboration toward the final PSMA‐targeted conjugates.

The key coupling step involved amide bond formation between the PSMA‐binding Lys‐urea‐Glu (K‐urea‐E) pharmacophore and the SiFA‐(F/P)SAA intermediates **7–12**. This reaction proceeded smoothly, affording the protected *t* *ert*‐BuOPSMA‐(F/P)SAA‐SiFA conjugates **13** **–18** in excellent yields (87%–98%). The consistently high conversions across all analogues demonstrate that SAA incorporation is synthetically well tolerated and that the coupling protocol is compatible with the diverse functional groups present in these structurally complex molecules. Global deprotection with trifluoroacetic acid efficiently removed the protecting groups to furnish the target PSMA‐(F/P)SAA‐SiFA conjugates **19** **–24**, exposing the free carboxylic acid functionalities required for PSMA recognition. Overall, the three‐step synthesis provided the desired compounds in 35%–75% yield (Figure 3), establishing a practical and modular route to structurally diverse SiFA‐PSMA derivatives suitable for isotopic exchange labeling with no‐carrier‐added [^18^F]fluoride. The robustness of this synthetic strategy should facilitate future structure–activity relationship studies by enabling rapid variation of SAA linker composition and architecture without substantial modification of the overall synthetic route.

All compounds were tested for their PSMA inhibitory potency in an in vitro competitive binding assay using PSMA‐expressing LNCaP cells with [^18^F]PSMA‐1007 as the radioligand according to a modified procedure published by our research group [19]. PSMA‐1007, a high‐affinity PSMA inhibitor, was used as a reference compound. In addition, the lipophilicity (logP) values provide further insight into structure–activity relationships of these compounds (Table 1).

**TABLE 1:** In vitro inhibition potencies (IC_50_ values, mean ± standard deviation) and lipophilicity (logP) values (mean ± standard deviation) determined by HPLC [21, 22] of control (PSMA‐1007) and PSMA‐(F/P)SAA‐SiFA compounds.

Compound	**IC**_**50**_ **[μM] (*n* = 3)**	logP (*n* = 3)
PSMA‐1007	0.004 ± 0.001	0.85 ± 0.11
**19**	5 ± 1.1	3.42 ± 0.05
**20**	10 ± 1.8	3.30 ± 0.04
**21**	19 ± 3.3	3.26 ± 0.05
**22**	11 ± 1.9	3.31 ± 0.03
**23**	>100	3.25 ± 0.03
**24**	>100	3.10 ± 0.04

The in vitro binding assay results presented in Table 1 demonstrate marked differences in the inhibitory potencies of the novel PSMA‐(F/P)SAA‐SiFA compounds compared with the reference ligand PSMA‐1007 (IC_50_ = 0.004 ± 0.001 μM). Among the synthesized derivatives, the furanose‐SAA compounds **19** **–22** retained measurable PSMA binding affinity, with IC_50_ values ranging from 5 to 19 μM, whereas the pyranose‐containing analogues **23** and **24** showed no measurable inhibition within the tested concentration range (IC_50_ > 100 μM). Since all compounds exhibited nearly identical lipophilicity (log*P* = 3.10–3.42), the observed differences in receptor binding are unlikely to arise from physicochemical properties alone but instead reflect conformational and geometric effects introduced by the respective sugar linkers. Compared with PSMA‐1007, which possesses low‐nanomolar affinity, incorporation of the SAA linker substantially reduced PSMA binding, suggesting that the spatial presentation of the Lys‐urea‐Glu pharmacophore relative to the entrance funnel of the PSMA binding site is altered, thereby disrupting key receptor interactions required for high‐affinity binding. Nevertheless, the pronounced difference between the furanose‐ and pyranose‐based derivatives indicates that linker architecture plays a critical role in PSMA recognition. The superior performance of the furanose‐SAA derivatives may reflect the greater conformational flexibility of the compact five‐membered furanose ring, which could provide a more favorable orientation of the Lys‐urea‐Glu pharmacophore within the PSMA binding pocket. In contrast, the more rigid six‐membered pyranose scaffold may constrain pharmacophore presentation and compromise productive receptor interactions. Although additional structural and computational studies are required to validate this hypothesis, these findings suggest that subtle differences in linker geometry can have a pronounced impact on PSMA binding affinity.

The experimentally determined logP values in Table 1 indicate that all PSMA‐(F/P)SAA‐SiFA derivatives are highly lipophilic, with logP values ranging from 3.10 to 3.42, whereas the reference compound PSMA‐1007 is considerably more hydrophilic (log*P* = 0.85). Despite exhibiting comparable lipophilicity, the furanose‐SAA derivatives **19–22** retained moderate PSMA binding affinity (IC_50_ = 5–19 μM), whereas the pyranose‐SAA analogues **23** and **24** showed no measurable binding (IC_50_ > 100 μM). These findings indicate that the differences in biological activity cannot be attributed primarily to lipophilicity but are more likely a consequence of the structural influence of the furanose and pyranose SAA linkers, particularly their linker geometry, on the presentation of the PSMA pharmacophore.

The uniformly high lipophilicity of the SiFA‐containing derivatives also highlights that further optimization will be required to reduce nonspecific uptake while preserving or improving PSMA affinity.

**FIGURE 4:**
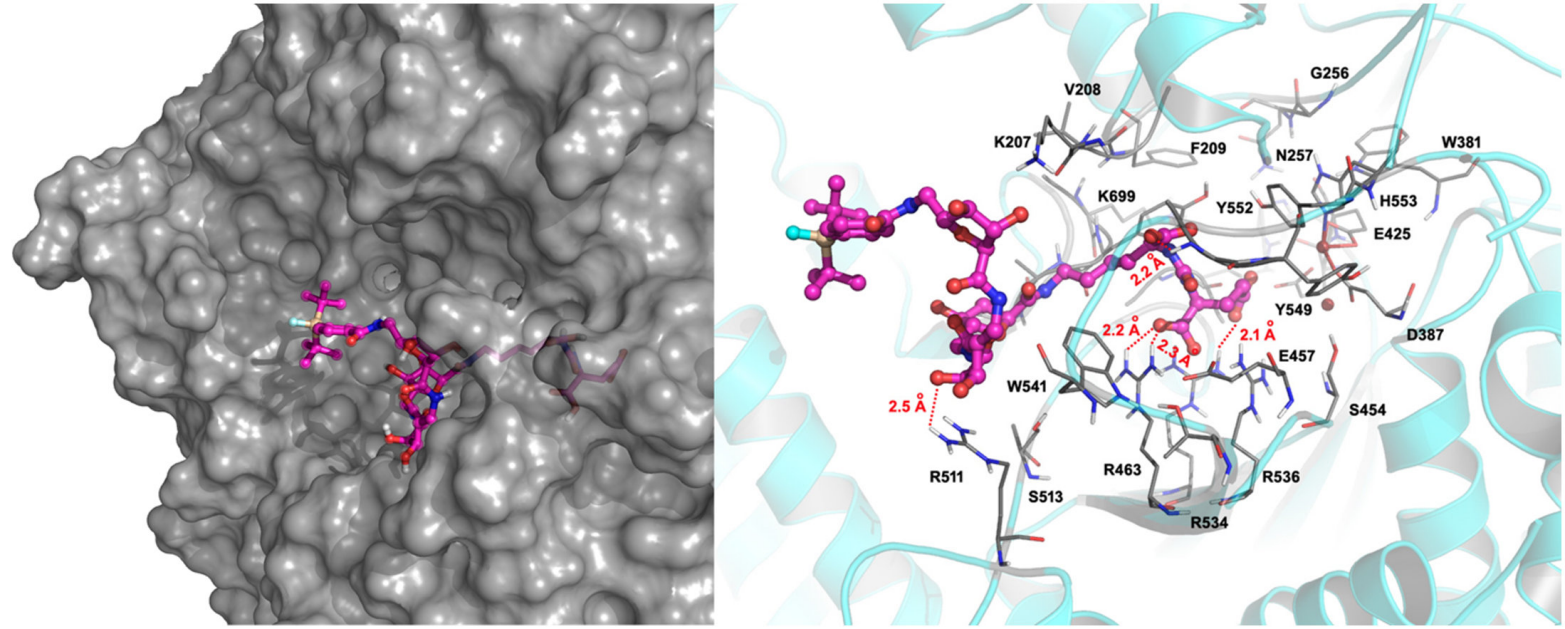
Molecular docking results for **21** docked in PSMA (PDB: 5O5T).

Molecular docking studies were performed using compound **21** to elucidate the molecular basis of ligand recognition and to understand how incorporation of the SAA linker influences binding relative to PSMA‐1007. Although compound **21** exhibited the weakest binding affinity among the furanose derivatives (IC_50_ = 19 μM), it was selected for docking because its longer SAA linker provided an opportunity to examine how increasing linker length influences interactions within the PSMA binding site. This structural analysis helps explain the reduced binding affinity and provides insights into the rational optimization of future PSMA‐targeted radioligands incorporating SAA linkers. As shown in Figure 4, the ligand (magenta, ball‐and‐stick) adopts a defined pose in a deep, predominantly polar pocket, stabilized by electrostatic and hydrogen bonding interactions with key residues. The binding pocket comprises a diverse set of residues forming a multifaceted recognition site, with a defined entrance, polar and aromatic sidewalls, and a deep arginine‐rich region supported by additional polar residues. The ligand is deeply embedded and oriented to maximize complementarity with this architecture.

Binding is primarily driven by an extensive hydrogen bonding network, including five key interactions (2.1–2.5 Å), indicating strong to moderate electrostatic stabilization. Notably, a short 2.1 Å hydrogen bond represents a particularly favorable interaction. The arginine‐rich patch likely forms salt bridges and hydrogen bonds with polar ligand moieties, while acidic residues (E425, E457, and D387) contribute to a complementary electrostatic environment. Aromatic residues (W381, Y549, Y552, and H553) enable π‐stacking and cation–π interactions, and hydrophobic residues (V208, F209, W381, and W541) form a subpocket for nonpolar ligand regions. Overall, the ligand’s binding mode is stabilized by a combination of hydrogen bonding, electrostatic, and hydrophobic interactions.

Furanose‐containing compounds **19** and **21** (30–50 nmol) were radiolabeled with ^18^F via isotopic exchange using our optimized “four‐drop method” [7]. Cyclotron‐produced [^18^F]fluoride (1.0–1.3 GBq) was trapped on a predried QMA cartridge and eluted with Kryptofix(2.2.2)/K_2_CO_3_ solution, collecting only the first four drops (~100 μL), which contain >80% of the radioactivity. This minimized base content and eliminated the need for neutralization.

**FIGURE 5:**
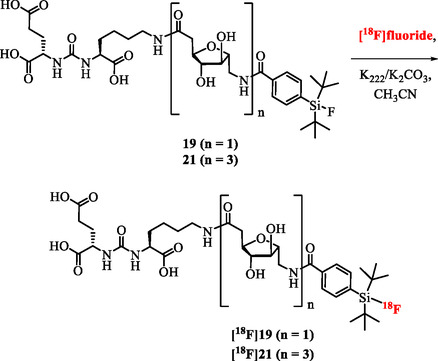
Radiosynthesis of FSAA compounds **[** ^**18**^ **F]19** and **[** ^**18**^ **F]21**.

The eluate was dried (90–100 °C, 5 min), cooled, resuspended in acetonitrile (50 μL), and added to a reaction vial containing SiFA precursor (**19** and **21**) dissolved in DMSO (15–20 μL). The isotopic exchange reaction was performed at 95 °C for 40 min, followed by purification using HPLC. The collected radiotracer fraction was concentrated under high vacuum, then formulated in isotonic saline, and sterile filtered. The method afforded moderate radiochemical yields of 25%–40% (decay‐corrected), molar activities of 86 ± 3 GBq/μmol (*n* = 8), and a radiochemical purity ≥98%. The radiosynthesis of compounds **[** ^**18**^ **F]19** and **[** ^**18**^ **F]21** is shown in Figure 5.

**FIGURE 6:**
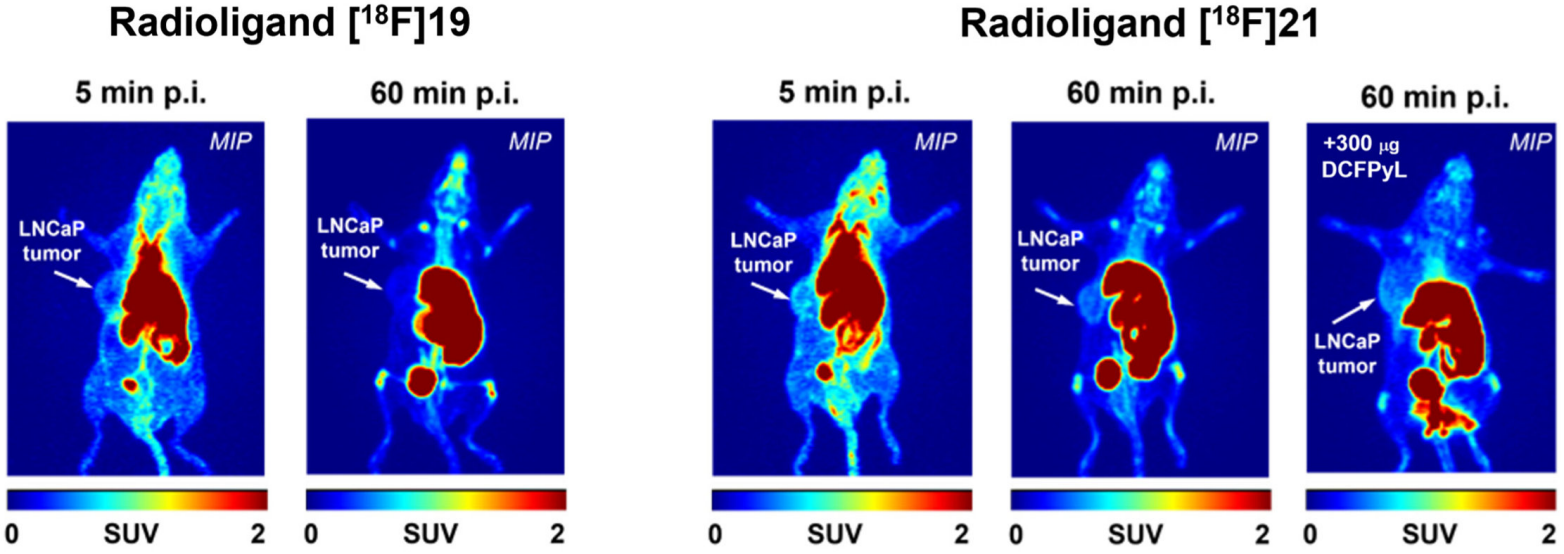
PET images of radioligands **[** ^**18**^ **F]19** and **[** ^**18**^ **F]21**.

**FIGURE 7:**
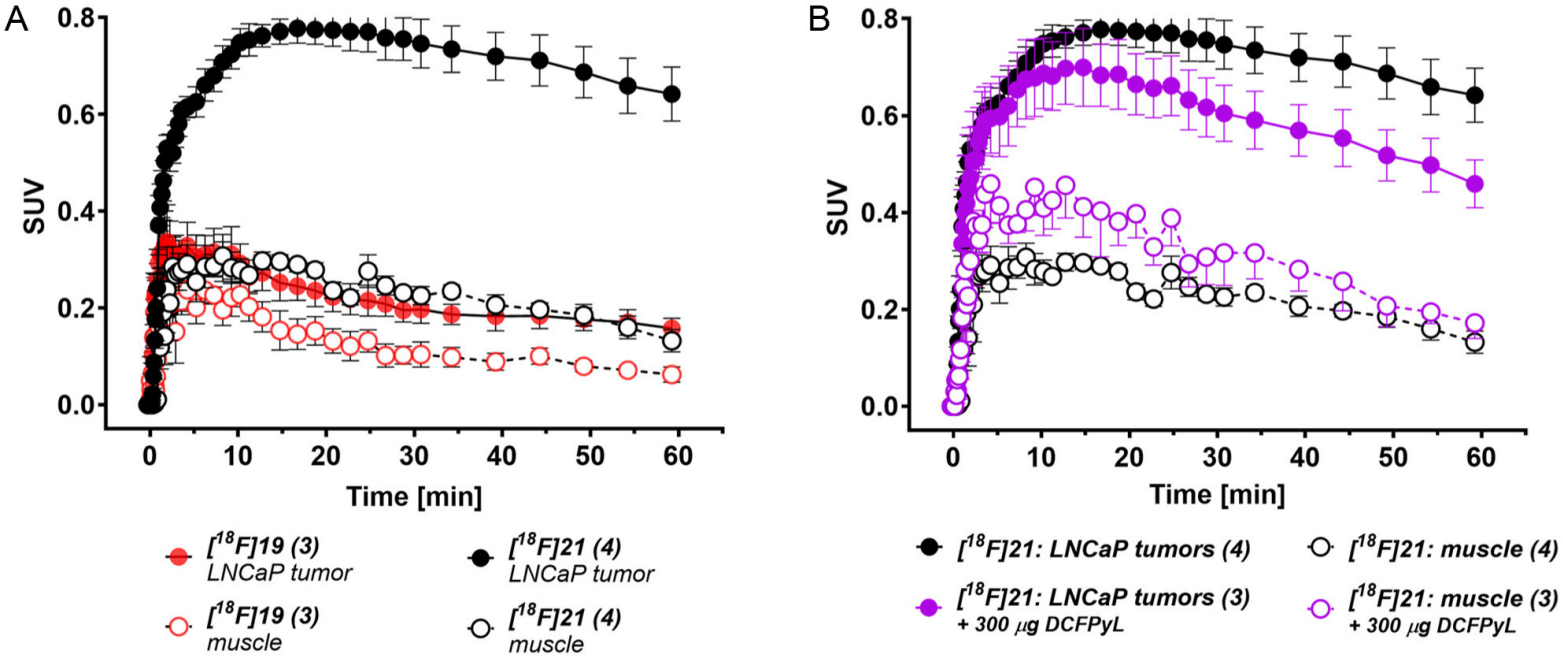
(A) Time‐activity curves (TACs) of radioligands **[** ^**18**^ **F]19** and **[** ^**18**^ **F]21** in LNCaP xenografts and muscle. (B) TACs of radioligand **[** ^**18**^ **F]21** under control and blocking conditions (*n* = 3).

Both radioligands, **[** ^**18**^ **F]19** and **[** ^**18**^ **F]21**, were evaluated in PET imaging studies using LNCaP tumor‐bearing mice to assess tumor targeting (Figure 6) and pharmacokinetics (Figure 7).

Radioligand **[** ^**18**^ **F]21**, containing three furanose‐SAA units, showed substantially higher tumor uptake and retention over 60 min (SUV_60min_ = 0.64 ± 0.06) compared to **[** ^**18**^ **F]19**, which contains a single SAA unit (SUV_60min_ = 0.16 ± 0.04). Accordingly, only **[** ^**18**^ **F]21** was selected for blocking studies. To confirm PSMA‐mediated uptake, blocking studies were performed with 300 µg of the PSMA inhibitor DCFPyL.

Tumor uptake of **[** ^**18**^ **F]21** decreased to SUV_60min_ = 0.46 ± 0.05, corresponding to an ≈30% reduction. This partial blocking confirms that tumor accumulation is partly PSMA‐mediated, consistent with in vitro binding (IC_50_ = 19 µM), while the incomplete inhibition likely reflects reduced PSMA affinity and a residual nonspecific component due to the lipophilicity of the SiFA core. For context, **[** ^**18**^ **F]21** was compared with previously reported SiFA‐based PSMA tracers. The second‐generation [^18^F]SiFA‐Asp_2_‐PEG_3_‐PSMA achieved SUV_60min_ values of 0.73 and 1.18 ± 0.12 at low and high molar activities, respectively, with a similar ~ 32% blocking effect [7], while the third‐generation ^18^F‐PSiMA reached SUV_60min_ = 1.90 ± 0.29 at high molar activity [6]. At matched molar activity (86 ± 3 GBq/µmol), **[** ^**18**^ **F]21** shows tumor uptake comparable to the performance of [^18^F]SiFA‐Asp_2_‐PEG_3_‐PSMA, supporting SAA modification as a viable, though not yet optimized, strategy for pharmacokinetic modulation.

The main limitation remains significant reduction in PSMA binding affinity relative to second‐generation tracers, which restricts PSMA‐specific uptake.

## 3 Conclusion

The pronounced lipophilicity of *tert*‐butyl‐substituted SiFA scaffolds remains a significant challenge for the development of PSMA‐targeted radioligands. In this proof‐of‐concept study, we demonstrated that SAA linkers can be readily incorporated into SiFA‐PSMA conjugates through a robust and modular synthetic strategy while retaining efficient ^18^F‐radiolabeling by isotopic exchange. Although SAA incorporation did not substantially reduce the overall lipophilicity of the resulting radioligands, the biological evaluation revealed clear differences between linker architectures, with furanose‐based SAAs outperforming their pyranose counterparts. These findings indicate that linker geometry, rather than physicochemical properties alone, plays a critical role in determining PSMA binding affinity and tumor targeting. Collectively, this study establishes SAA linkers as promising structural elements for the design of SiFA‐based radioligands and provides a foundation for future optimization. Further work will focus on repositioning and redesigning the SAA linker to preserve optimal presentation of the Lys‐urea‐Glu pharmacophore, thereby improving PSMA affinity while maintaining the favorable radiolabeling characteristics of the SiFA platform.

## 4 Experimental Section

All reagents were obtained from commercial suppliers and used without further purification. SAAs **1** **–6** were purchased from the Canadian Glycomics Network (University of Alberta, 3–572 Edmonton Clinic Health Academy (ECHA), 11 405–87 Avenue NW, Edmonton, Alberta T6G 1C9, Canada). NMR spectra were recorded on a Bruker AM‐600 spectrometer. ^1^H and ^13^C NMR chemical shifts were referenced to residual signals of deuterated solvents as internal standards and are reported in ppm. Coupling constants (J) are given in hertz (Hz). Signal multiplicities are denoted as s (singlet), bs (broad singlet), d (doublet), dd (doublet of doublets), t (triplet), and m (multiplet).

Low‐resolution mass spectra were acquired on an Agilent 6130 mass spectrometer coupled to an Agilent 1260 HPLC system. High‐resolution mass spectra were obtained using either an Agilent Technologies 6220 oaTOF (ESI) or a Kratos MS50G (EI) instrument.

Thin‐layer chromatography was performed on silica gel 60 F254 aluminum plates (MilliporeSigma). Semipreparative HPLC was carried out on a Gilson system (Mandel Scientific, Guelph, ON, Canada) equipped with a 321 pump and a 155 dual‐wavelength detector (210 and 254 nm), using a Phenomenex Jupiter 10 µm Proteo 90 Å (250 × 10 mm, 4.5 µm) C12 column. Analytical HPLC was performed on a Shimadzu system (Mandel Scientific, Guelph, ON, Canada) equipped with a DGU‐20A5 degasser, SIL‐20A HT autosampler, LC‐20AT pump, and SPD‐M20A photodiode array detector.

### 4.1 Synthesis of PSMA‐(F/P)SAA‐SiFA Compounds

#### 4.1.1 General Procedure

##### 4.1.1.1 Step 1

SAA (**1** **–6**, 1.0 eq.) was dissolved in THF/H_2_O (1:1, 200 μL) in a dram vial at room temperature. Saturated aqueous NaHCO_3_ (50 μL) was added, and the mixture was stirred for 5 min. **SFB‐SiFA** [18] (10–15 μmol, 2–3 eq.) was then added, and the reaction was stirred overnight at room temperature. Progress was monitored by LC‐MS. Upon completion, the crude product was purified by preparative HPLC.

##### 4.1.1.2 Step 2

The resulting SiFA‐(F/P)SAA compound (**7** **–12**, 1.0 eq.) and ^***t***^ **BuOPSMA** [21] (1.35 eq.) were dissolved in Dimethylformamide (DMF) (500 μL) at room temperature. Hexafluorophosphate azabenzotriazole tetramethyl uronium (HATU) (1.1 eq.) and *N,N‐*diisopropylethylamine (DIPEA) (3.0 eq.) were added, and the reaction mixture was stirred overnight. Reaction progress was monitored by LC‐MS analysis, and upon completion, the crude product was purified by preparative HPLC.

##### 4.1.1.3 Step 3

The protected compound (**13** **–18**, 5 μmol) was treated with a 1:1 mixture of CH_2_Cl_2_/TFA (1 mL) at 0 °C to room temperature for 2–4 h. The solvent was removed, and the residue was purified by preparative HPLC to afford the final PSMA‐(F/P)SAA‐SiFA compounds **19** **–24** in overall yields of 35%–75% for the three steps.

##### 4.1.1.4 Compound 7

Yield: 70%. 1H NMR (600 MHz, methanol‐d4) *δ* ppm 1.05–1.08 (m, 18 H) 3.61 (dd, J = 14.1, 6.4 Hz, 1 H) 3.71 (dd, J = 13.9, 4.3 Hz, 1 H) 3.96 (dd, J = 4.1, 2.9 Hz, 1 H) 4.22 (dt, J = 6.3, 4.3 Hz, 1 H) 4.35 (t, J = 2.9 Hz, 1 H) 4.42 (d, J = 2.8 Hz, 1 H) 7.72 (d, J = 8.01 Hz, 2 H) 7.87 (d, J = 8.01 Hz, 2 H) ^13^C NMR (150 MHz, methanol‐d4) *δ* ppm 19.6, 19.7, 26.3, 41.8, 78.7, 80.7, 82.7, 84.1, 126.0, 133.8, 133.8, 135.4, 137.7, 137.8, 168.9, 173.5.

##### 4.1.1.5 Compound 8

Yield: 73%. 1H NMR (600 MHz, methanol‐d4) *δ* ppm 1.03–1.10 (m, 18 H) 3.43 (dd, J = 13.9, 6.2 Hz, 1 H) 3.54 (dd, J = 13.9, 4.71 Hz, 1 H) 3.61–3.65 (m, 1 H) 3.68–3.73 (m, 1 H) 3.88 (dd, J = 4.2, 3.1 Hz, 1 H) 3.98–4.01 (m, 1 H) 4.08 (dt, J = 6.2, 4.5 Hz, 1 H) 4.26 (ddd, J = 6.5, 4.6, 3.6 Hz, 1 H) 4.29 (t, J = 3.1 Hz, 1 H) 4.32–4.34 (m, 1 H) 4.36 (dd, J = 6.5, 2.9 Hz, 2 H) 7.72 (d, J = 8.3 Hz, 2 H) 7.87 (d, J = 8.3 Hz, 2 H); ^13^C NMR (150 MHz, methanol‐d4) *δ* ppm 21.0, 21.1, 27.7, 42.1, 43.3, 79.9, 80.0, 81.9, 82.1, 84.0, 85.3, 85.9, 86.0, 127.4, 135.2, 135.2, 136.8, 139.1, 139.2, 170.4, 173.9, 174.8.

##### 4.1.1.6 Compound 9

Yield: 86%. 1H NMR (600 MHz, methanol‐d4) *δ* ppm 1.06–1.08(m, 18 H) 3.31–3.37 (m, 1 H) 3.41–3.52 (m, 2 H) 3.60–3.67 (m, 2 H) 3.69–3.74 (m, 1 H) 3.87 (m, 2 H) 4.03–4.09 (m, 3 H) 4.24–4.29 (m, 2 H) 4.30 (t, J = 3.1 Hz, 1 H) 4.31–4.35 (m, 1 H) 4.36–4.40 (m, 3 H) 7.72 (d, J = 8.1 Hz, 2 H) 7.87 (d, J = 8.1 Hz, 2 H); ^13^C NMR (150 MHz, methanol‐d4) *δ* ppm 21.0, 21.1, 27.7, 42.0, 42.2, 43.3, 79.9, 79.9, 81.9, 82.0, 82.1, 84.0, 85.3, 85.3, 85.5, 86.3, 86.4, 127.4, 135.2, 135.2, 136.8, 139.2, 139.3, 170.3, 173.8, 173.9, 174.8.

##### 4.1.1.7 Compound 10

Yield: 83%. 1H NMR (600 MHz, methanol‐d4) *δ* ppm 1.07 (m, 18 H) 3.20–3.25 (m, 1 H) 3.39–3.49 (m, 3 H) 3.69–3.85 (m, 3 H) 7.73 (d, J = 8.1 Hz, 2 H) 7.86 (d, J = 8.1 Hz, 2 H); ^13^C NMR (150 MHz, methanol‐d4) *δ* ppm 21.0, 21.1, 27.7, 42.0, 72.3, 73.3, 78.7, 79.9, 80.0, 127.5, 135.2, 135.2, 136.7, 139.2, 139.3, 170.8, 173.5.

##### 4.1.1.8 Compound 11

Yield: 59%. 1H NMR (600 MHz, methanol‐d4) *δ* ppm 1.05–1.08 (m, 18 H) 3.15–3.20 (m, 1 H) 3.20–3.26 (m, 1 H) 3.35–3.53 (m, 7 H) 3.64–3.69 (m, 1 H) 3.71–3.82 (m, 4 H) 7.73 (d, J = 8.1 Hz, 2 H) 7.86 (d, J = 8.1 Hz, 2 H); ^13^C NMR (150 MHz, methanol‐d4) *δ* ppm 21.0, 21.1, 27.7, 41.2, 42.0, 72.1, 72.3, 73.3, 73.6, 78.7, 79.7, 79.9, 80.0, 80.0, 127.6, 135.2, 135.3, 136.7, 139.2, 139.3, 170.9, 173.0, 173.2.

##### 4.1.1.9 Compound 12

Yield: 65%. 1H NMR (600 MHz, methanol‐d4) *δ* ppm 1.06–1.08 (m, 18 H) 3.18 (td, J = 9.2, 5.8 Hz, 2 H) 3.23–3.27 (m, 1 H) 3.34–3.52 (m, 11 H) 3.64 (dd, J = 14.1, 2.8 Hz, 1 H) 3.70–3.82 (m, 6 H) 7.73 (d, J = 8.1 Hz, 2 H) 7.86 (d, J = 8.1 Hz, 2 H); ^13^C NMR (150 MHz, methanol‐d4) *δ* ppm 21.0, 21.1, 27.7, 41.1, 41.3, 42.0, 72.0, 72.2, 73.3, 73.5, 73.6, 78.7, 78.7, 79.4, 79.7, 79.8, 79.8, 79.9, 80.0, 127.6, 135.2, 135.3, 136.8, 139.3, 170.9, 172.8, 172.9, 173.4.

##### 4.1.1.10 Compound 13

Yield: 89%. 1H NMR (600 MHz, methanol‐d4) *δ* ppm 1.06–1.08 (m, 18 H) 1.36–1.43 (m, 2 H) 1.43–1.47 (m, 27 H) 1.50–1.56 (m, 2 H) 1.65–1.56 (m, 1 H) 1.69–1.84 (m, 2 H) 1.99–2.07 (m, 1 H) 2.25–2.36 (m, 2 H) 3.13–3.19 (m, 1 H) 3.26 (dt, J = 13.4, 6.9 Hz, 1 H) 3.61–3.72 (m, 2 H) 4.01 (t, J = 2.5 Hz, 1 H) 4.11 (dd, J = 8.2, 5.0 Hz, 1 H) 4.18 (dd, J = 8.7, 5.1 Hz, 1 H) 4.28 (m, 1 H) 4.31–4.34 (m, 2 H) 7.72 (d, J = 8.01 Hz, 2 H) 7.87 (d, J = 8.01 Hz, 2 H); ^13^C NMR (150 MHz, methanol‐d4) *δ* ppm 21.0, 21.1, 23.8, 27.7, 28.3, 28.3, 28.4, 29.0, 30.0, 32.5, 33.1, 39.6, 43.3, 54.2, 54.9, 80.0, 81.8, 82.1, 82.6, 82.9, 86.2, 86.4, 127.4, 135.2, 135.2, 136.8, 139.2, 139.3, 160.0, 170.3, 173.5, 173.6, 173.8, 174.0.

##### 4.1.1.11 Compound 14

Yield: 87%. 1H NMR (600 MHz, methanol‐d4) *δ* ppm 1.05–1.08 (m, 18 H) 1.35–1.42 (m, 3 H) 1.43–1.47 (m, 27 H) 1.48–1.64 (m, 3 H) 1.70–1.83 (m, 2 H) 2.00–2.07 (m, 1 H) 2.25–2.36 (m, 2 H) 3.13–3.23 (m, 2 H) 3.32–3.37 (m, 1 H) 3.59–3.67 (m, 2 H) 3.70–3.74 (m, 1 H) 3.88 (dd, J = 3.8, 3.0 Hz, 1 H) 4.03–4.07 (m, 2 H) 4.11 (dd, J = 8.3, 5.1 Hz, 1 H) 4.19 (dd, J = 8.9, 5.1 Hz, 1 H) 4.22–4.25 (m, 2 H) 4.30–4.34 (m, 1 H) 4.36–4.40 (m, 2 H) 7.72 (d, J = 8.1 Hz, 2 H) 7.87 (d, J = 8.1 Hz, 2 H); ^13^C NMR (150 MHz, methanol‐d4) *δ* ppm 21.0, 21.1, 23.8, 28.3, 28.3, 28.4, 29.0, 30.0, 32.5, 33.0, 39.6, 42.3, 43.3, 54.2, 54.9, 79.9, 80.0, 81.7, 82.1, 82.6, 82.9, 85.4, 85.7, 86.3, 86.4, 127.4, 135.2, 135.2, 136.8, 139.2, 139.3, 160.0, 170.3, 173.5, 173.6, 173.7, 173.8, 174.0.

##### 4.1.1.12 Compound 15

Yield: 92%. 1H NMR (600 MHz, methanol‐d4) *δ* ppm 1.06–1.08 (m, 18 H) 1.36–1.42 (m, 2 H) 1.43–1.48 (m, 27 H) 1.49–1.57 (m, 2 H) 1.58–1.66 (m, 1 H) 1.70–1.85 (m, 2 H) 1.99–2.08 (m, 1 H) 2.25–2.37 (m, 2 H) 3.14–3.25 (m, 2 H) 3.33–3.41 (m, 2 H) 3.53–3.59 (m, 1 H) 3.59–3.67 (m, 2 H) 3.70–3.76 (m, 1 H) 3.89–3.91 (m, 2 H) 4.03–4.05 (m, 1 H) 4.05–4.13 (m, 3 H) 4.17–4.21 (m, 1 H) 4.25–4.27 (m, 2 H) 4.29 (d, J = 10.16 Hz, 2 H) 4.31–4.35 (m, 1 H) 4.36–4.40 (m, 2 H) 7.72 (d, J = 8.1 Hz, 2 H) 7.87 (d, J = 8.1 Hz, 2 H); ^13^C NMR (150 MHz, methanol‐d4) *δ* ppm 21.0, 21.1, 23.8, 27.7, 28.3, 28.3, 28.4, 29.0, 30.0, 32.5, 33.1, 39.7, 42.2, 42.3, 43.4, 54.2, 54.9, 79.9, 80.0, 81.2, 82.1, 82.6, 82.9, 85.5, 85.7, 85.8, 85.9, 86.3, 86.4, 127.4, 135.2, 135.3, 136.8, 139.2, 139.3, 160.0, 170.3, 173.5, 173.6, 173.8, 173.8, 173.9, 174.0.

##### 4.1.1.13 Compound 16

Yield: 90%. 1H NMR (600 MHz, methanol‐d4) *δ* ppm 1.05–1.09 (m, 18 H) 1.37–1.42 (m, 2 H) 1.43–1.47 (m, 27 H) 1.50–1.58 (m, 2 H) 1.58–1.67 (m, 1 H) 1.71–1.84 (m, 2 H) 2.00–2.07 (m, 1 H) 2.25–2.37 (m, 2 H) 3.20–3.27 (m, 3 H) 3.31 (dt, J = 3.3, 1.7 Hz, 3 H) 3.43–3.47 (m, 1 H) 3.68–3.75 (m, 2 H) 3.79–3.84 (m, 1 H) 4.13 (dd, J = 8.3, 5.08 Hz, 1 H) 4.19 (dd, J = 8.9, 5.1 Hz, 1 H) 7.73 (d, J = 8.3 Hz, 2 H) 7.86 (d, J = 8.1 Hz, 2 H); ^13^C NMR (150 MHz, methanol‐d4) *δ* ppm 21.0, 21.1, 23.8, 27.7, 28.3, 28.3, 28.4, 29.0, 29.9, 32.5, 33.1, 39.7, 42.0, 54.2, 54.8, 72.2, 73.6, 78.7, 79.4, 80.0, 81.7, 82.5, 82.8, 127.5, 135.2, 135.3, 136.8, 139.3, 139.4, 159.9, 170.8, 172.4, 173.5, 173.8, 173.9.

##### 4.1.1.14 Compound 17

Yield: 88%. 1H NMR (600 MHz, methanol‐d4) *δ* ppm 1.04–1.10 (m, 18 H) 1.36–1.42 (m, 2 H) 1.42–1.48 (m, 27 H) 1.48–1.65 (m, 4 H) 1.70–1.84 (m, 2 H) 1.98–2.06 (m, 1 H) 2.25–2.37 (m, 2 H) 3.17–3.22 (m, 3 H) 3.23–3.29 (m, 1 H) 3.34–3.38 (m, 1 H) 3.38–3.44 (m, 1 H) 3.45–3.52 (m, 2 H) 3.65–3.81 (m, 5 H) 4.10–4.15 (m, 1 H) 4.17–4.21 (m, 1 H) 7.73 (d, J = 8.1 Hz, 2 H) 7.86 (d, J = 8.1 Hz, 2 H); ^13^C NMR (150 MHz, methanol‐d4) *δ* ppm 21.0, 21.1, 23.9, 27.7, 28.3, 28.4, 29.0, 29.9, 32.5, 33.1, 39.8, 41.1, 42.0, 54.2, 54.9, 72.1, 72.1, 73.6, 73.7, 78.8, 78.8, 79.2, 79.6, 79.8, 80.1, 81.8, 82.6, 82.8, 127.6, 135.3, 135.3, 136.8, 139.2, 139.3, 160.0, 170.9, 172.4, 173.0, 173.6, 173.8, 174.0.

##### 4.1.1.15 Compound 18

Yield: 98%. 1H NMR (600 MHz, methanol‐d4) *δ* ppm 1.05–1.09 (m, 18 H) 1.38–1.43 (m, 2 H) 1.43–1.48 (m, 29 H) 1.51–1.59 (m, 2 H) 1.59–1.67 (m, 1 H) 1.72–1.84 (m, 2 H) 2.00–2.07 (m, 1 H) 2.26–2.37 (m, 2 H) 3.17–3.28 (m, 5 H) 3.34–3.58 (m, 12 H) 3.61–3.80 (m, 7 H) 4.12 (dd, J = 8.19, 5.18 Hz, 1 H) 4.19 (dd, J = 8.85, 5.08 Hz, 1 H) 7.73 (d, J = 8.09 Hz, 2 H) 7.87 (d, J = 8.28 Hz, 2 H); ^13^C NMR (150 MHz, methanol‐d4) *δ* ppm 21.0, 21.1, 24.0, 28.3, 28.4, 29.0, 29.9, 32.5, 33.1, 39.9, 40.4, 41.0, 42.0, 54.2, 54.9, 71.9, 72.1, 72.2, 73.6, 73.6, 78.8, 79.3, 79.4, 79.7, 79.8, 80.1, 81.8, 82.6, 82.9, 127.6, 135.2, 135.3, 136.7, 160.0, 170.9, 172.4, 172.9, 173.0, 173.6, 173.8, 174.1.

##### 4.1.1.16 Compound 19

Yield: 93%. 1H NMR (600 MHz, methanol‐d4) *δ* ppm 1.03–1.10 (m, 18 H; C(CH_3_)_3_) 1.38–1.45 (m, 2 H) 1.54 (m, 2 H) 1.61–1.69 (m, 1 H) 1.77–1.92 (m, 2 H) 2.13 (m, 1 H) 2.34–2.46 (m, 2 H) 3.14 (dt, J = 13.3, 6.7 Hz, 1 H) 3.25–3.29 (m, 1 H) 3.61–3.66 (m, 1 H) 3.67–3.74 (m, 1 H) 4.02 (t, J = 2.6 Hz, 1 H) 4.23 (dd, J = 8.4, 4.8 Hz, 1 H) 4.26–4.31 (m, 2 H) 4.32–4.35 (m, 2 H) 7.72 (d, J = 8.3 Hz, 2 H; C_ar_‐H) 7.86 (d, J = 8.3 Hz, 2 H; C_ar_‐H); ^13^C NMR (150 MHz, methanol‐d4) *δ* ppm 21.0 ( **C** (CH_3_)_3_), 21.1, 23.8, 27.7 (C( **C** H_3_)_3_), 28.9, 30.0, 31.1, 33.0, 39.6, 43.3, 53.5, 54.0, 80.0, 82.0, 86.2, 86.3, 127.4 (C_ar_‐H), 135.2 (C_ar_‐H), 135.2 (C_ar_‐H), 136.8 (C_ar_‐H), 139.3, 160.1 (C═O), 170.4 (C═O), 173.5 (C═O), 176.0 (C═O), 176.4 (C═O), 176.5 (C═O). HRMS (ESI) m/z: (M‐H)‐ cald. for C33H51FN4O12Si 742.3184, found 742.3294.

##### 4.1.1.17 Compound 20

Yield: 91%. 1H NMR (600 MHz, methanol‐d4) *δ* ppm 1.05–1.08 (m, 18 H; C(CH_3_)_3_) 1.36–1.44 (m, 2 H) 1.47–1.58 (m, 2 H) 1.60–1.68 (m, 1 H) 1.76–1.84 (m, 1 H) 1.84–1.92 (m, 1 H) 2.13 (m, 1 H) 2.35–2.46 (m, 2 H) 3.11–3.18 (m, 1 H) 3.23 (dt, J = 13.1, 6.5 Hz, 1 H) 3.32–3.37 (m, 1 H) 3.59–3.67 (m, 2 H) 3.69–3.74 (m, 1 H) 3.88 (dd, J = 3.9, 3.1 Hz, 1 H) 4.03–4.08 (m, 2 H) 4.22–4.26 (m, 3 H) 4.29–4.34 (m, 2 H) 4.36–4.40 (m, 2 H) 7.72 (d, J = 8.3 Hz, 2 H; C_ar_‐H) 7.87 (d, J = 8.3 Hz, 2 H; C_ar_‐H); ^13^C NMR (150 MHz, methanol‐d4) *δ* ppm 21.0 ( **C** (CH_3_)_3_), 21.1, 23.7, 27.7 (C( **C** H_3_)_3_), 28.9, 29.9, 31.1, 33.0, 39.6, 42.3, 43.3, 53.5, 54.0, 80.0, 80.0, 82.0, 85.4, 85.6, 86.3, 86.4, 127.4 (C_ar_‐H), 135.2 (C_ar_‐H), 135.3 (C_ar_‐H), 136.8 (C_ar_‐H), 139.2, 139.3, 160.1 (C═O), 170.3 (C═O), 173.6 (C═O), 173.8 (C═O), 176.0 (C═O), 176.5 (C═O); HRMS (ESI) m/z: (M‐2H)−2 cald. for C39H60FN5O16Si 449.6821, found 449.6820.

##### 4.1.1.18 Compound 21

Yield: 92%. 1H NMR (600 MHz, methanol‐d4) *δ* ppm 1.07 (m, 18 H; C(CH_3_)_3_) 1.37–1.45 (m, 2 H) 1.47–1.59 (m, 2 H) 1.61–1.70 (m, 1 H) 1.78–1.93 (m, 2 H) 2.14 (m, 1 H) 2.35–2.46 (m, 2 H) 3.14–3.29 (m, 2 H) 3.32–3.38 (m, 2 H) 3.54–3.67 (m, 3 H) 3.68–3.77 (m, 1 H) 3.87–3.93 (m, 2 H) 4.01–4.13 (m, 3 H) 4.22–4.28 (m, 3 H) 4.28–4.35 (m, 4 H) 4.36–4.40 (m, 2 H) 7.72 (m, J = 8.1 Hz, 2 H; C_ar_‐H) 7.87 (d, J = 8.1 Hz, 2 H; C_ar_‐H); ^13^C NMR (150 MHz, (C═O)42.3, 43.3, 53.5, 54.0, 79.9, 80.0, 82.0, 85.5, 85.7, 85.8, 85.9, 86.2, 86.4, 127.4 (C_ar_‐H), 135.2 (C_ar_‐H), 135.2 (C_ar_‐H), 136.8 (C_ar_‐H), 139.2, 139.3, 160.1 (C═O), 170.4 (C═O), 173.6 (C═O), 173.9 (C═O), 176.0 (C═O), 176.5 (C═O). HRMS (ESI) m/z: (M‐2H)−2 cald. for C45H69FN6O20Si 529.2087, found 529.2082.

##### 4.1.1.19 Compound 22

Yield: 93%. 1H NMR (600 MHz, methanol‐d4) *δ* ppm 1.07 (m, 18 H; C(CH_3_)_3_) 1.40–1.47 (m, 2 H) 1.50–1.60 (m, 2 H) 1.61–1.70 (m, 1 H) 1.79–1.92 (m, 2 H) 2.14 (m, 1 H) 2.40 (m, 2 H) 3.20–3.28 (m, 3 H) 3.39–3.47 (m, 3 H) 3.68–3.74 (m, 2 H) 3.78–3.85 (m, 1 H) 4.24–4.32 (m, 2 H) 7.74 (d, J = 8.3 Hz, 2 H; C_ar_‐H) 7.85 (d, J = 8.3 Hz, 2 H; C_ar_‐H); ^13^C NMR (150 MHz, methanol‐d4) *δ* ppm 21.0 ( **C** (CH_3_)_3_), 21.1, 23.8, 27.7 (C( **C** H_3_)_3_), 28.9, 29.8, 31.1, 33.1, 39.8, 42.0, 53.5, 53.9, 72.2, 73.6, 78.8, 79.4, 80.0, 127.5 (C_ar_‐H), 135.2 (C_ar_‐H), 135.3 (C_ar_‐H), 136.8 (C_ar_‐H), 139.3, 139.4, 160.1 (C═O), 170.9 (C═O), 172.4 (C═O), 175.9 (C═O), 176.4 (C═O), 176.4 (C═O). HRMS (ESI) m/z: (M‐H)^‐^ cald. for C34H53FN4O13Si 772.3290, found 772.3294.

##### 4.1.1.20 Compound 23

Yield: 95%. 1H NMR (600 MHz, methanol‐d4) *δ* ppm 1.03–1.10 (m, 18 H; C(CH_3_)_3_) 1.38–1.45 (m, 2 H) 1.47–1.68 (m, 3 H) 1.78–1.93 (m, 2 H) 2.14 (m, 1 H) 2.36–2.45 (m, 2 H) 3.18–3.29 (m, 4 H) 3.32–3.50 (m, 7 H) 3.66–3.79 (m, 5 H) 4.25 (dd, J = 8.4, 4.9 Hz, 1 H) 4.31 (dd, J = 8.6, 4.9 Hz, 1 H) 7.74 (m, J = 8.2 Hz, 2 H; C_ar_‐H) 7.86 (m, J = 8.3 Hz, 2 H; C_ar_‐H); ^13^C NMR (150 MHz, methanol‐d4) *δ* ppm 21.0 ( **C** (CH_3_)_3_), 21.1, 23.8, 27.7 (C( **C** H_3_)_3_), 28.8, 29.8, 31.1, 33.1, 39.8, 41.1, 42.0, 53.5, 54.0, 72.1, 72.1, 73.6, 73.7, 78.8, 79.2, 79.6, 79.8, 80.0, 127.5 (C_ar_‐H), 135.3 (C_ar_‐H), 135.3 (C_ar_‐H), 136.8 (C_ar_‐H), 139.2, 139.3, 160.1 (C═O), 170.9 (C═O), 172.4 (C═O), 173.0 (C═O), 176.0 (C═O), 176.5 (C═O). HRMS (ESI) m/z: (M‐2H)−2 cald. for C41H64FN5O18Si 479.6927, found 479.6929.

##### 4.1.1.21 Compound 24

Yield: 90%. 1H NMR (600 MHz, methanol‐d4) *δ* ppm 1.03–1.07 (m, 18 H; C(CH_3_)_3_) 1.40–1.47 (m, 2 H) 1.50–1.60 (m, 2 H) 1.63–1.71 (m, 1 H) 1.80–1.93 (m, 2 H) 2.14 (m, 1 H) 2.36–2.46 (m, 2 H) 3.18–3.29 (m, 5 H) 3.32–3.54 (m, 11 H) 3.64–3.74 (m, 4 H) 3.76–3.80 (m, 3 H) 4.24–4.33 (m, 2 H) 7.73 (d, J = 8.3 Hz, 2 H; C_ar_‐H) 7.86 (d, J = 8.3 Hz, 2 H; C_ar_‐H); ^13^C NMR (150 MHz, methanol‐d4) *δ* ppm 21.0 ( **C** (CH_3_)_3_), 21.1, 23.9, 27.7 (C( **C** H_3_)_3_), 28.8, 29.8, 31.1, 33.1, 39.9, 40.9, 41.3, 42.0, 53.5, 54.0, 71.9, 72.1, 72.2, 73.6, 78.8, 79.3, 79.5, 79.7, 79.8, 80.0, 127.6 (C_ar_‐H), 135.2 (C_ar_‐H), 135.3 (C_ar_‐H), 136.8 (C_ar_‐H), 139.6, 160.2 (C═O), 172.4 (C═O), 172.9 (C═O), 176.0 (C═O), 176.5 (C═O), 176.5 (C═O). HRMS (ESI) m/z: (M‐2H)−2 cald. for C48H75FN6O23Si 574.2246, found 574.2245.

### 4.2 Determination of logP Values

The lipophilicity of the compounds was determined by reversed‐phase HPLC according to the method of Minick et al. [21, 22]. Chromatographic capacity factors (k′) were measured on a C8 reversed‐phase column using mobile phases containing varying methanol concentrations (0.25–0.75, v/v). The organic phase consisted of methanol containing 0.25% (v/v) 1‐octanol, while the aqueous phase comprised octanol‐saturated water containing 0.02 M MOPS buffer and 0.15% (v/v) n‐decylamine (pH 7.4). Log k′ values were linearly extrapolated to 100% aqueous mobile phase (log k′w), and logP values were calculated from a calibration curve generated with reference compounds (p‐anisidine (log*P* = 0.95); phenol (log*P* = 1.46); p‐cresol (log*P* = 1.94); 4‐bromoaniline (log*P* = 2.26); naphthalene (log*P* = 3.30) of known octanol/water partition coefficients.

### 4.3 Radiochemistry

The ^18^F labeling was performed manually using a recently developed method in our laboratory [7]. Aqueous [^18^F]fluoride (1–1.5 GBq) from the cyclotron was first trapped on a quaternary methylammonium cartridge (Waters Sep‐Pak Accell Plus QMA Carbonate, 130 mg) and dried with a stream of air (10 mL). The trapped [^18^F]fluoride was then eluted into a reaction vial in 4 drops using a solution of Kryptofix2.2.2 (10 mg) and K_2_CO_3_ (1 M, 10 μL) in acetonitrile (950 μL) and water (40 μL). The eluate was dried at 90–100 °C for 5 min. After cooling to room temperature, the dried [^18^F]fluoride was transferred with dry acetonitrile (50 μL) into a reaction vial containing 30–50 nmol of radiolabelling precursor dissolved in DMSO (15–20 μL). The reaction mixture was heated at 95 °C for 40 min, followed by purification using HPLC. The collected radiotracer fraction was concentrated under high vacuum, then formulated in isotonic saline to a final ethanol concentration of ≤10%. The total synthesis time, including [^18^F]fluoride drying, was ≈1 h. The method consistently provided moderate radiochemical yields (25%–40%, decay‐corrected to the start of synthesis), with a molar activity of 86 ± 3 GBq/μmol (*n* = 8) and radiochemical purity ≥98%.

### 4.4 Competitive Binding Assay

In vitro competitive binding was analyzed in PSMA‐expressing human prostate adenocarcinoma LNCaP cells in triplicate according to literature [6, 23]. Briefly, the determination of the concentration of half‐maximum inhibition (IC_50_ values) was carried out as a competition against [^18^F]PSMA‐1007 as radioligand using increasing concentrations of compounds **19** **–24** in the range of 0.1 nM to 100 μM. After incubation for 2 h at 37 ºC and several washing steps, the cells were harvested. Counts per minute (cpm) of cell‐associated radioactivity were measured in a HIDEX automated gamma counter (Hidex Oy, Turku, Finland), decay corrected and plotted versus the log of the PSMA inhibitor concentration to give the typical sigmoidal dose–response curves. Graphs were constructed using GraphPad Prism 5.0 (GraphPad Software, San Diego, CA).

### 4.5 Molecular Docking

The coordinates from the X‐ray crystal structure of PSMA (PDB ID: 5O5T, 1.43 Å, PSMA1007 bound in the active site) were retrieved from the RCSB Protein Data Bank server. Compound **21** was built with the builder toolkit of the software package ArgusLab 4.0.1, and energy was minimized using the semiempirical quantum mechanical method PM3. The crystallized ligand was docked first for validating the computational method. The protease structure of PSMA was selected, properly protonated, and geometrically optimized and minimized. The active site water molecules were retained and defined as 20 Å around the ligand.

The molecule to be docked in the enzyme active site was inserted into the workspace carrying the structure of the enzyme. The docking program implements an efficient grid‐based docking algorithm, which approximates an exhaustive search within the free volume of the binding site cavity. The conformational space was measured by the geometry optimization of the flexible ligand (rings are treated as rigid) in combination with the incremental construction of the ligand torsions. Therefore, docking occurred between the flexible parts of the ligand and receptor. The ligand orientation was determined by a shape scoring function based on Ascore and the final positions were ranked by lowest interaction energy values. The intermolecular value is the sum of the energies involved in hydrogen bond interactions, hydrophobic interactions, and van der Waals interactions. Each molecular docking experiment was repeated three times to confirm the reproducibility.

### 4.6 Animal Studies

All animal experiments were carried out in accordance with the guidelines of the Canadian Council on Animal Care (CCAC) and approved by the local animal care committee (Cross Cancer Institute, University of Alberta). PET imaging experiments were carried out in male LNCaP tumor‐bearing nu/nu mice (Charles River Laboratories, Quebec, Canada). LNCaP (15–20 × 10^6^ cells in 200 μL of matrigel/PBS 50/50) were injected into the upper left flank of these mice (20–24 g). Before injecting LNCaP cells, the mice received a 1.0 mg/pellet containing dehydroepiandrosterone in a 60‐day release preparation (Innovative Research of America, Sarasota, FL, USA). The pellet was implanted subcutaneously into the upper right flank in order to provide a constant level of testosterone to support tumor growth of androgen receptor‐positive LNCaP cells. Tumors reached sizes of ≈7x7 mm, which were suitable for PET experiments.

### 4.7 Dynamic PET Imaging

General anesthesia of LNCaP tumor‐bearing mice was induced with inhalation of isoflurane in 40% oxygen/60% nitrogen (gas flow 1 mL/min), and the mice were subsequently fixed in prone position. The body temperature was kept constant at 37 °C for the entire experiment.

The mice were positioned in a prone position into the center of the field of view of an INVEON PET/CT scanner (Siemens Preclinical Solutions, Knoxville, TN). A transmission scan for attenuation correction was not acquired.

The mice were injected with 4–6 MBq of **[** ^**18**^ **F]19** and **[** ^**18**^ **F]21** in 100–150 μL of isotonic NaCl solution (0.9%) through a tail vein catheter. For blocking studies, the animals were predosed with 300 μg of DCFPyL in 50 μL saline about 5 min before radiotracer injection. Data acquisition was performed over 60 min in a 3D list mode. The dynamic list mode data were sorted into sinograms with 54 time frames (10 × 2, 8 × 5, 6 × 10, 6 × 20, 8 × 60, 10 × 120, and 6 × 300 s).

The frames were reconstructed using maximum a posteriori as reconstruction mode. The pixel size was 0.085 × 0.085 × 0.121 mm^3^ (256 × 256 × 63), and the resolution in the center of the field of view was 1.8 mm. No correction for partial volume effects was applied. The image files were processed using the ROVER v 2.0.51 software (ABX GmbH, Radeberg, Germany). Masks defining 3D regions of interest (ROI) were set and defined by thresholding. Mean standardized uptake values (SUVmean as activity/mL tissue)/(injected activity/body weight), mL/g, were determined for each ROI. TACs were generated for the dynamic scans. All semiquantified PET data are presented as means ± SEM from n experiments.

## Funding

The authors have nothing to report.

## Conflicts of Interest

The authors declare no conflicts of interest.

## Supporting information

Supplementary Material

## Data Availability

The data that support the findings of this study are available on request from the corresponding author. The data are not publicly available due to privacy or ethical restrictions.
