# Supplementary material for: Sugar Amino Acid Linkers for the Design of [18F]SiFA‐PSMA Radioligands
Source: ChemMedChem. 2026 Aug 12;21(15):e70436. doi: 10.1002/cmdc.70436 (PMC13469581; doi:10.1002/cmdc.70436)
Supplement: Supplementary file 1 [file CMDC-21-e70436-s001.pdf]

Sugar amino acids for the design of [^{18}F]SiFA-PSMA radioligands

Supplementary Information

Narendar R. Gade[a], Melinda Wuest[a], Justin J. Bailey[a], Atul Bhardwaj[a], Ralf Schirrmacher[a,b], and Frank Wuest[a,b,c],*

[a] Department of Oncology, Cross Cancer Institute, University of Alberta, Edmonton, AB, T6G 1Z2 (Canada)
[b] Department of Chemistry, University of Alberta, 11227 Saskatchewan Drive, Edmonton, AB, T6G 2G2 (Canada)
[c] Cancer Research Institute of Northern Alberta, 87 Ave and 112 St, Edmonton, AB, T6G 2E1 (Canada)

^{1}H and ^{13}C NMR spectra of synthesized compounds……………………..……………………2-19

HPLC traces of radioligands **[^{18}F]19** and **[^{18}F]21**………………………..……………………20-21

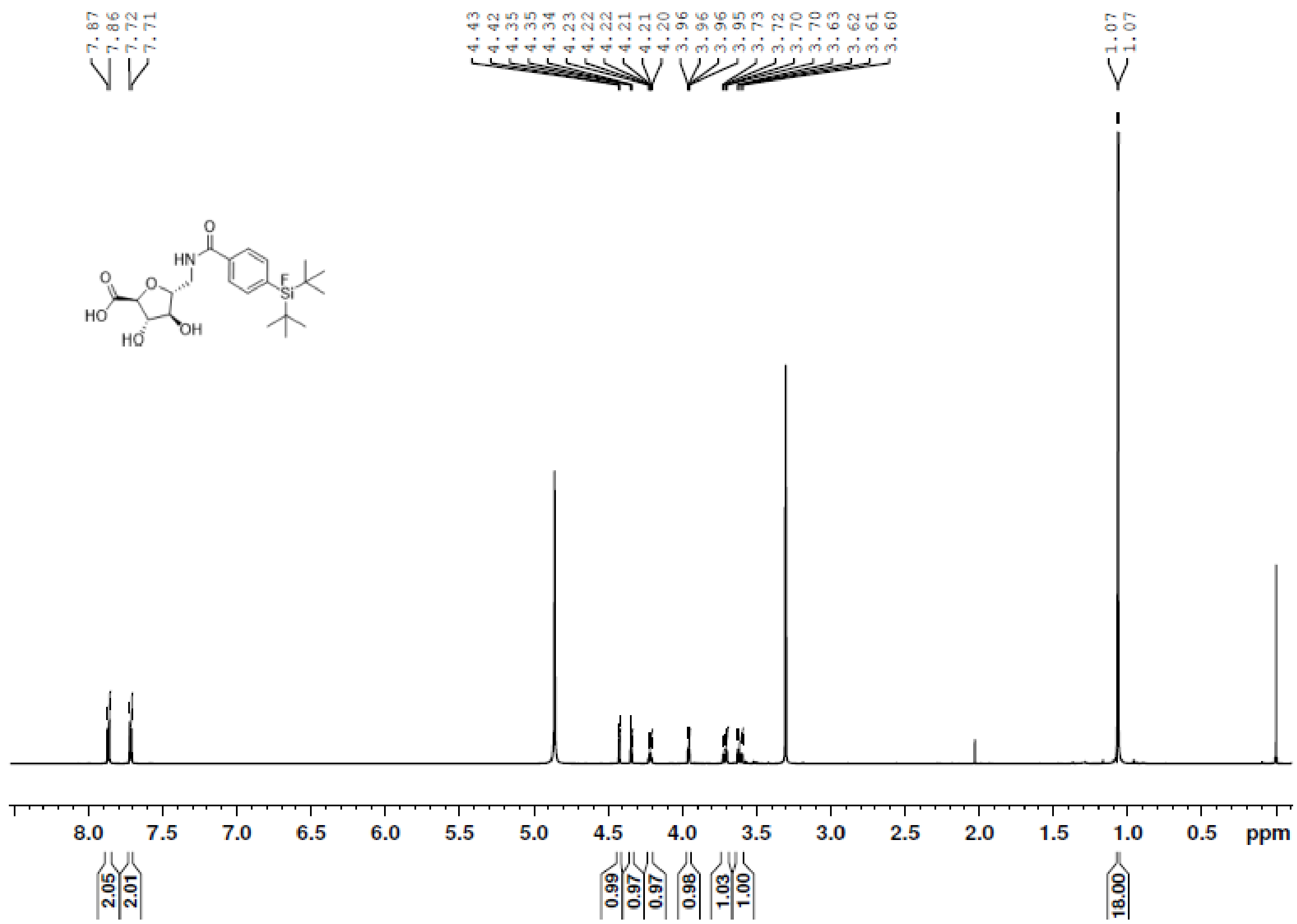
7.87
7.86
7.72
7.71
4.43
4.42
4.35
4.35
4.34
4.23
4.22
4.22
4.21
4.21
4.20
3.96
3.96
3.96
3.95
3.73
3.72
3.70
3.70
3.63
3.62
3.61
3.60
1.07
1.07
HN
O
F
Si
O
HO
HO
OH
O
8.0 7.5 7.0 6.5 6.0 5.5 5.0 4.5 4.0 3.5 3.0 2.5 2.0 1.5 1.0 0.5 ppm
2.05
2.01
0.99
0.97
0.97
0.98
1.03
1.00
18.00

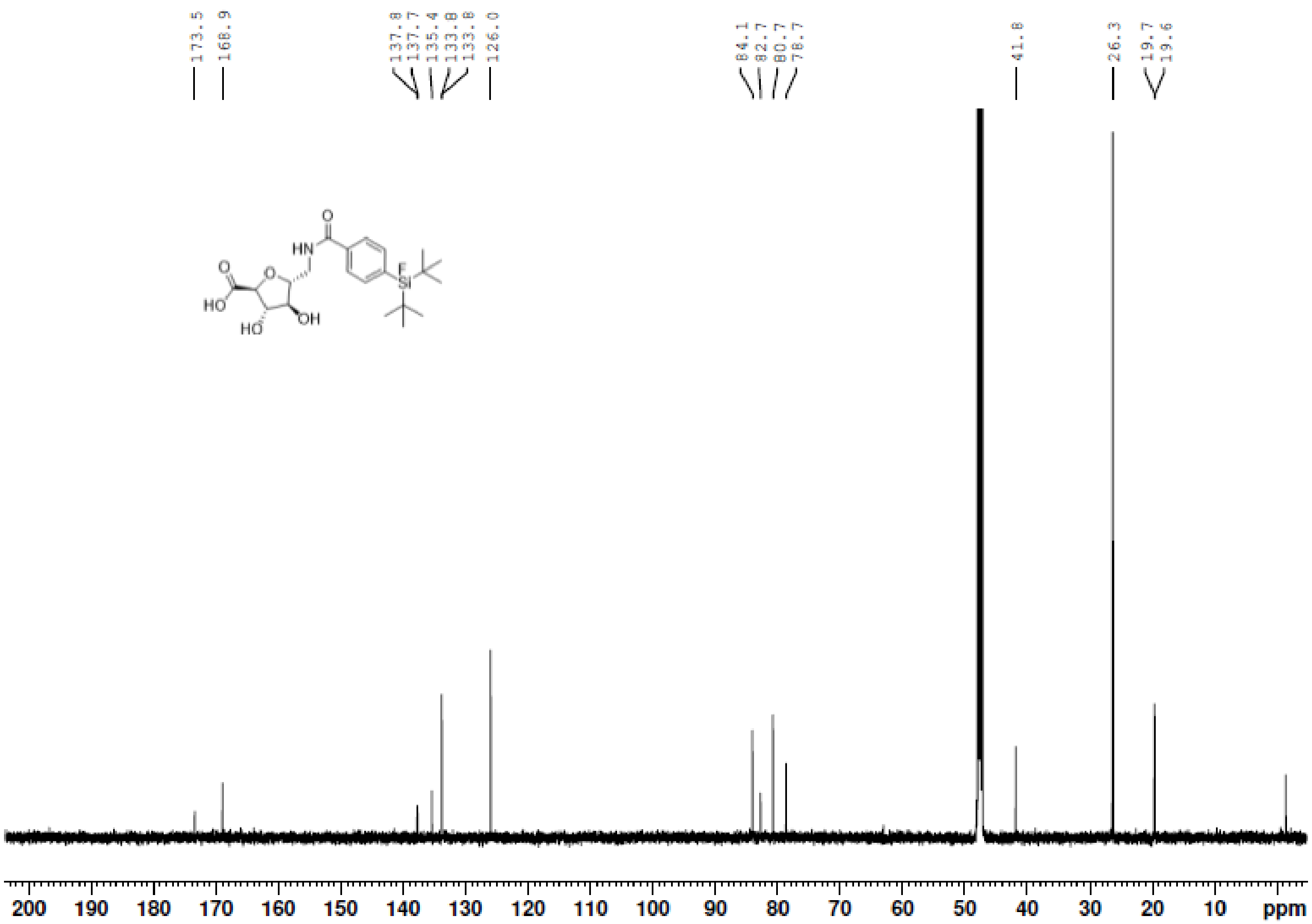
173.5
168.9
137.8
137.7
135.4
133.8
133.8
126.0
84.1
82.7
80.7
78.7
41.8
26.3
19.7
19.6
200 190 180 170 160 150 140 130 120 110 100 90 80 70 60 50 40 30 20 10 ppm

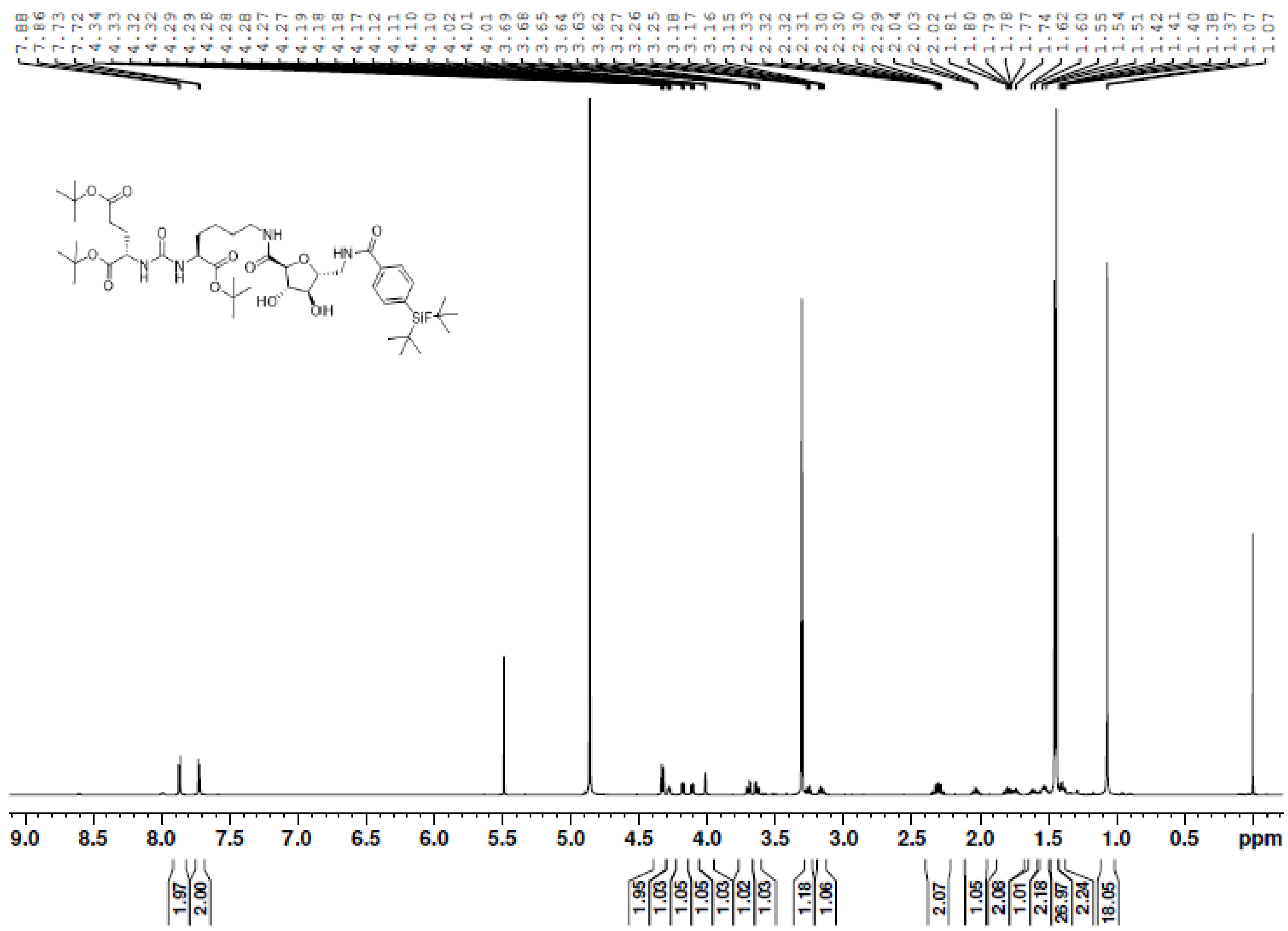
7.88 7.86 7.73 7.72 4.34 4.33 4.32 4.32 4.29 4.29 4.28 4.28 4.27 4.27 4.19 4.18 4.18 4.17 4.12 4.11 4.10 4.10 4.02 4.01 4.01 3.69 3.68 3.65 3.64 3.63 3.62 3.27 3.26 3.25 3.18 3.17 3.16 3.15 2.33 2.32 2.32 2.31 2.30 2.30 2.29 2.04 2.03 2.02 1.81 1.80 1.79 1.78 1.77 1.74 1.62 1.60 1.55 1.54 1.51 1.42 1.41 1.40 1.38 1.37 1.07 1.07
9.0 8.5 8.0 7.5 7.0 6.5 6.0 5.5 5.0 4.5 4.0 3.5 3.0 2.5 2.0 1.5 1.0 0.5 ppm
1.97 2.00 1.95 1.03 1.05 1.05 1.03 1.02 1.03 1.18 1.06 2.07 1.05 2.08 1.01 2.18 26.97 2.24 18.05

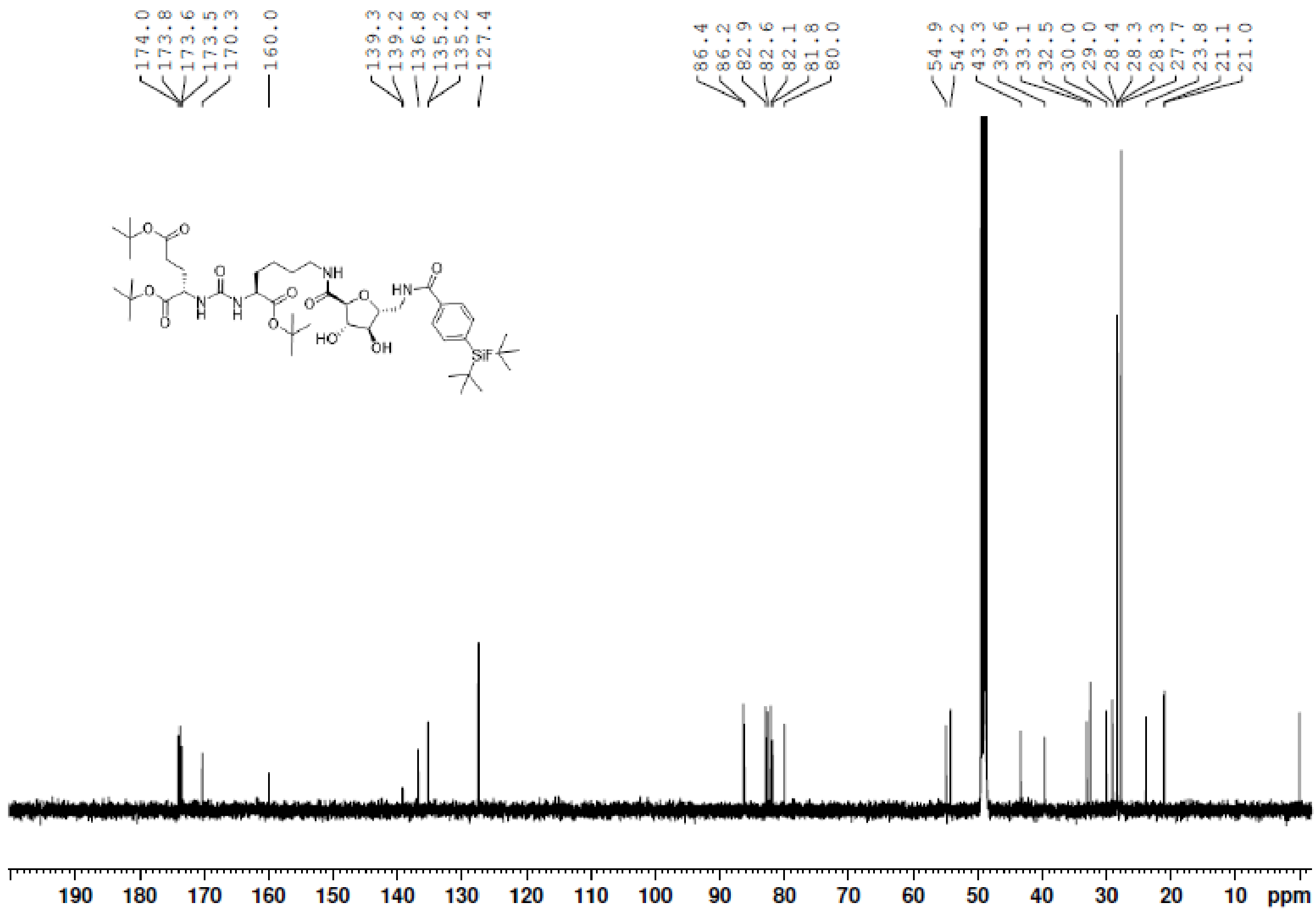
174.0
173.8
173.6
173.5
170.3
160.0
139.3
139.2
136.8
135.2
135.2
127.4
86.4
86.2
82.9
82.6
82.1
81.8
80.0
54.9
54.2
43.3
39.6
33.1
32.5
30.0
29.0
28.4
28.3
28.3
27.7
23.8
21.1
21.0
NH
HN
HO
OH
Si
F
190 180 170 160 150 140 130 120 110 100 90 80 70 60 50 40 30 20 10 ppm

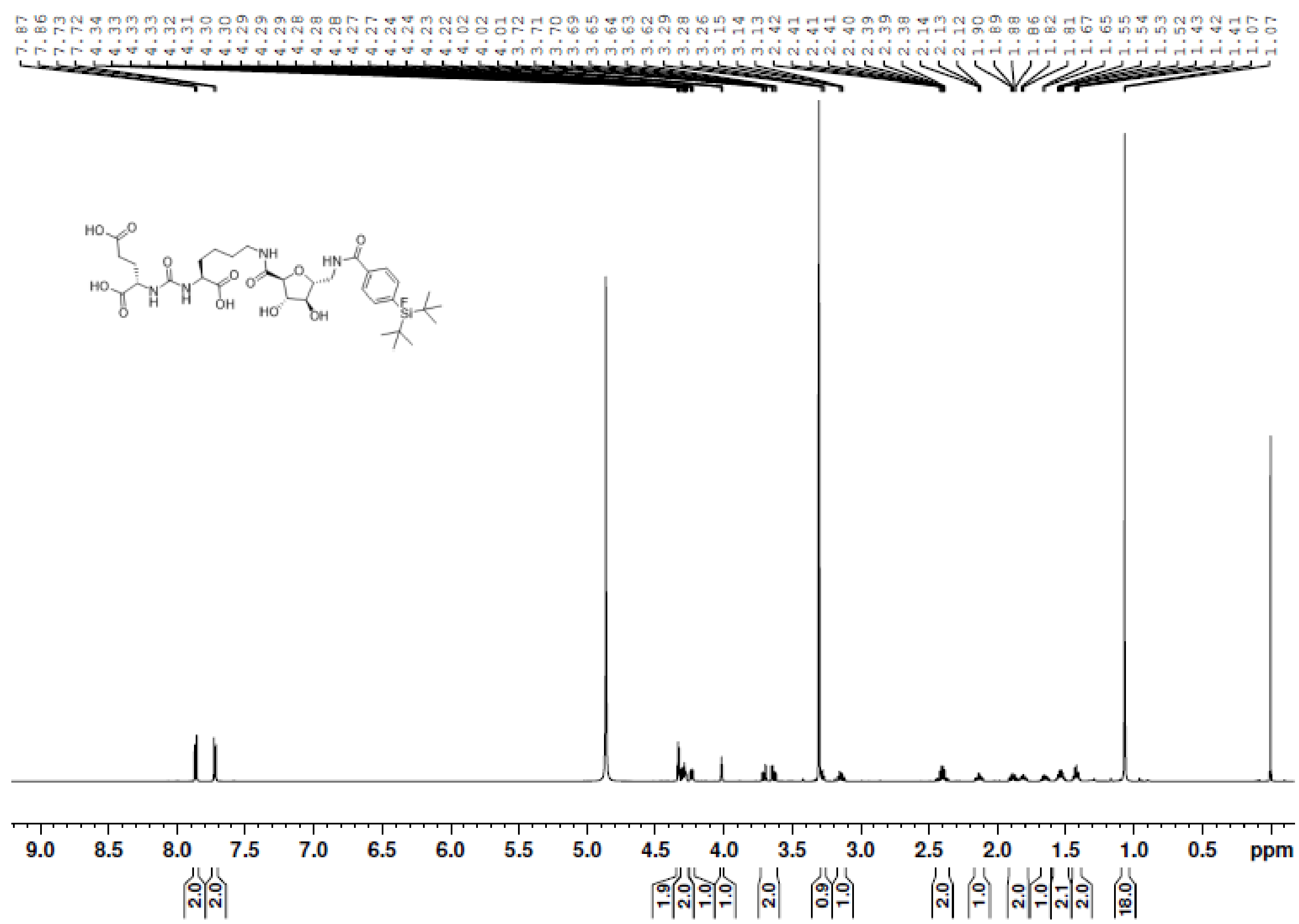
7.87
7.86
7.73
7.72
4.34
4.33
4.33
4.33
4.32
4.31
4.30
4.30
4.29
4.29
4.28
4.28
4.28
4.27
4.27
4.24
4.24
4.23
4.22
4.22
4.02
4.01
3.72
3.71
3.70
3.69
3.65
3.64
3.63
3.62
3.29
3.28
3.26
3.15
3.14
3.13
2.42
2.41
2.41
2.40
2.39
2.39
2.38
2.14
2.13
2.12
1.90
1.89
1.88
1.86
1.82
1.81
1.67
1.65
1.55
1.54
1.53
1.52
1.43
1.42
1.41
1.07
1.07
HO
OH
NH
HN
Si
F
9.0 8.5 8.0 7.5 7.0 6.5 6.0 5.5 5.0 4.5 4.0 3.5 3.0 2.5 2.0 1.5 1.0 0.5 ppm
2.0
2.0
1.9
2.0
1.0
1.0
2.0
0.9
1.0
2.0
1.0
2.0
1.0
2.1
2.0
18.0

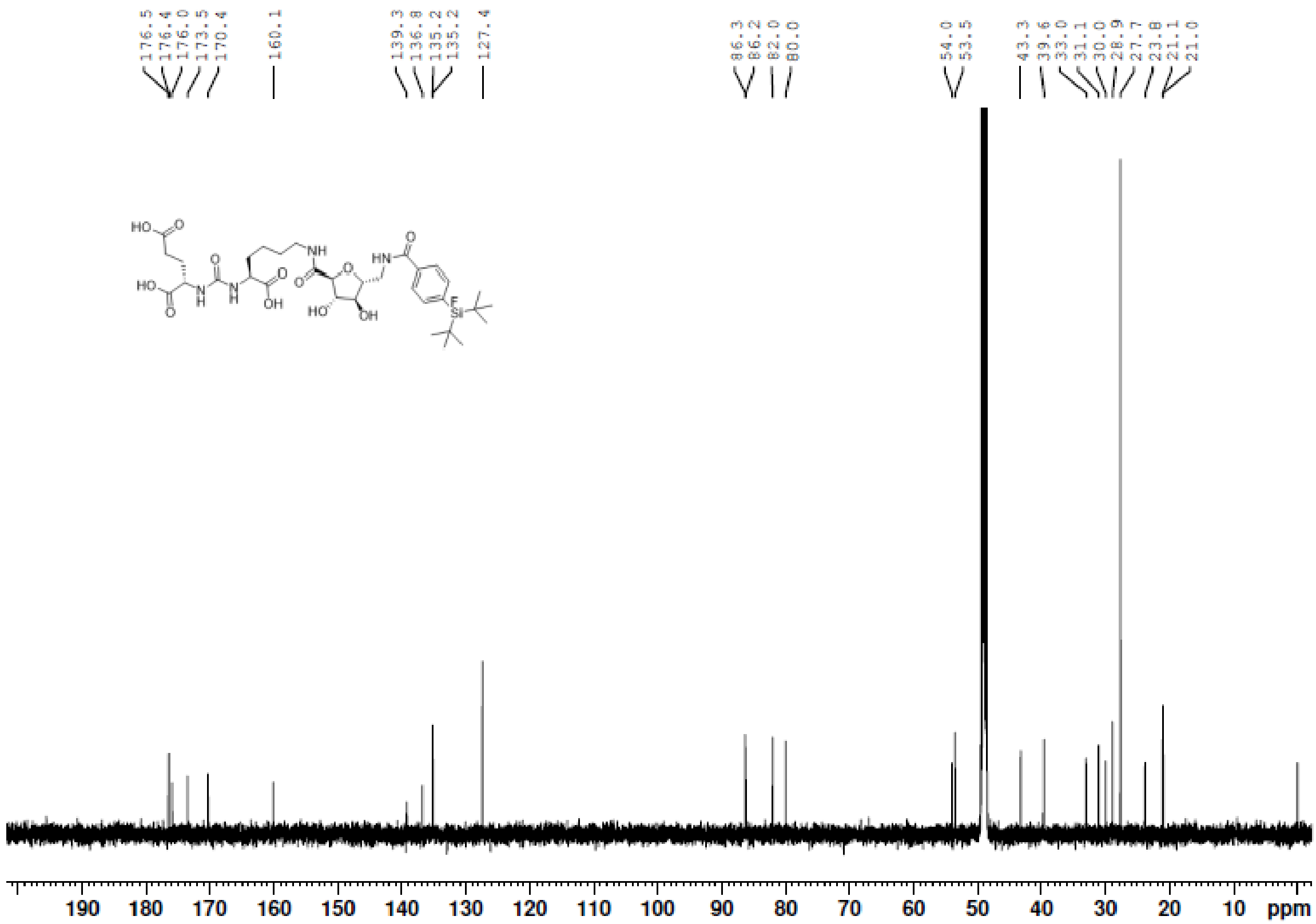
176.5 176.4 176.0 173.5 170.4 160.1 139.3 136.8 135.2 135.2 127.4 86.3 86.2 82.0 80.0 54.0 53.5 43.3 39.6 33.0 31.1 30.0 28.9 27.7 23.8 21.1 21.0
190 180 170 160 150 140 130 120 110 100 90 80 70 60 50 40 30 20 10 ppm

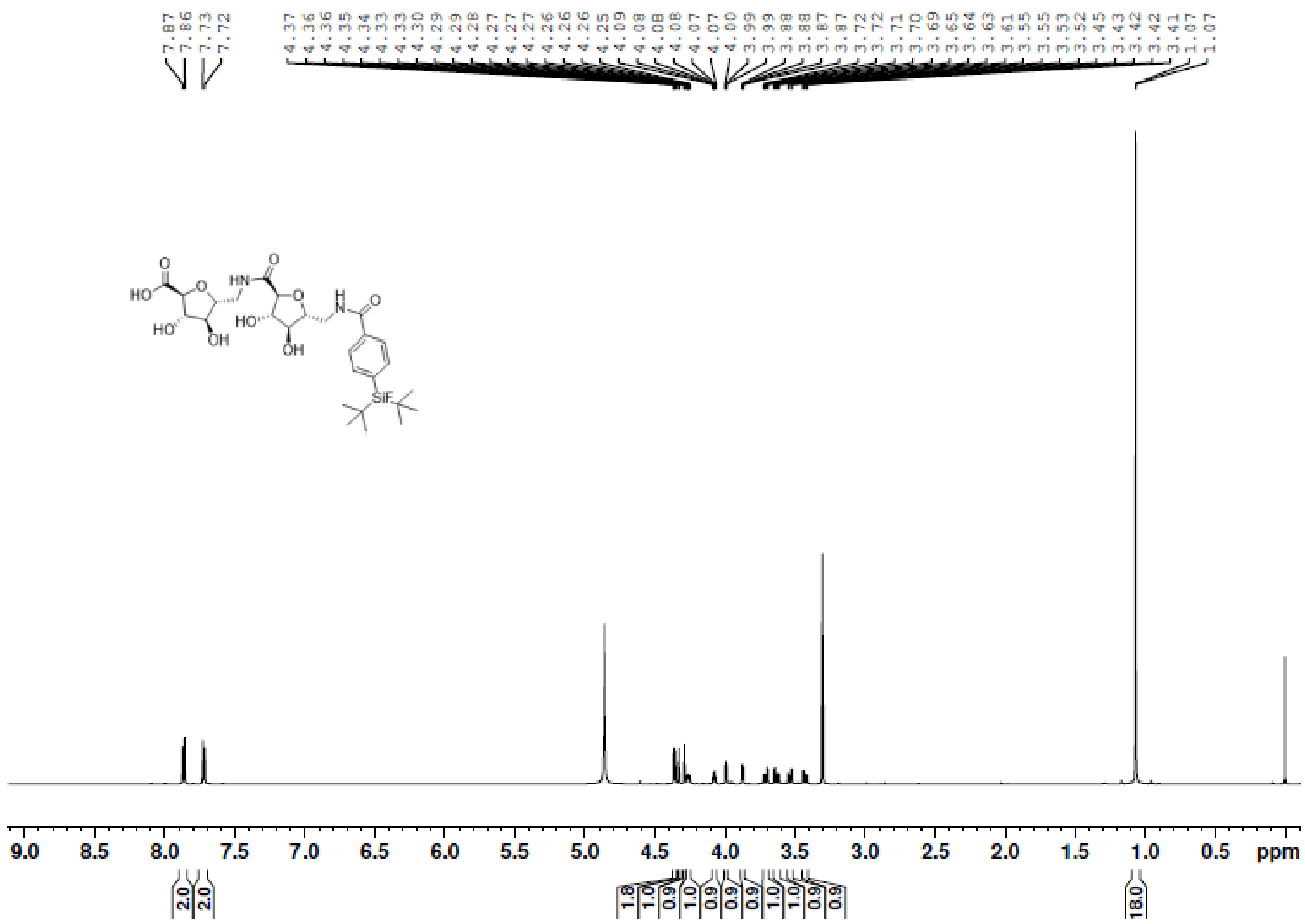
7.87 7.86 7.73 7.72 4.37 4.36 4.35 4.34 4.33 4.30 4.29 4.29 4.28 4.27 4.27 4.26 4.26 4.25 4.09 4.08 4.08 4.07 4.07 4.00 3.99 3.99 3.88 3.88 3.87 3.72 3.72 3.71 3.70 3.69 3.65 3.64 3.63 3.61 3.55 3.53 3.52 3.45 3.43 3.42 3.41 1.07 1.07
9.0 8.5 8.0 7.5 7.0 6.5 6.0 5.5 5.0 4.5 4.0 3.5 3.0 2.5 2.0 1.5 1.0 0.5 ppm
2.0 2.0 1.8 1.0 0.9 1.0 0.9 0.9 0.9 1.0 1.0 0.9 0.9 18.0

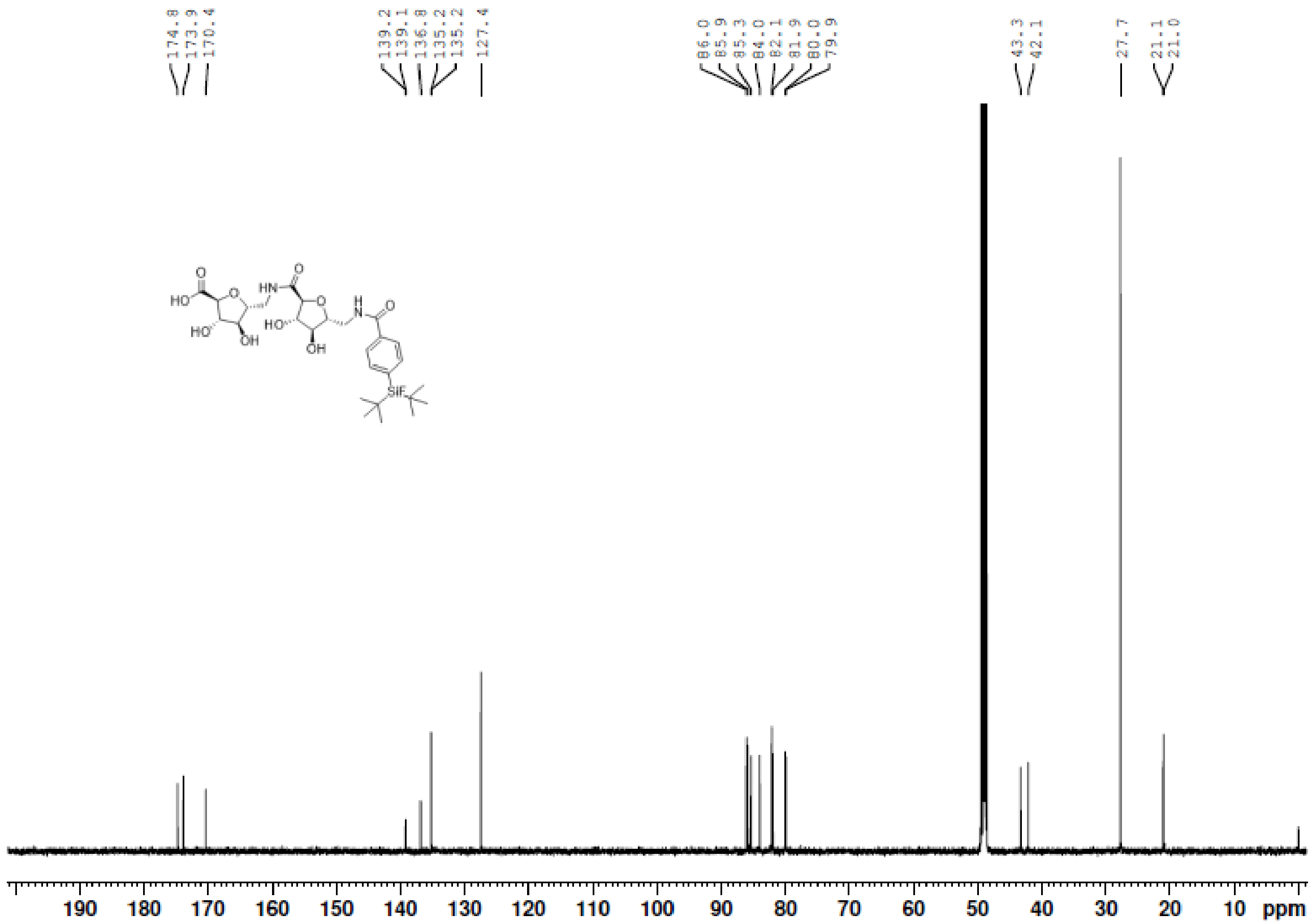
174.8
173.9
170.4
139.2
139.1
136.8
135.2
135.2
127.4
86.0
85.9
85.3
84.0
82.1
81.9
80.0
79.9
43.3
42.1
27.7
21.1
21.0
190 180 170 160 150 140 130 120 110 100 90 80 70 60 50 40 30 20 10 ppm

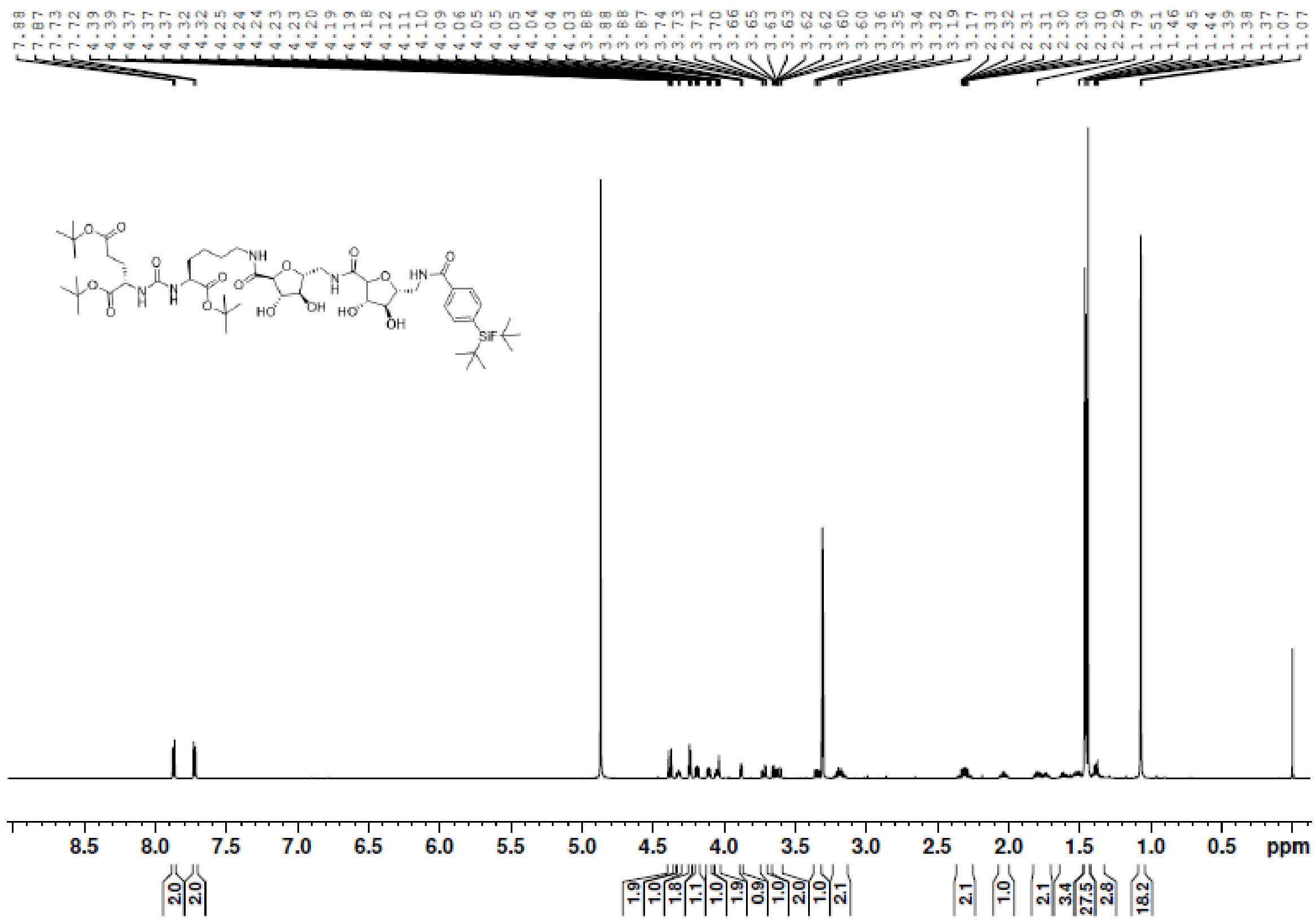
7.88 7.87 7.73 7.72 4.39 4.39 4.37 4.37 4.32 4.32 4.25 4.24 4.24 4.23 4.23 4.20 4.19 4.19 4.18 4.12 4.11 4.10 4.09 4.06 4.05 4.05 4.05 4.04 4.04 4.03 3.88 3.88 3.87 3.74 3.73 3.71 3.70 3.66 3.65 3.63 3.63 3.62 3.62 3.60 3.60 3.36 3.35 3.34 3.32 3.19 3.17 2.33 2.32 2.31 2.31 2.30 2.30 2.29 1.79 1.51 1.46 1.45 1.44 1.39 1.38 1.37 1.07 1.07
8.5 8.0 7.5 7.0 6.5 6.0 5.5 5.0 4.5 4.0 3.5 3.0 2.5 2.0 1.5 1.0 0.5 ppm
2.0 2.0 1.9 1.0 1.8 1.1 1.0 1.9 0.9 1.0 2.0 1.0 2.1 2.1 1.0 2.1 3.4 27.5 2.8 18.2

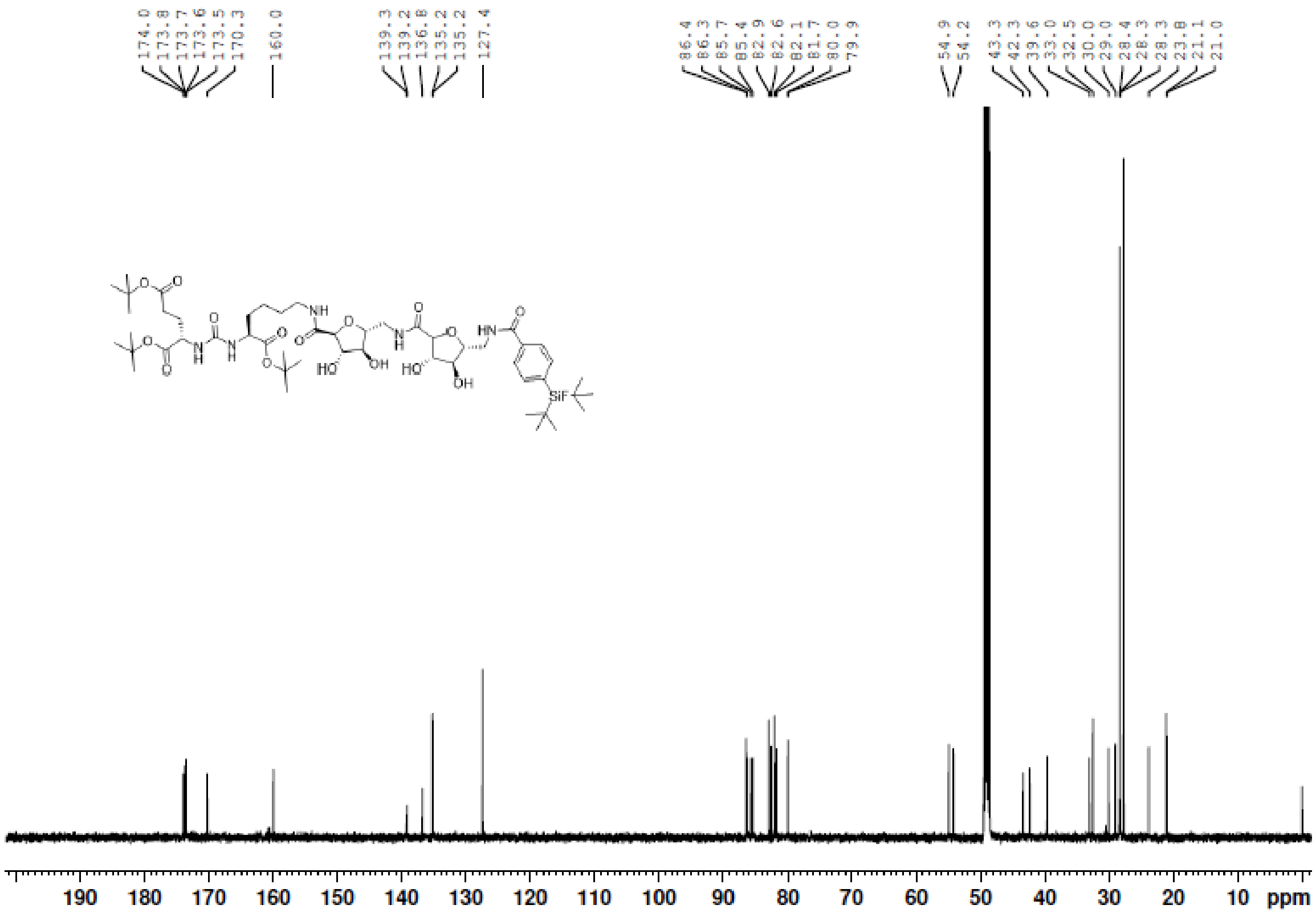
174.0 173.8 173.7 173.6 173.5 170.3 160.0 139.3 139.2 136.8 135.2 135.2 127.4 86.4 86.3 85.7 85.4 82.9 82.6 82.1 81.7 80.0 79.9 54.9 54.2 43.3 42.3 39.6 33.0 32.5 30.0 29.0 28.4 28.3 23.8 21.1 21.0
190 180 170 160 150 140 130 120 110 100 90 80 70 60 50 40 30 20 10 ppm

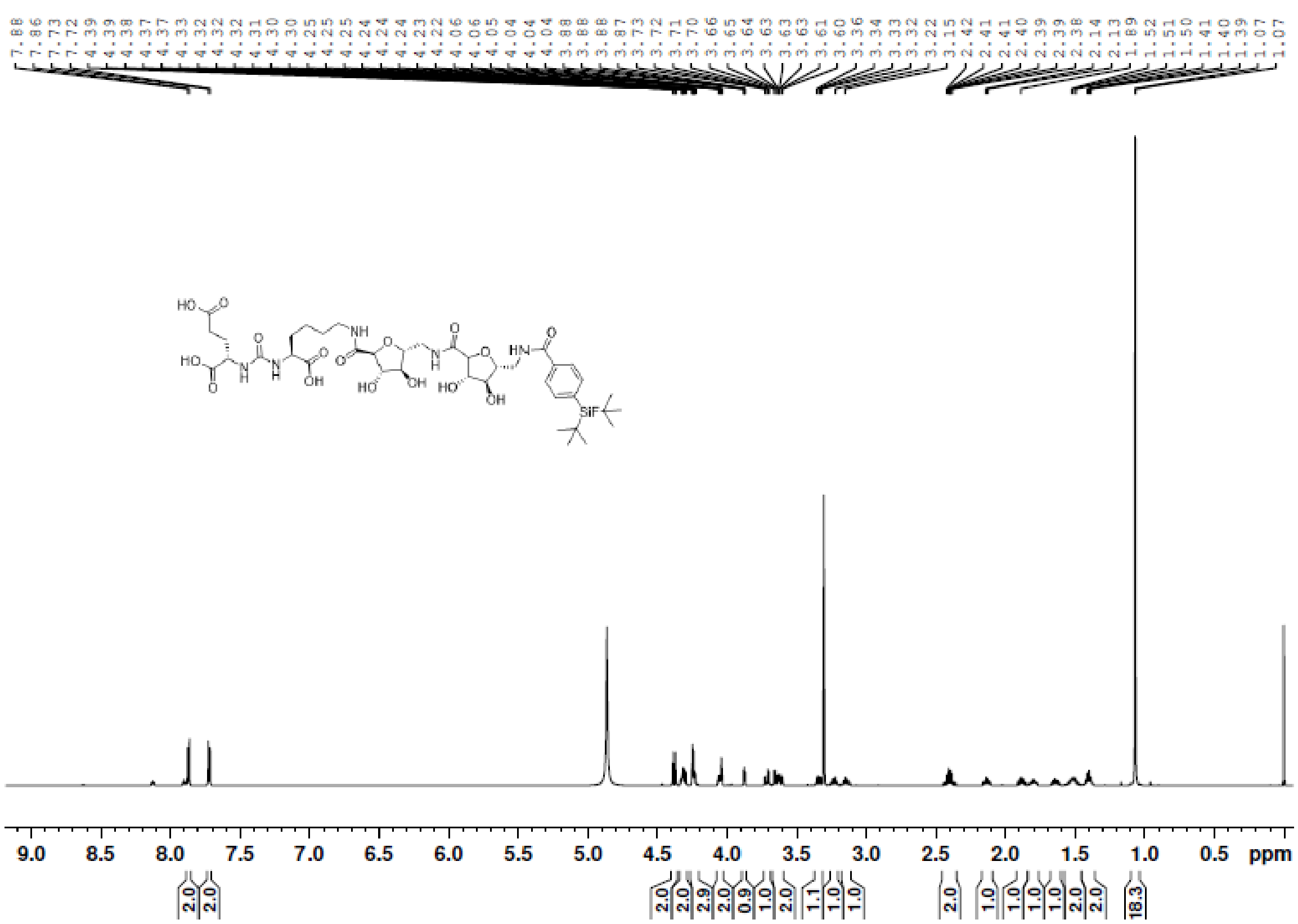
7.88 7.86 7.73 7.72 4.39 4.39 4.38 4.37 4.37 4.33 4.32 4.32 4.31 4.30 4.30 4.25 4.25 4.25 4.24 4.24 4.24 4.23 4.22 4.06 4.06 4.05 4.04 4.04 4.04 3.88 3.88 3.87 3.73 3.72 3.71 3.70 3.66 3.65 3.64 3.63 3.63 3.63 3.61 3.60 3.36 3.34 3.33 3.32 3.22 3.15 2.42 2.41 2.40 2.39 2.39 2.38 2.14 2.13 1.89 1.52 1.51 1.50 1.41 1.40 1.39 1.07 1.07
9.0 8.5 8.0 7.5 7.0 6.5 6.0 5.5 5.0 4.5 4.0 3.5 3.0 2.5 2.0 1.5 1.0 0.5 ppm

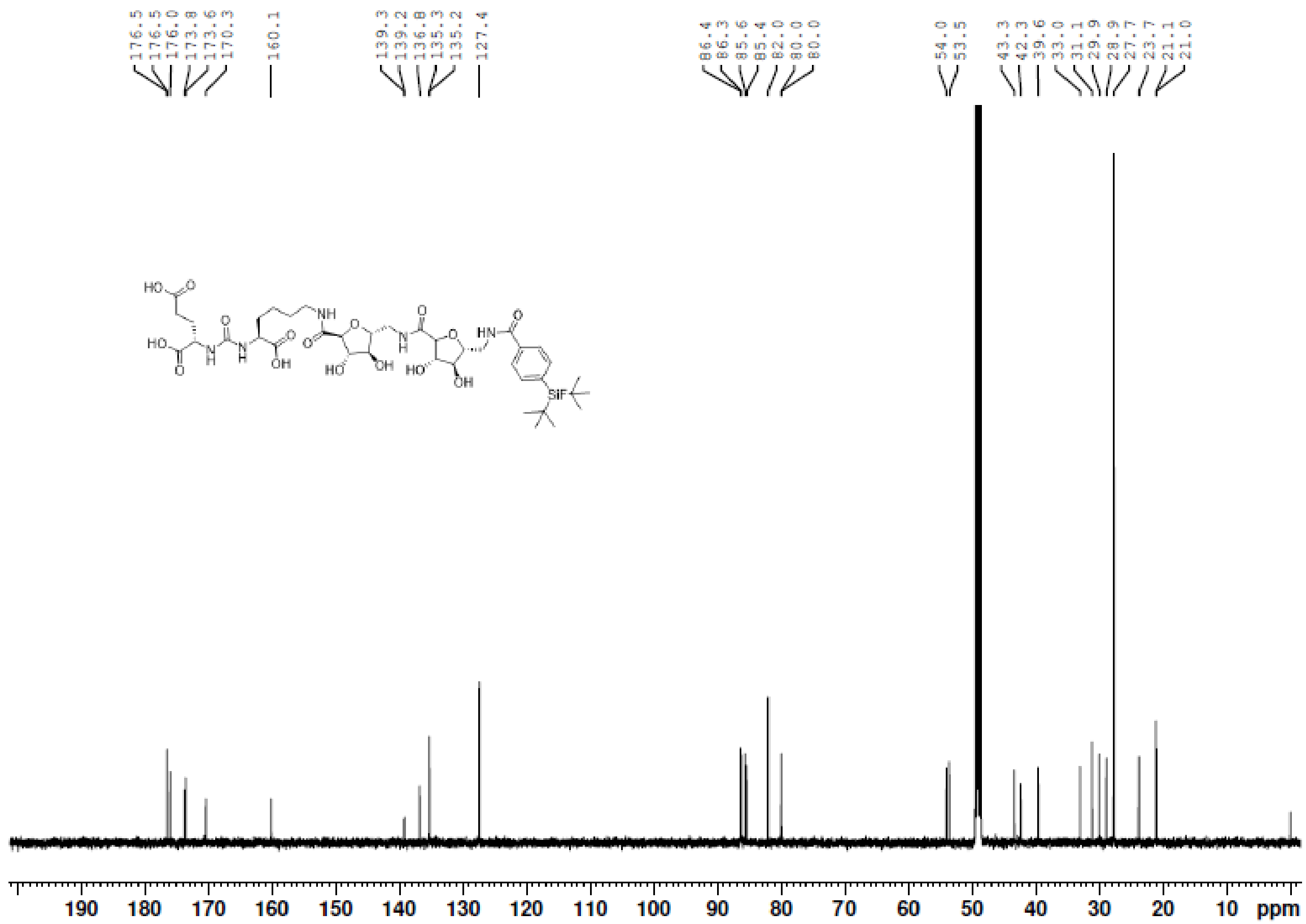
176.5 176.5 176.0 173.8 173.6 170.3
160.1
139.3 139.2 136.8 135.3 135.2 127.4
86.4 86.3 85.6 85.4 82.0 80.0 80.0
54.0 53.5
43.3 42.3 39.6 33.0 31.1 29.9 28.9 27.7 23.7 21.1 21.0
190 180 170 160 150 140 130 120 110 100 90 80 70 60 50 40 30 20 10 ppm

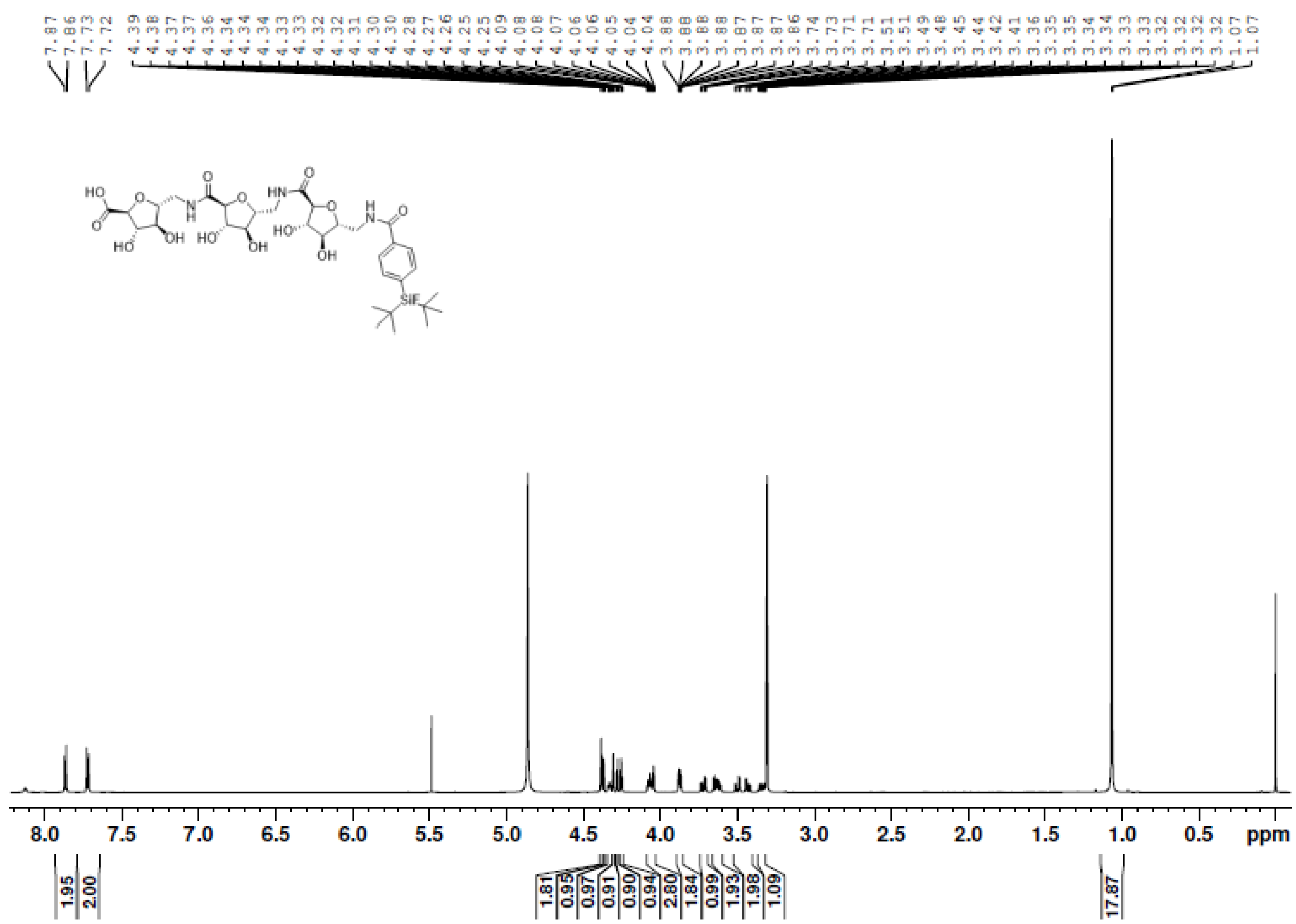
7.87 7.86 7.73 7.72
4.39 4.38 4.37 4.36 4.34 4.34 4.33 4.33 4.32 4.32 4.31 4.30 4.28 4.27 4.26 4.25 4.09 4.08 4.08 4.07 4.06 4.05 4.04 4.04 3.88 3.88 3.88 3.87 3.87 3.86 3.74 3.73 3.71 3.71 3.51 3.51 3.49 3.48 3.45 3.44 3.42 3.41 3.36 3.35 3.35 3.34 3.33 3.33 3.32 3.32 3.32 1.07 1.07
8.0 7.5 7.0 6.5 6.0 5.5 5.0 4.5 4.0 3.5 3.0 2.5 2.0 1.5 1.0 0.5 ppm
1.95 2.00
1.81 0.95 0.97 0.91 0.90 0.94 2.80 1.84 0.99 1.93 1.98 1.09
17.87

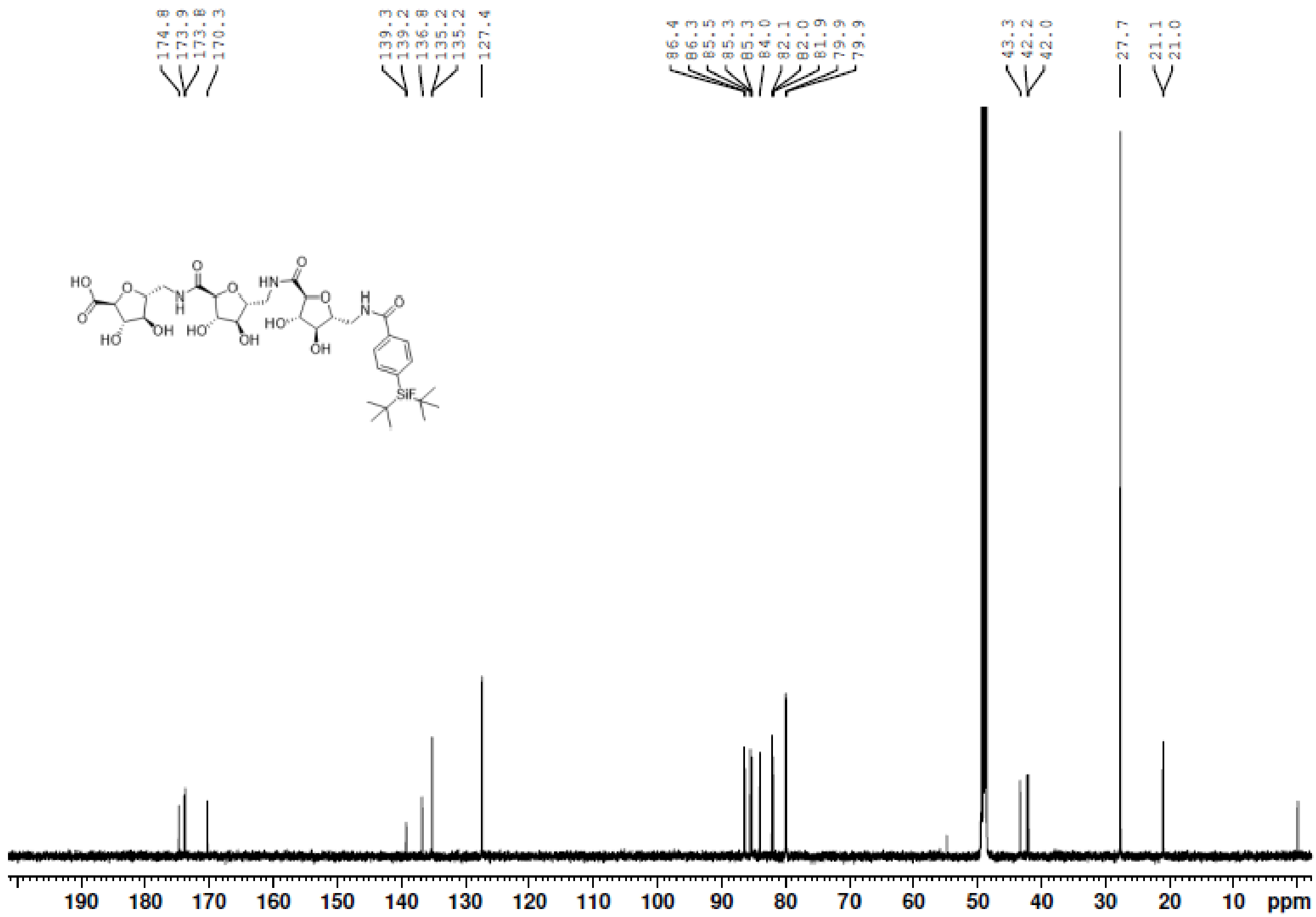
174.8
173.9
173.8
170.3
139.3
139.2
136.8
135.2
135.2
127.4
86.4
86.3
85.5
85.3
85.3
84.0
82.1
82.0
81.9
79.9
79.9
43.3
42.2
42.0
27.7
21.1
21.0
190 180 170 160 150 140 130 120 110 100 90 80 70 60 50 40 30 20 10 ppm

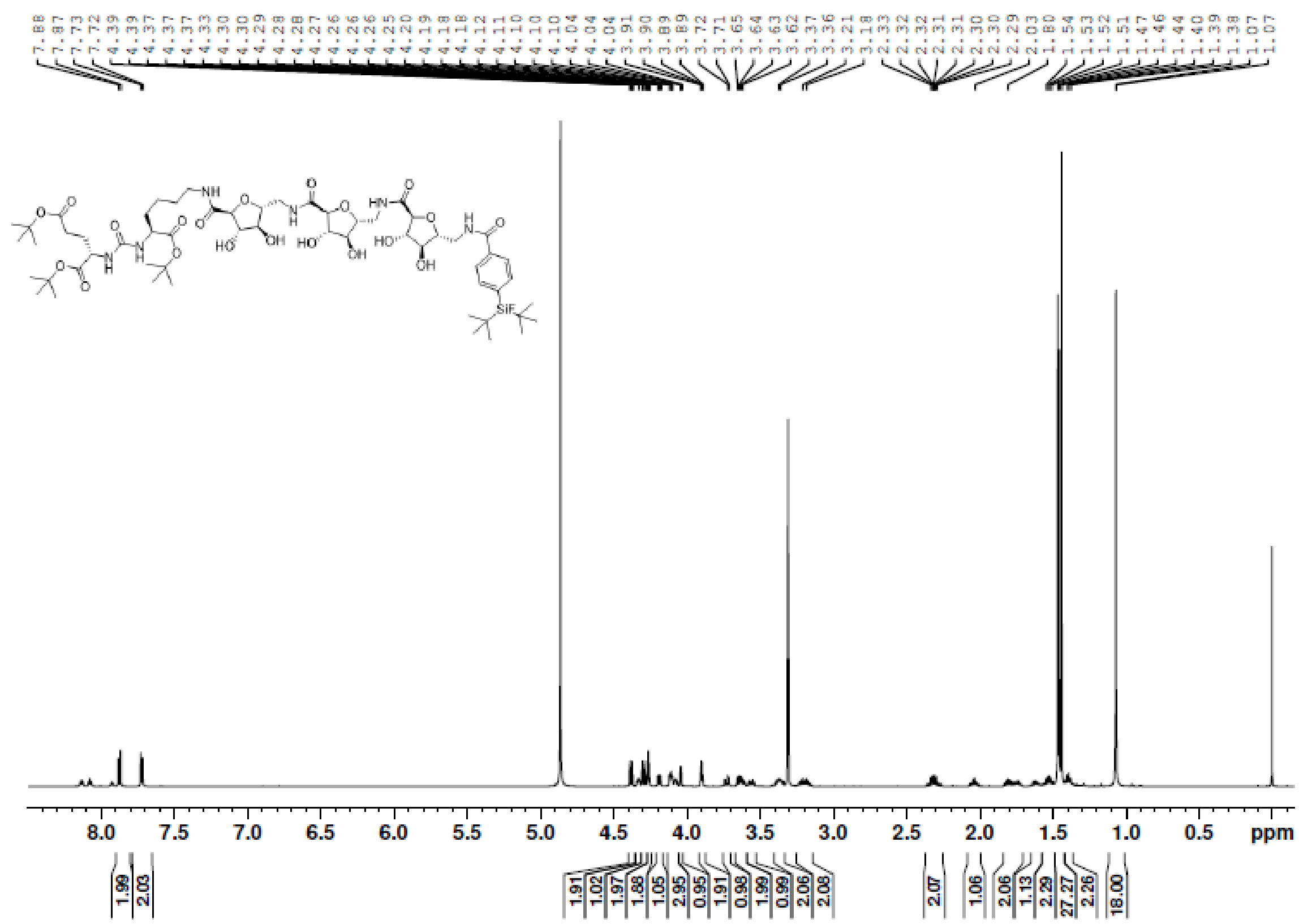
7.88 7.87 7.73 7.72 4.39 4.39 4.37 4.37 4.37 4.33 4.30 4.30 4.29 4.28 4.28 4.27 4.26 4.26 4.25 4.20 4.19 4.18 4.18 4.12 4.11 4.10 4.10 4.10 4.04 4.04 4.04 3.91 3.90 3.89 3.89 3.72 3.71 3.65 3.64 3.63 3.62 3.37 3.36 3.21 3.18 2.33 2.32 2.31 2.31 2.30 2.30 2.29 2.03 1.80 1.54 1.53 1.52 1.51 1.47 1.46 1.44 1.40 1.39 1.38 1.07 1.07
8.0 7.5 7.0 6.5 6.0 5.5 5.0 4.5 4.0 3.5 3.0 2.5 2.0 1.5 1.0 0.5 ppm
1.99 2.03 1.91 1.02 1.97 1.88 1.05 2.95 0.95 1.91 0.98 1.99 0.99 2.06 2.08 2.07 1.06 2.06 1.13 2.29 27.27 2.26 18.00

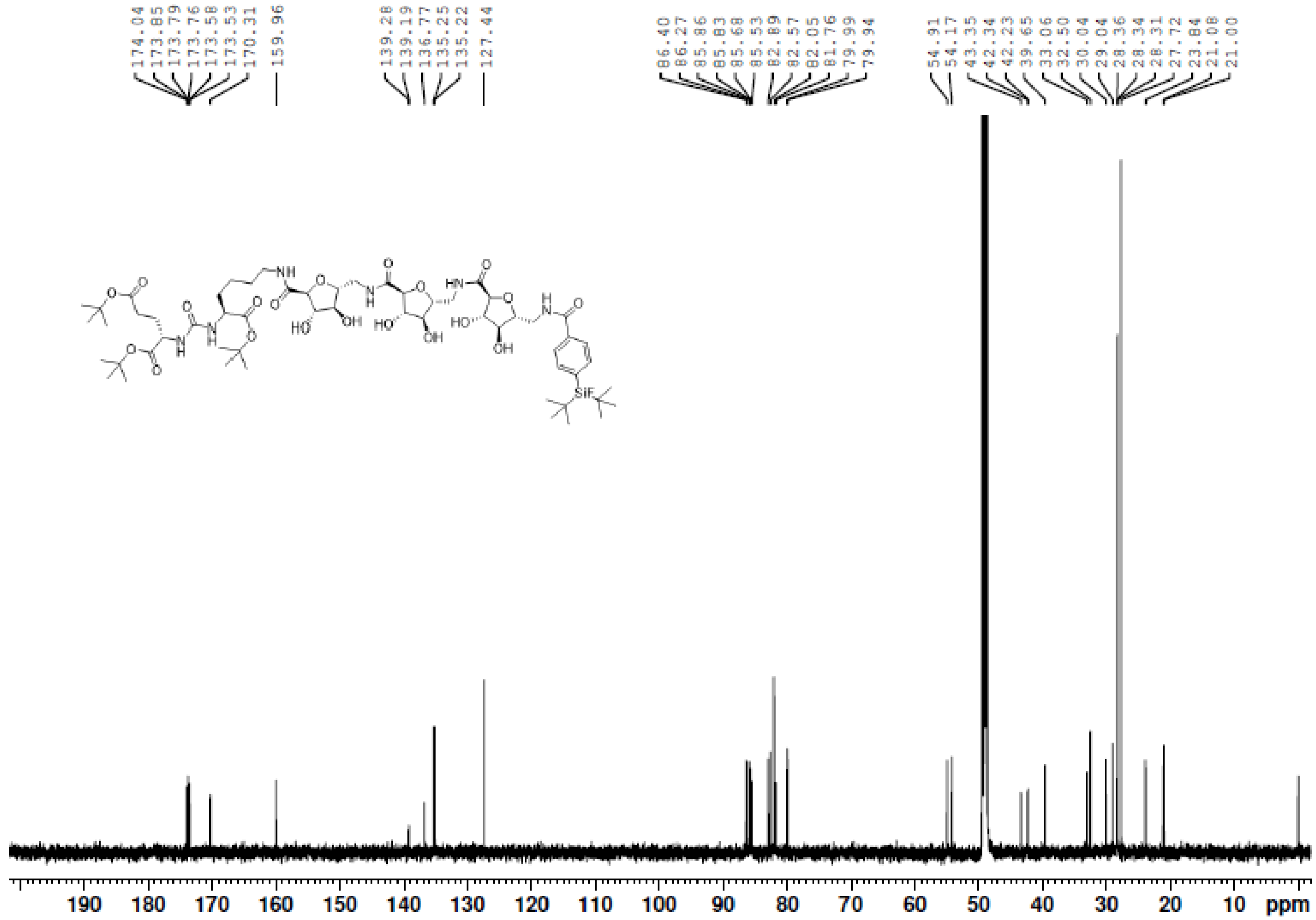

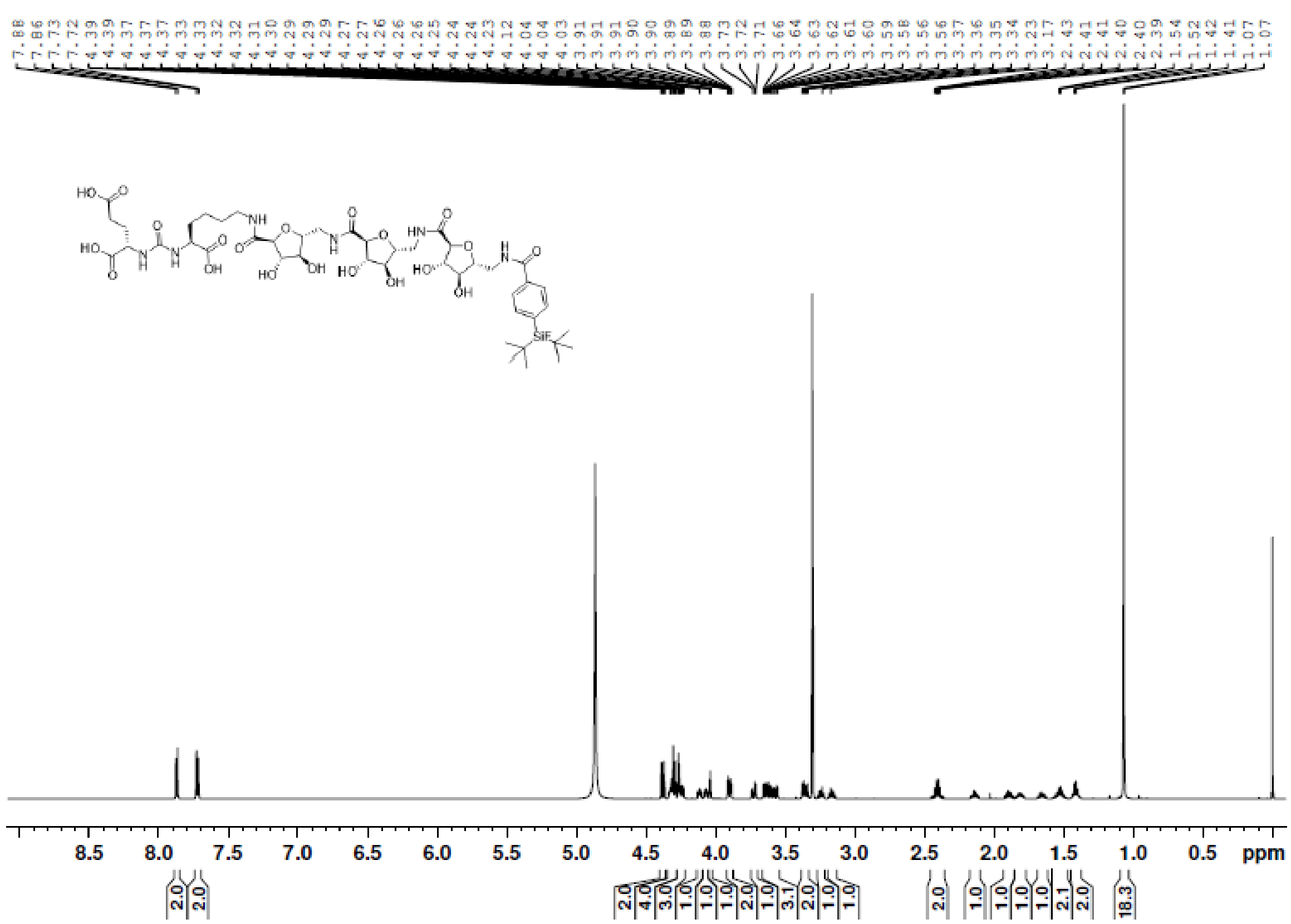

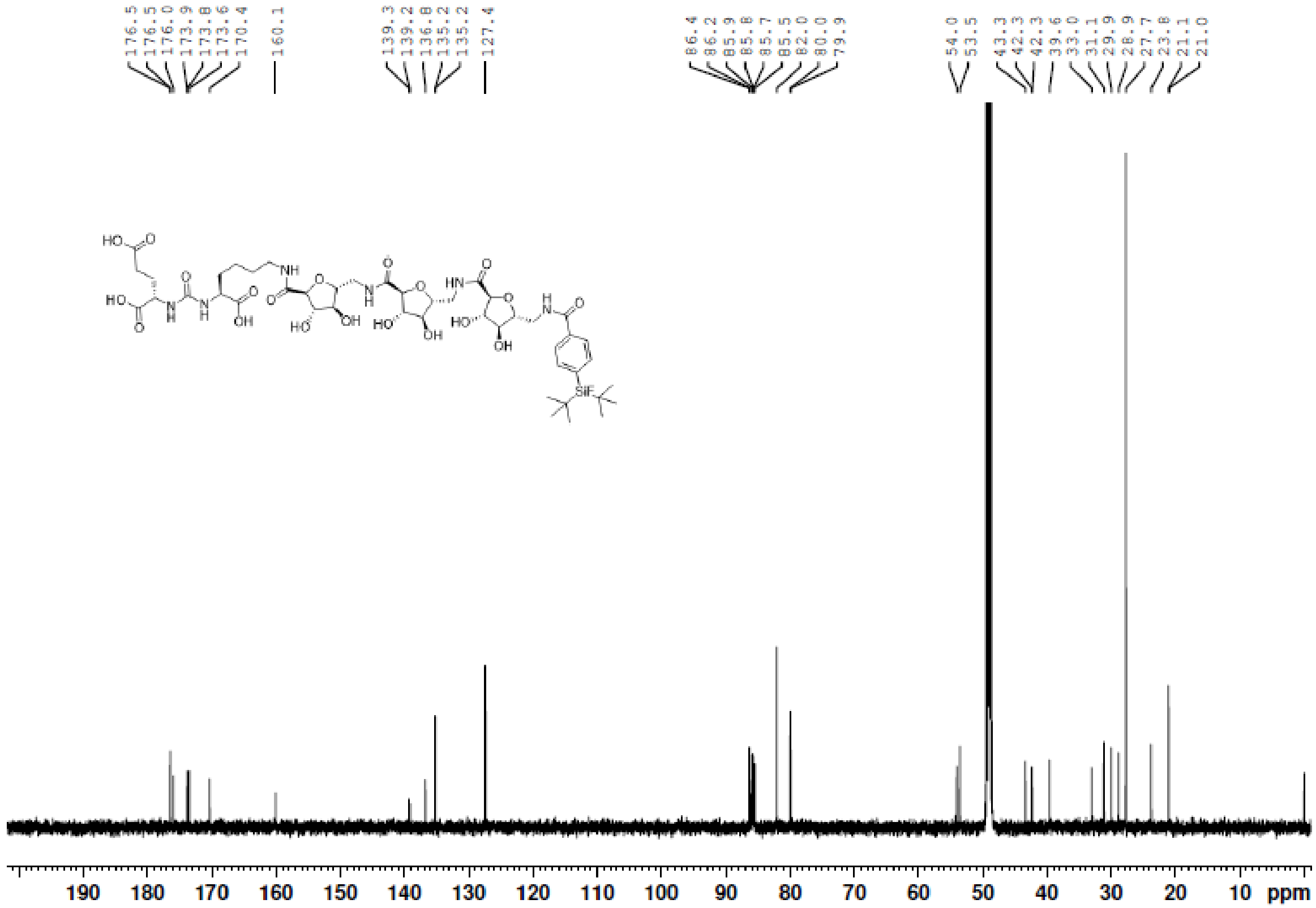

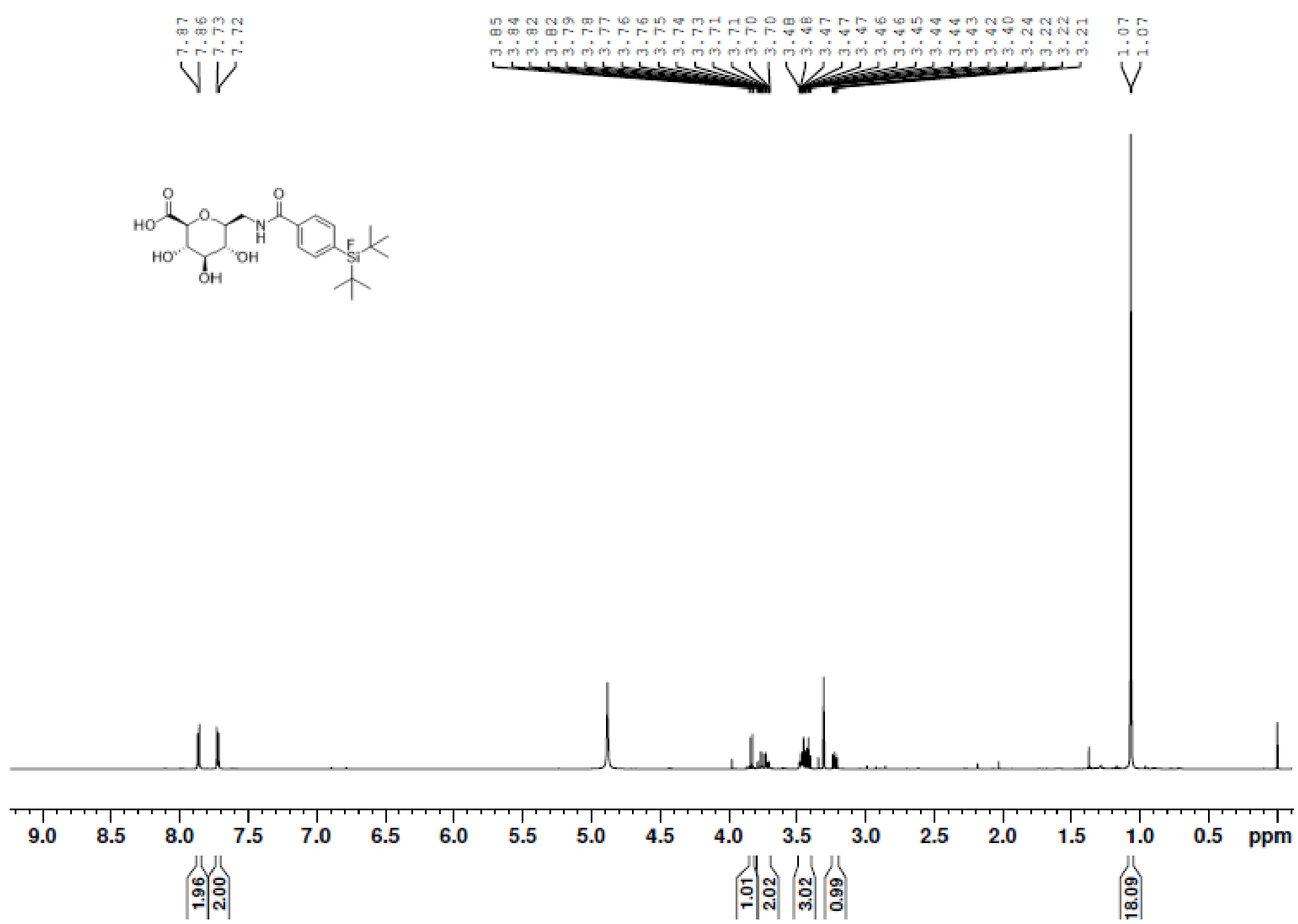

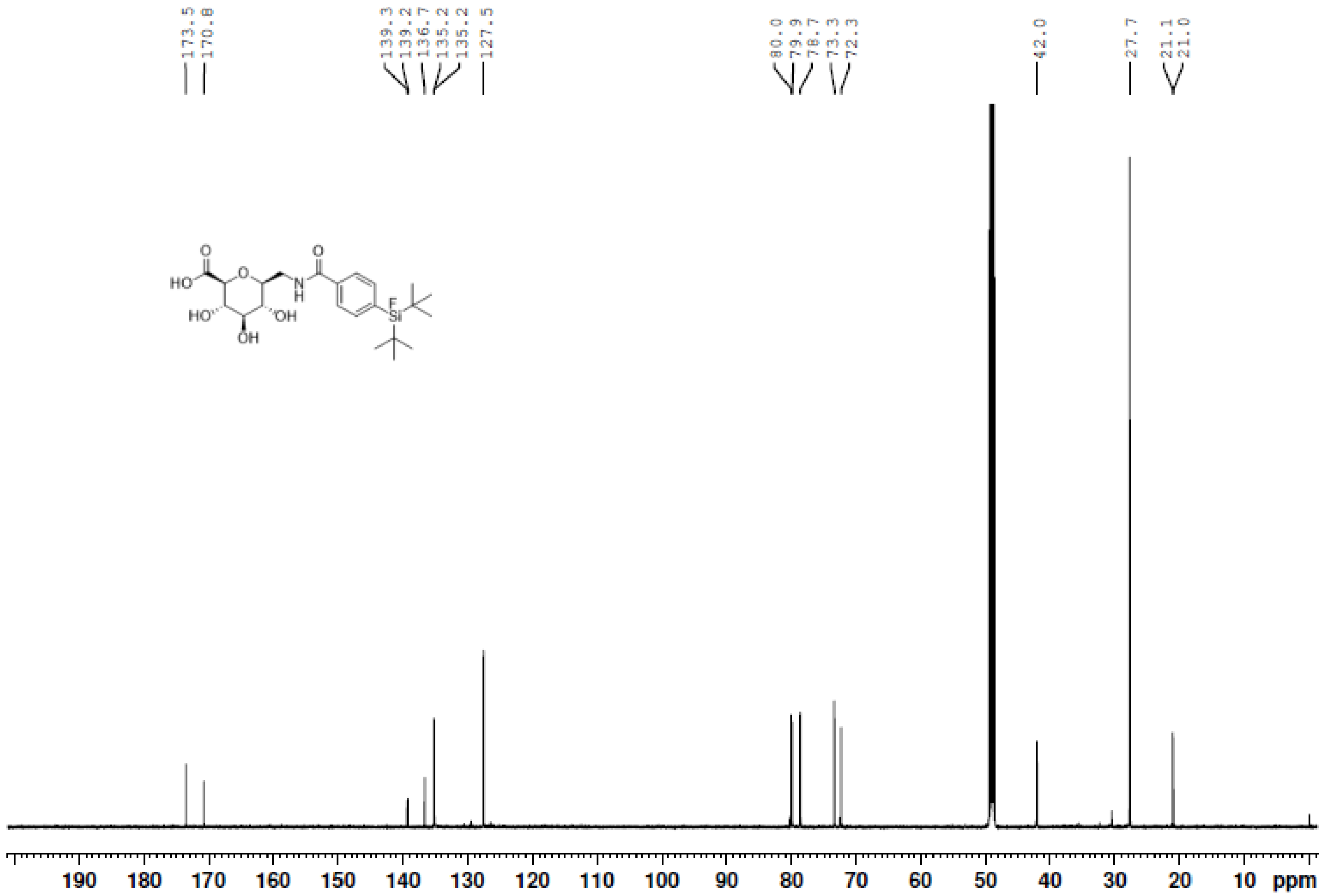

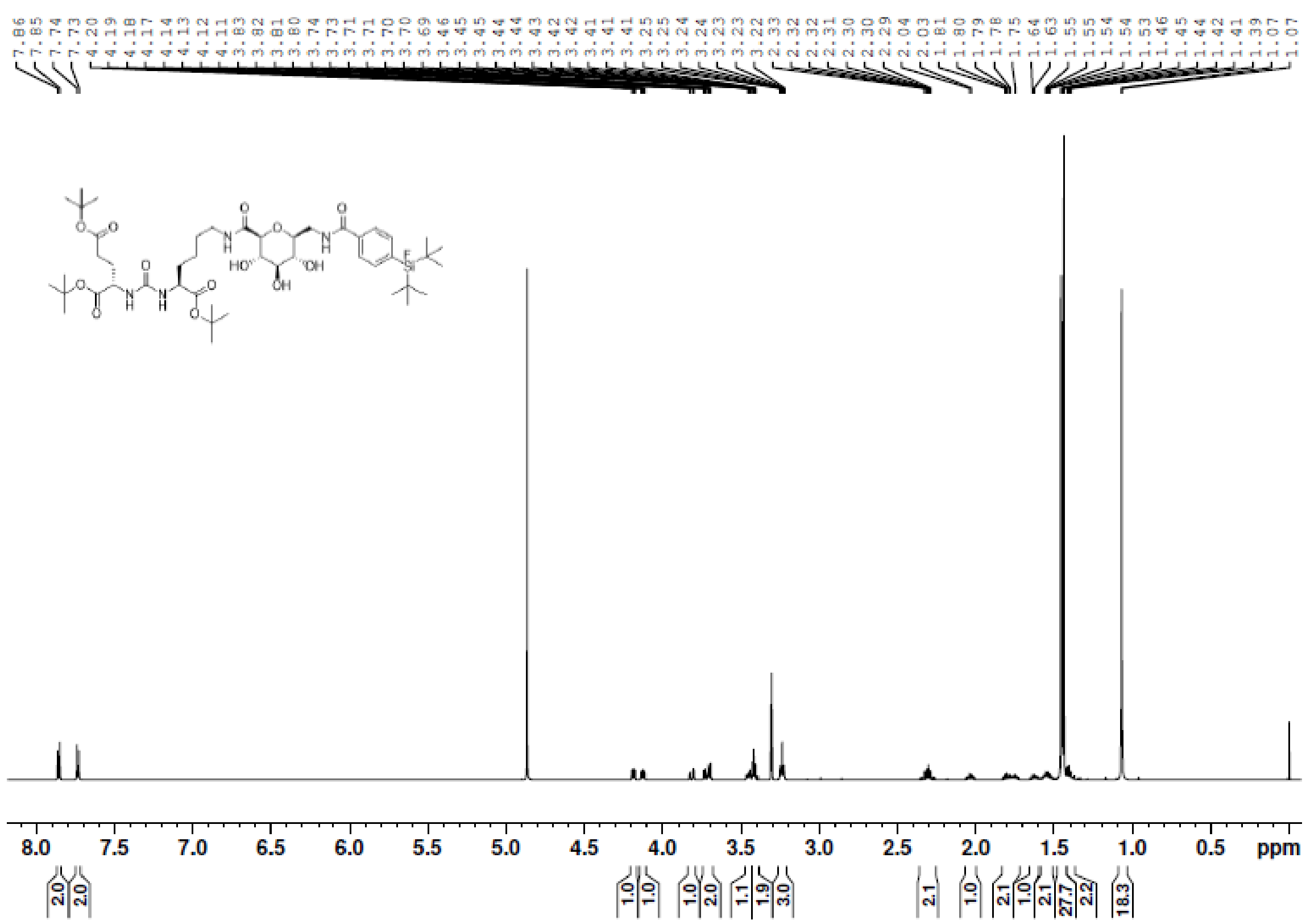

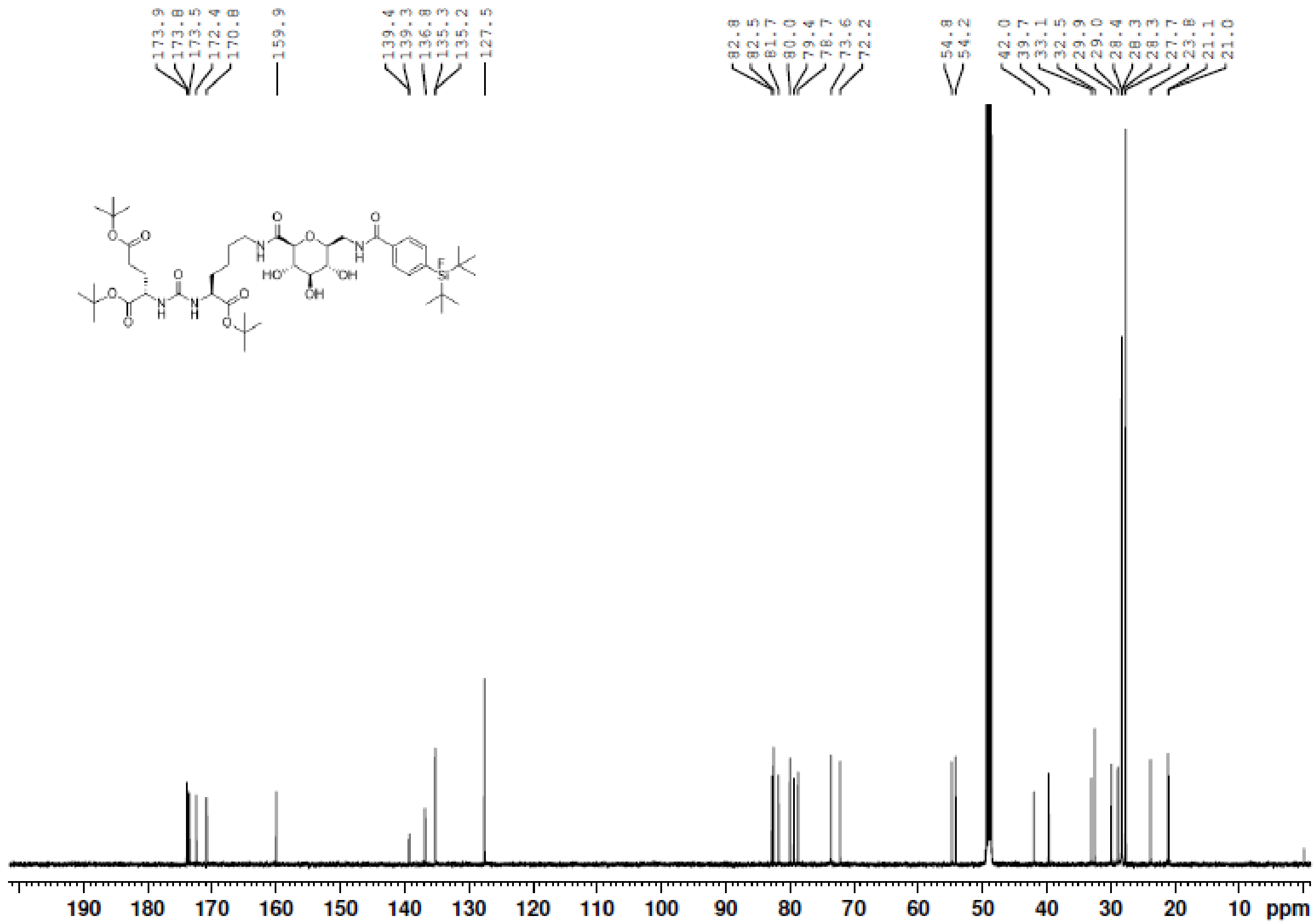
173.9
173.8
173.5
172.4
170.8
159.9
139.4
139.3
136.8
135.3
135.2
127.5
82.8
82.5
81.7
80.0
79.4
78.7
73.6
72.2
54.8
54.2
42.0
39.7
33.1
32.5
29.9
29.0
28.4
28.3
28.3
27.7
23.8
21.1
21.0
190 180 170 160 150 140 130 120 110 100 90 80 70 60 50 40 30 20 10 ppm

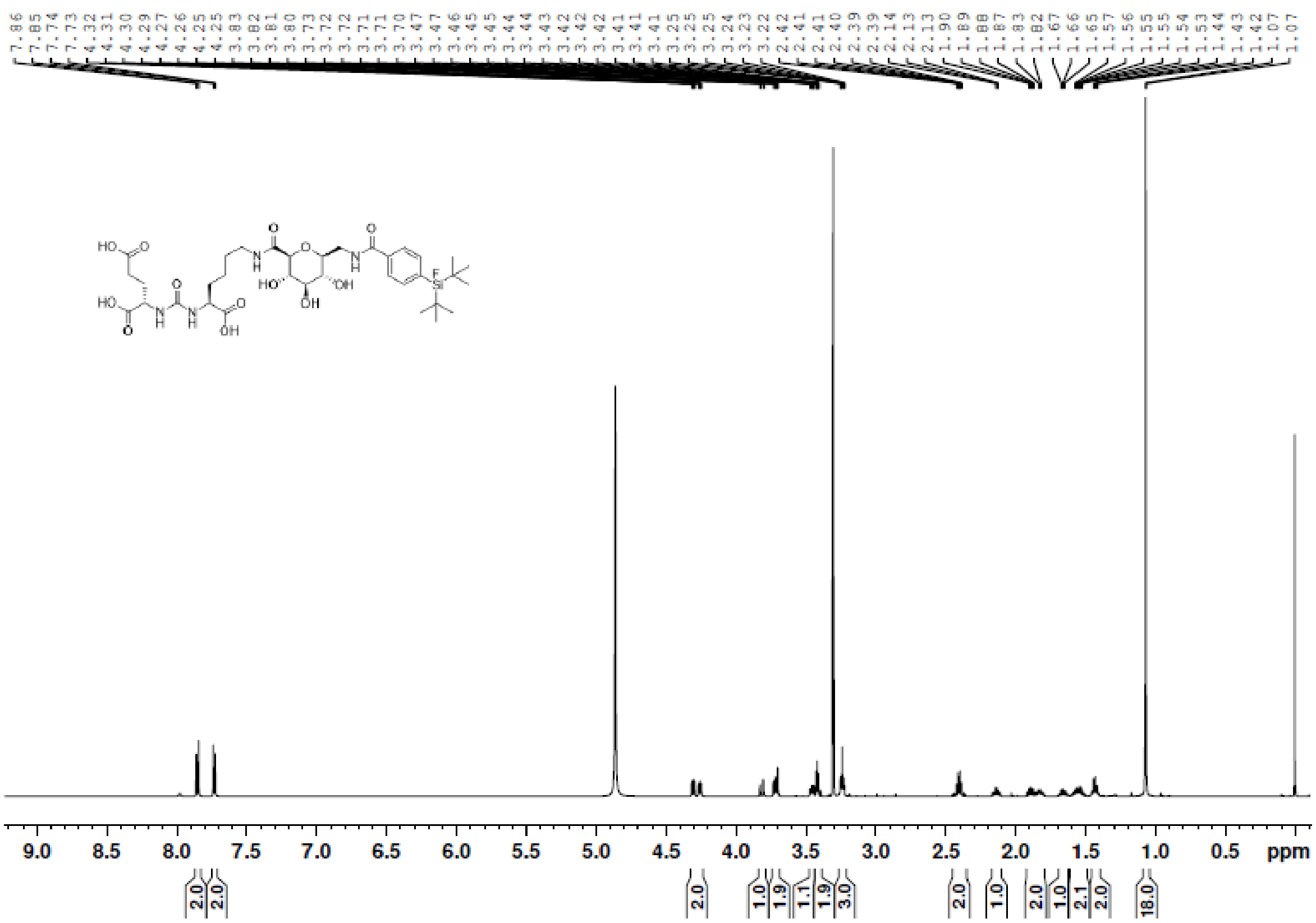
9.0 8.5 8.0 7.5 7.0 6.5 6.0 5.5 5.0 4.5 4.0 3.5 3.0 2.5 2.0 1.5 1.0 0.5 ppm
2.0
2.0
2.0
1.0
1.9
1.1
1.9
3.0
2.0
1.0
2.0
1.0
2.1
2.0
18.0

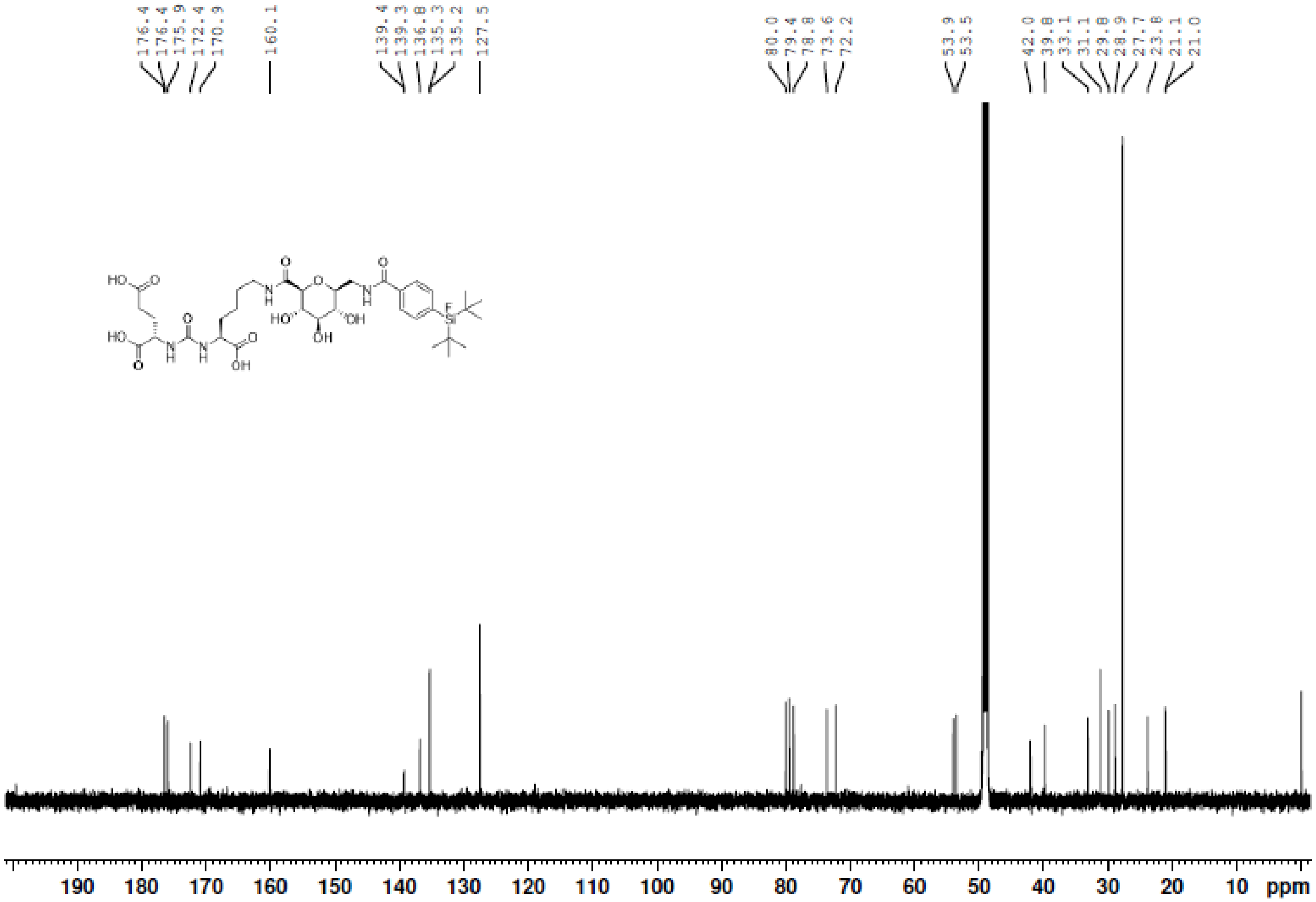
176.4
176.4
175.9
172.4
170.9
160.1
139.4
139.3
136.8
135.3
135.2
127.5
80.0
79.4
78.8
73.6
72.2
53.9
53.5
42.0
39.8
33.1
31.1
29.8
28.9
27.7
23.8
21.1
21.0
190 180 170 160 150 140 130 120 110 100 90 80 70 60 50 40 30 20 10 ppm

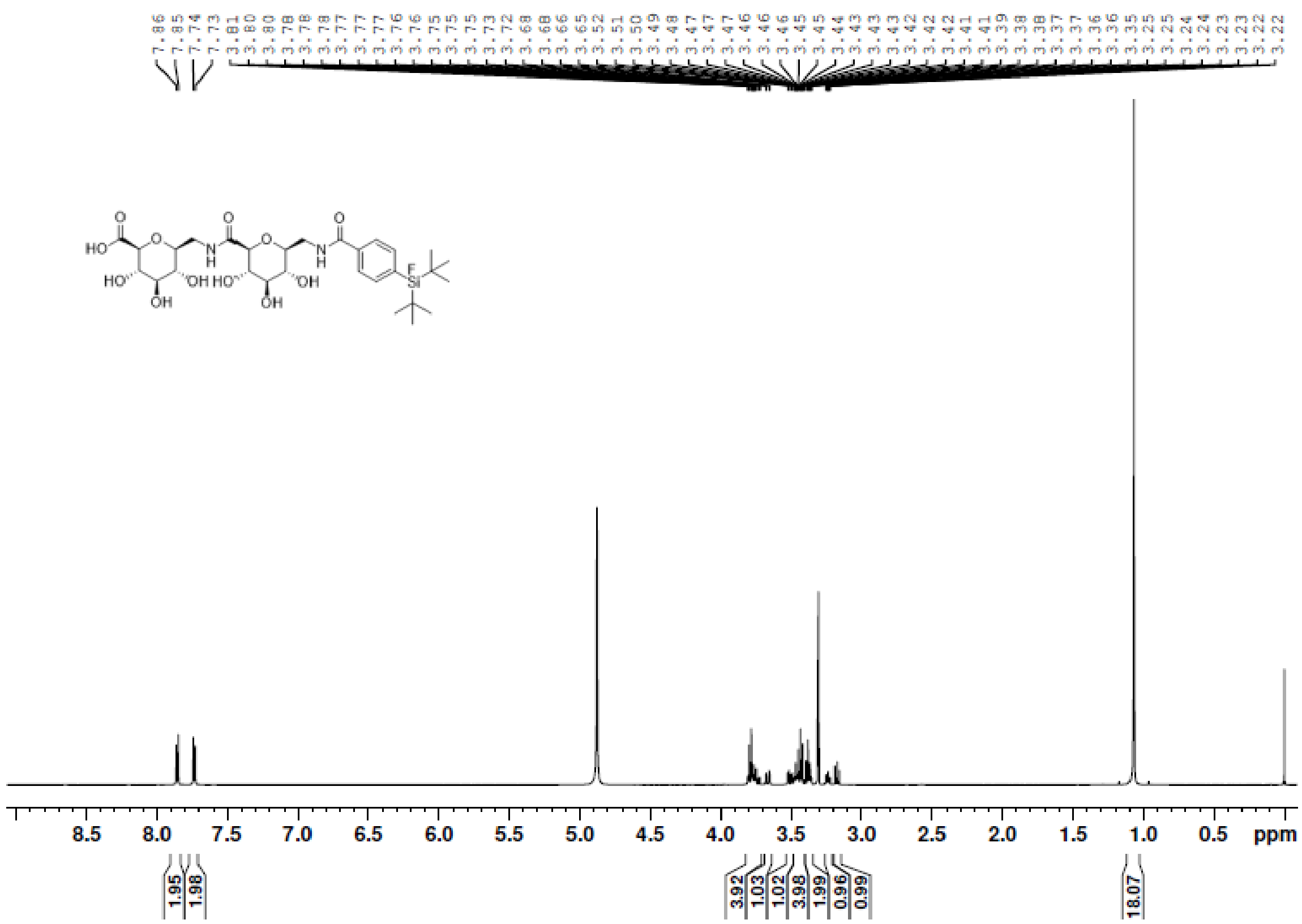
7.86
7.85
7.74
7.73
3.81
3.80
3.80
3.78
3.78
3.77
3.77
3.76
3.75
3.75
3.73
3.72
3.68
3.66
3.65
3.52
3.51
3.50
3.49
3.48
3.47
3.47
3.46
3.46
3.45
3.45
3.44
3.43
3.43
3.43
3.42
3.42
3.41
3.41
3.39
3.38
3.38
3.37
3.37
3.36
3.35
3.35
3.25
3.25
3.24
3.23
3.23
3.22
3.22
8.5 8.0 7.5 7.0 6.5 6.0 5.5 5.0 4.5 4.0 3.5 3.0 2.5 2.0 1.5 1.0 0.5 ppm
1.95
1.98
3.92
1.03
1.02
3.98
1.99
0.96
0.99
18.07

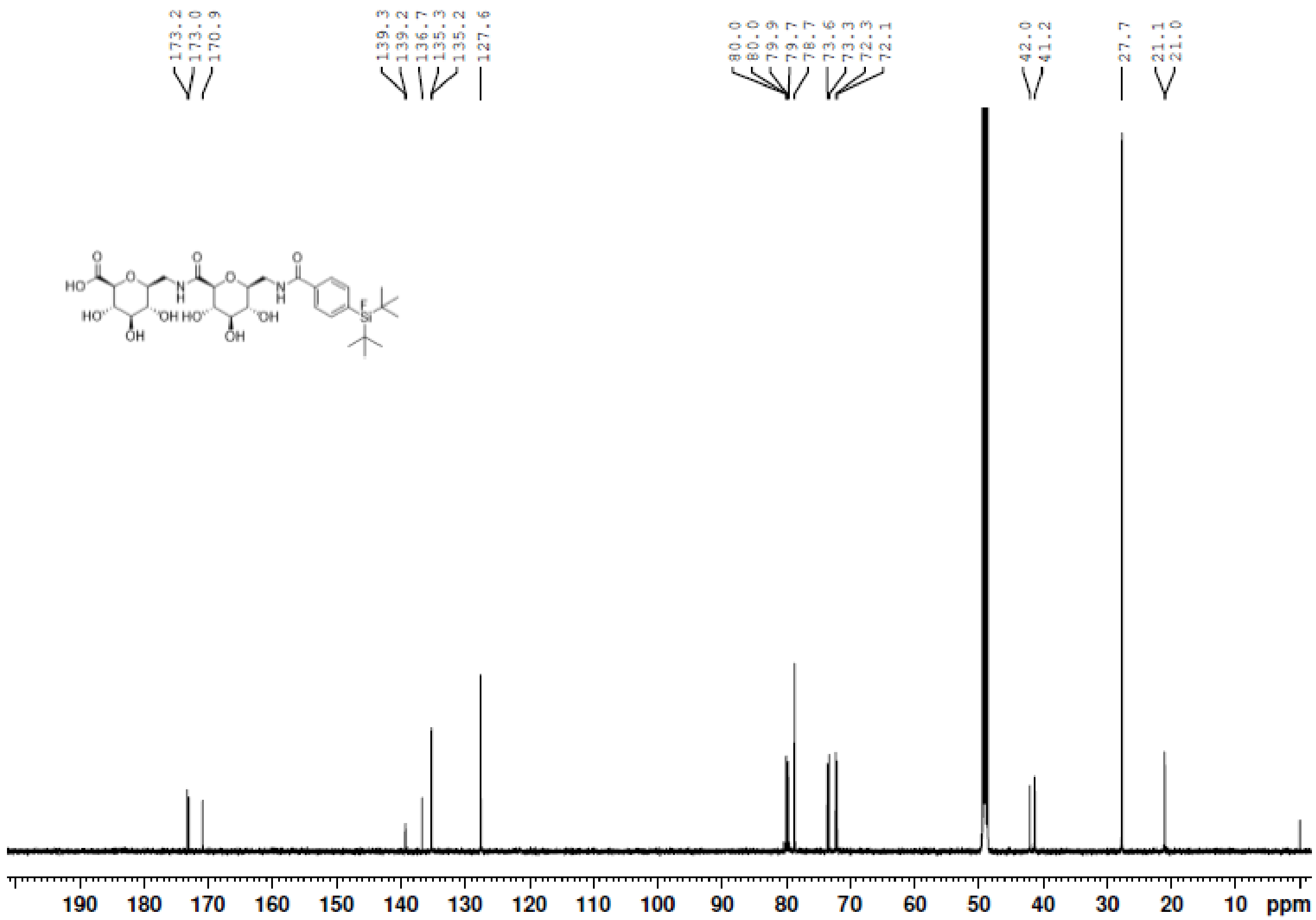
173.2
173.0
170.9
139.3
139.2
136.7
135.3
135.2
127.6
80.0
80.0
79.9
79.7
78.7
73.6
73.3
72.3
72.1
42.0
41.2
27.7
21.1
21.0
190 180 170 160 150 140 130 120 110 100 90 80 70 60 50 40 30 20 10 ppm

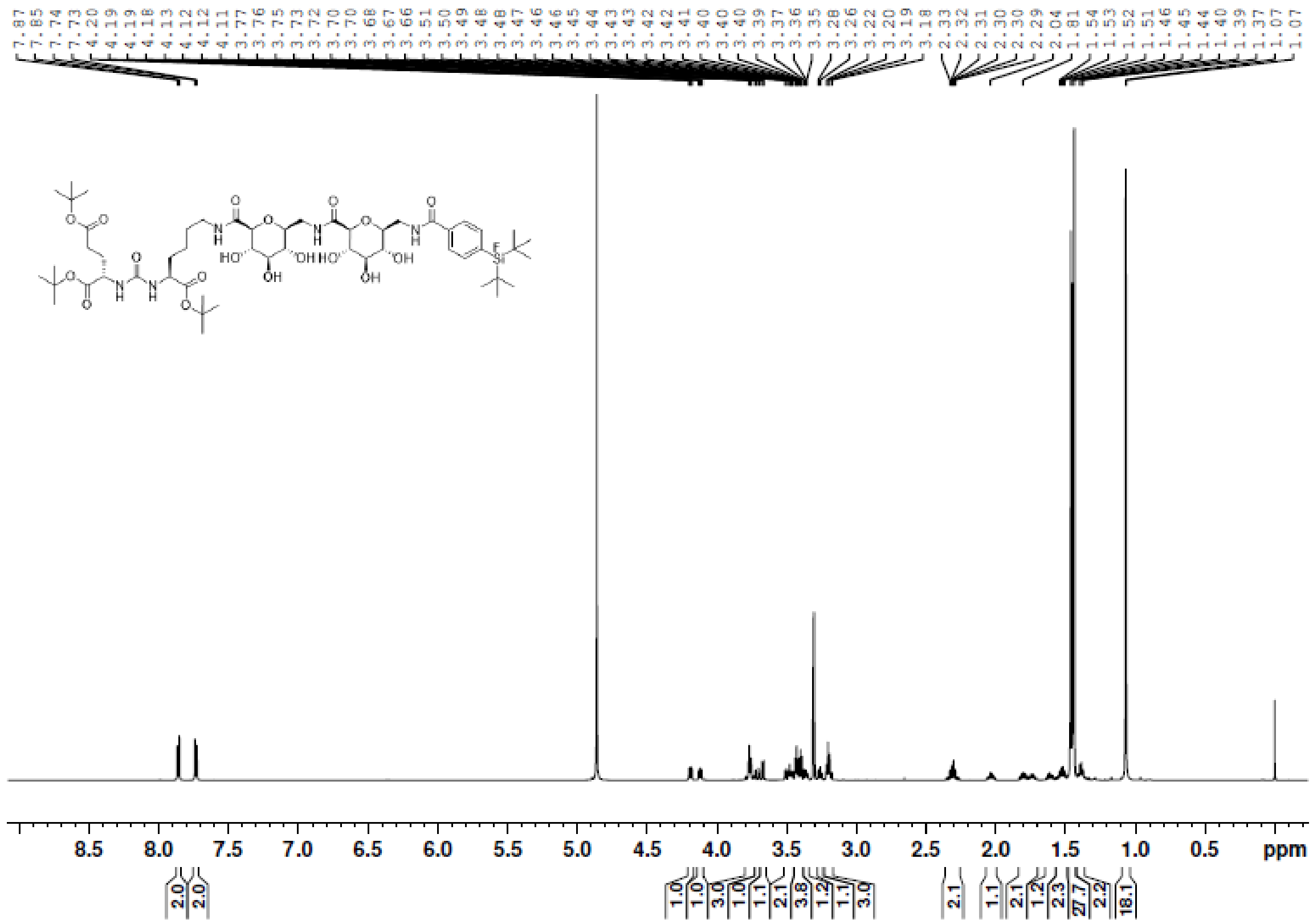
8.5 8.0 7.5 7.0 6.5 6.0 5.5 5.0 4.5 4.0 3.5 3.0 2.5 2.0 1.5 1.0 0.5 ppm

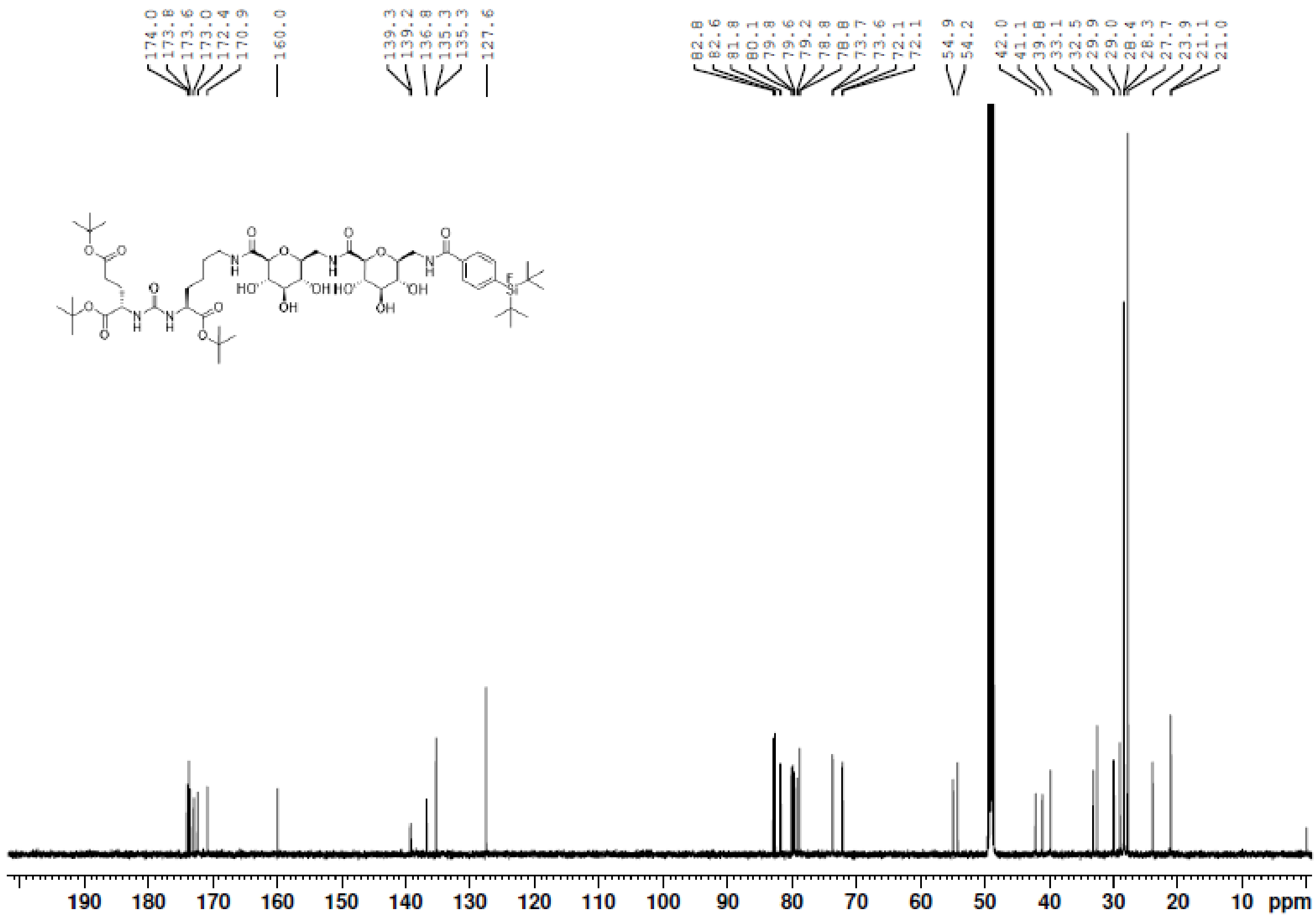
174.0 173.8 173.6 173.0 172.4 170.9 160.0 139.3 139.2 136.8 135.3 135.3 127.6 82.8 82.6 81.8 80.1 79.8 79.6 79.2 78.8 78.8 73.7 73.6 72.1 72.1 54.9 54.2 42.0 41.1 39.8 33.1 32.5 29.9 29.0 28.4 28.3 27.7 23.9 21.1 21.0
190 180 170 160 150 140 130 120 110 100 90 80 70 60 50 40 30 20 10 ppm

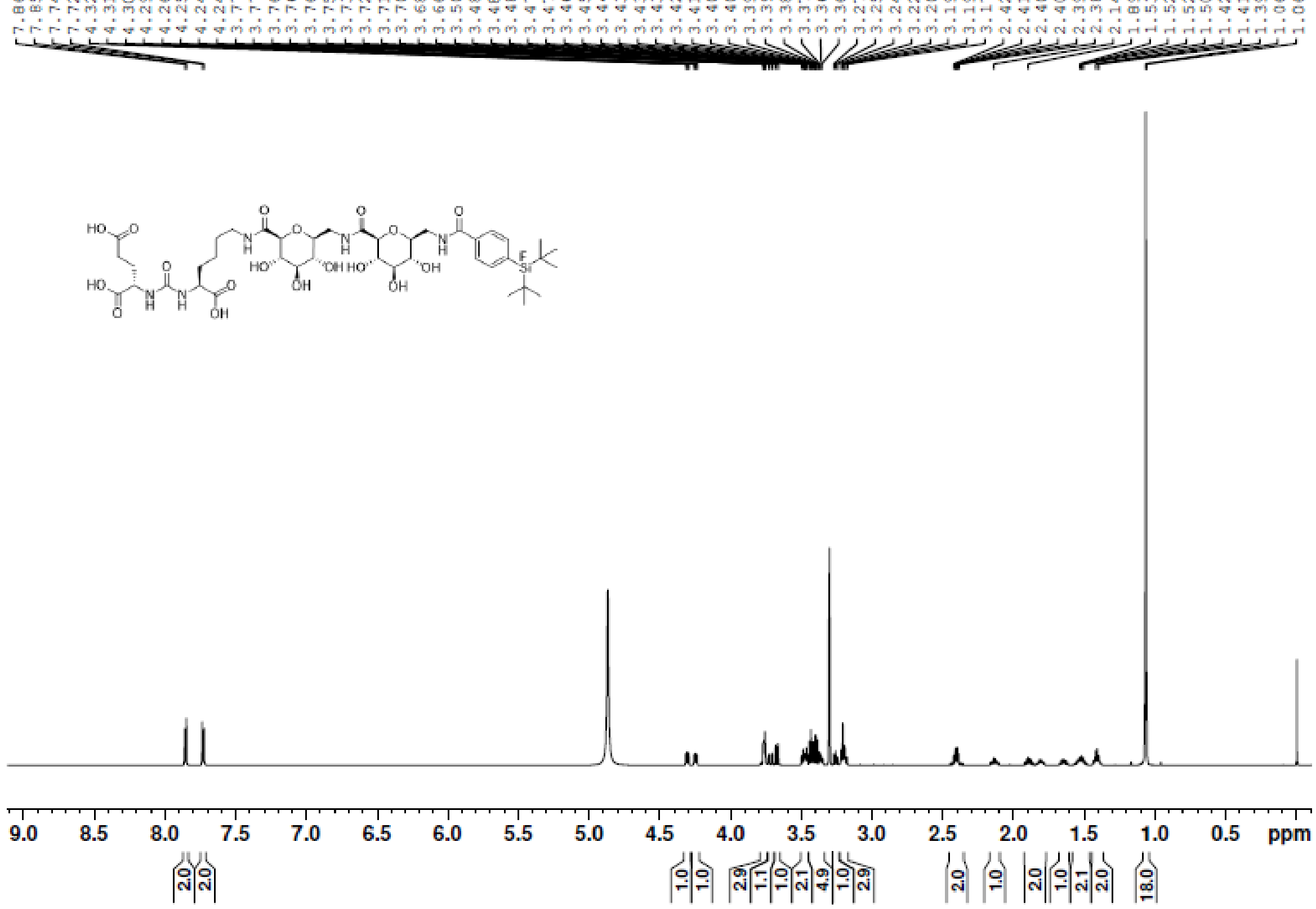
7.86 7.85 7.74 7.72 4.32 4.31 4.30 4.29 4.26 4.25 4.24 4.24 3.77 3.77 3.76 3.76 3.75 3.73 3.72 3.71 3.70 3.68 3.66 3.50 3.48 3.48 3.47 3.46 3.45 3.44 3.43 3.43 3.42 3.41 3.40 3.39 3.38 3.37 3.36 3.27 3.25 3.24 3.22 3.20 3.19 3.17 2.42 2.41 2.40 2.40 2.39 2.38 2.14 1.89 1.53 1.52 1.52 1.50 1.42 1.41 1.39 1.06
9.0 8.5 8.0 7.5 7.0 6.5 6.0 5.5 5.0 4.5 4.0 3.5 3.0 2.5 2.0 1.5 1.0 0.5 ppm
2.0 2.0 1.0 1.0 2.9 1.1 1.0 2.1 4.9 1.0 2.9 2.0 1.0 2.0 1.0 2.1 2.0 18.0

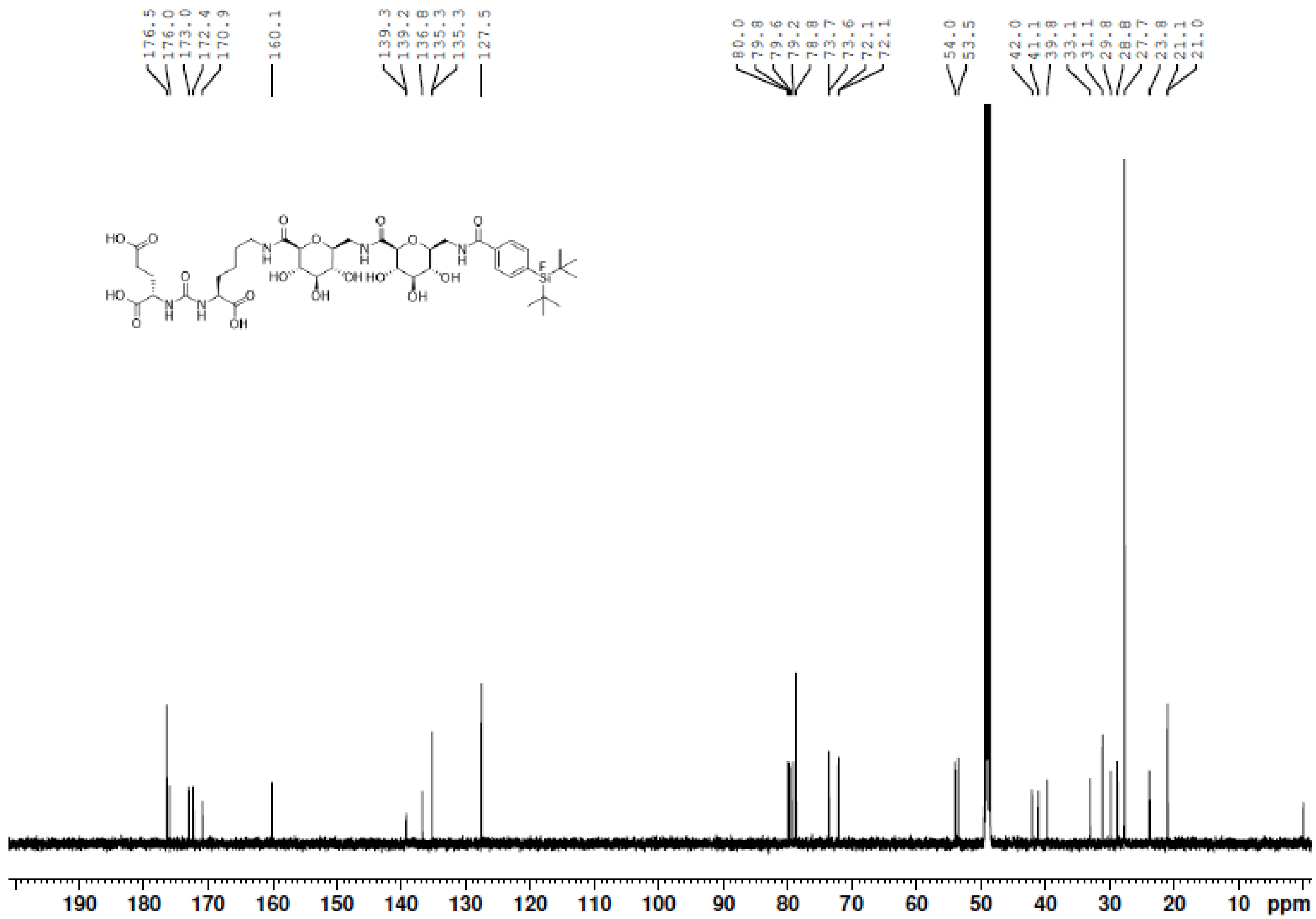
176.5
176.0
173.0
172.4
170.9
160.1
139.3
139.2
136.8
135.3
135.3
127.5
80.0
79.8
79.6
79.2
78.8
73.7
73.6
72.1
72.1
54.0
53.5
42.0
41.1
39.8
33.1
31.1
29.8
28.8
27.7
23.8
21.1
21.0
190 180 170 160 150 140 130 120 110 100 90 80 70 60 50 40 30 20 10 ppm

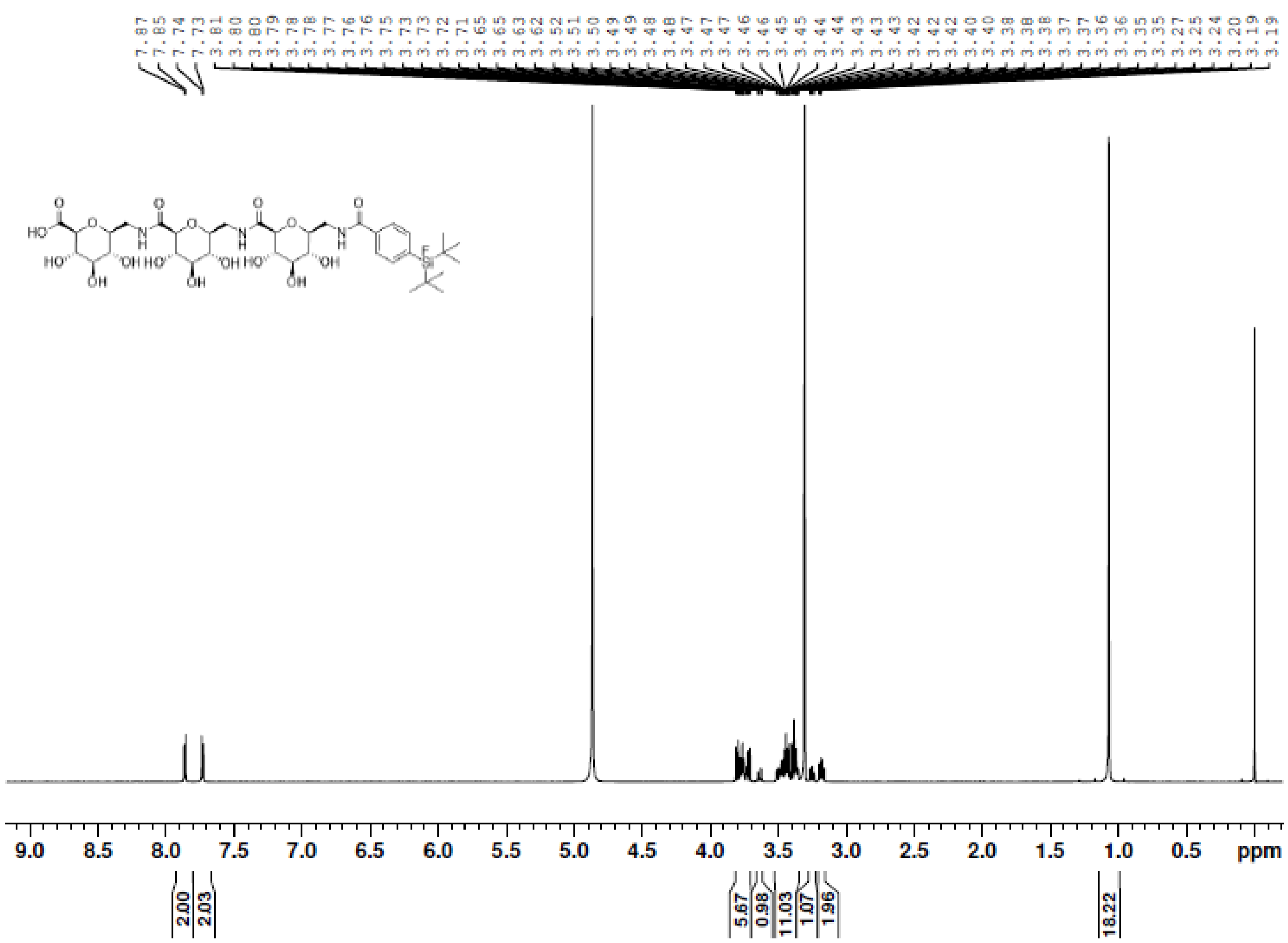
7.87
7.85
7.74
7.73
3.81
3.80
3.80
3.79
3.78
3.78
3.77
3.76
3.76
3.75
3.73
3.73
3.72
3.71
3.65
3.63
3.62
3.52
3.51
3.50
3.49
3.49
3.48
3.48
3.47
3.47
3.46
3.46
3.45
3.45
3.44
3.44
3.43
3.43
3.43
3.42
3.42
3.42
3.40
3.40
3.38
3.38
3.38
3.37
3.36
3.36
3.35
3.35
3.27
3.25
3.24
3.20
3.19
3.19
9.0 8.5 8.0 7.5 7.0 6.5 6.0 5.5 5.0 4.5 4.0 3.5 3.0 2.5 2.0 1.5 1.0 0.5 ppm
2.00
2.03
5.67
0.98
11.03
1.07
1.96
18.22

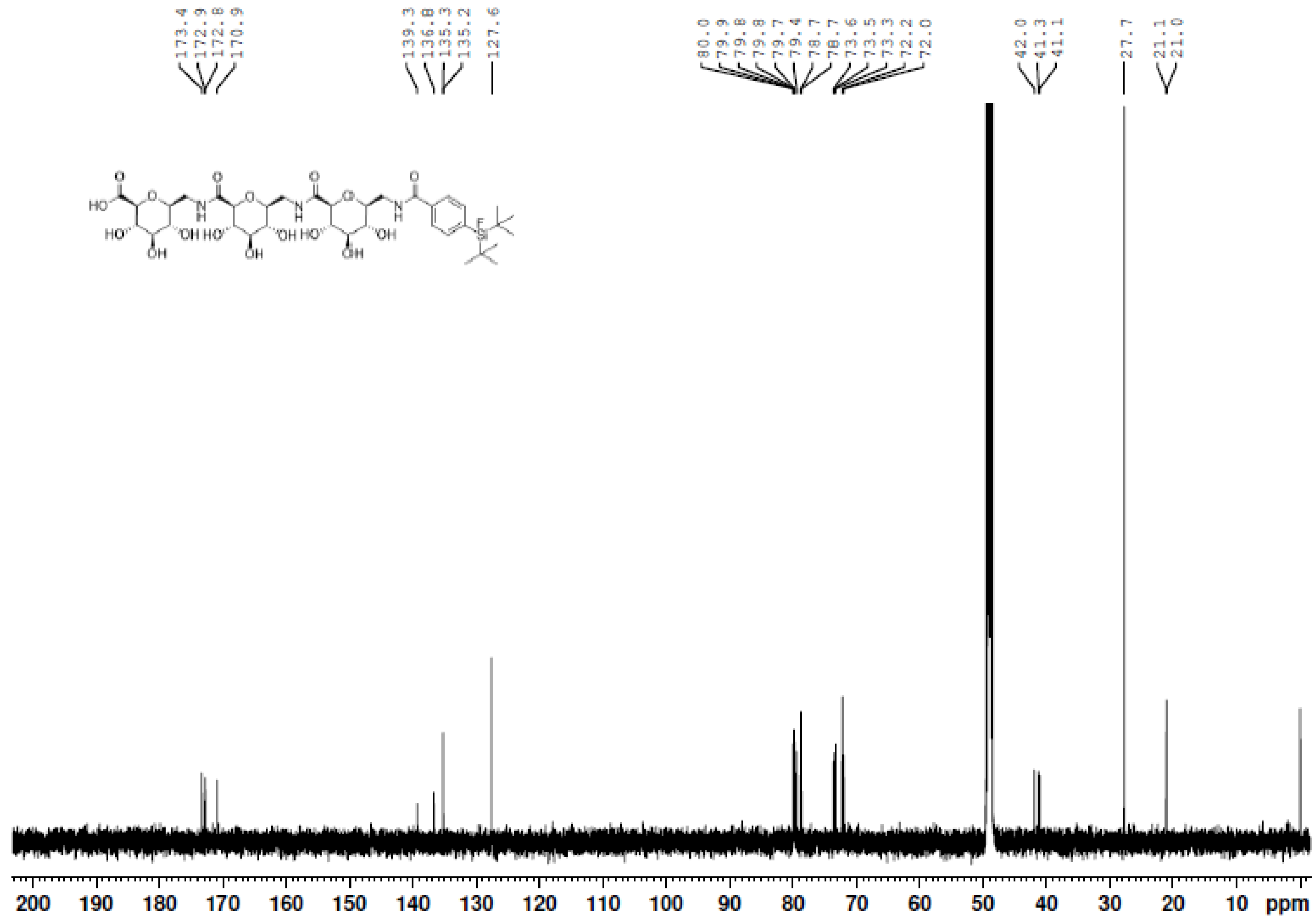

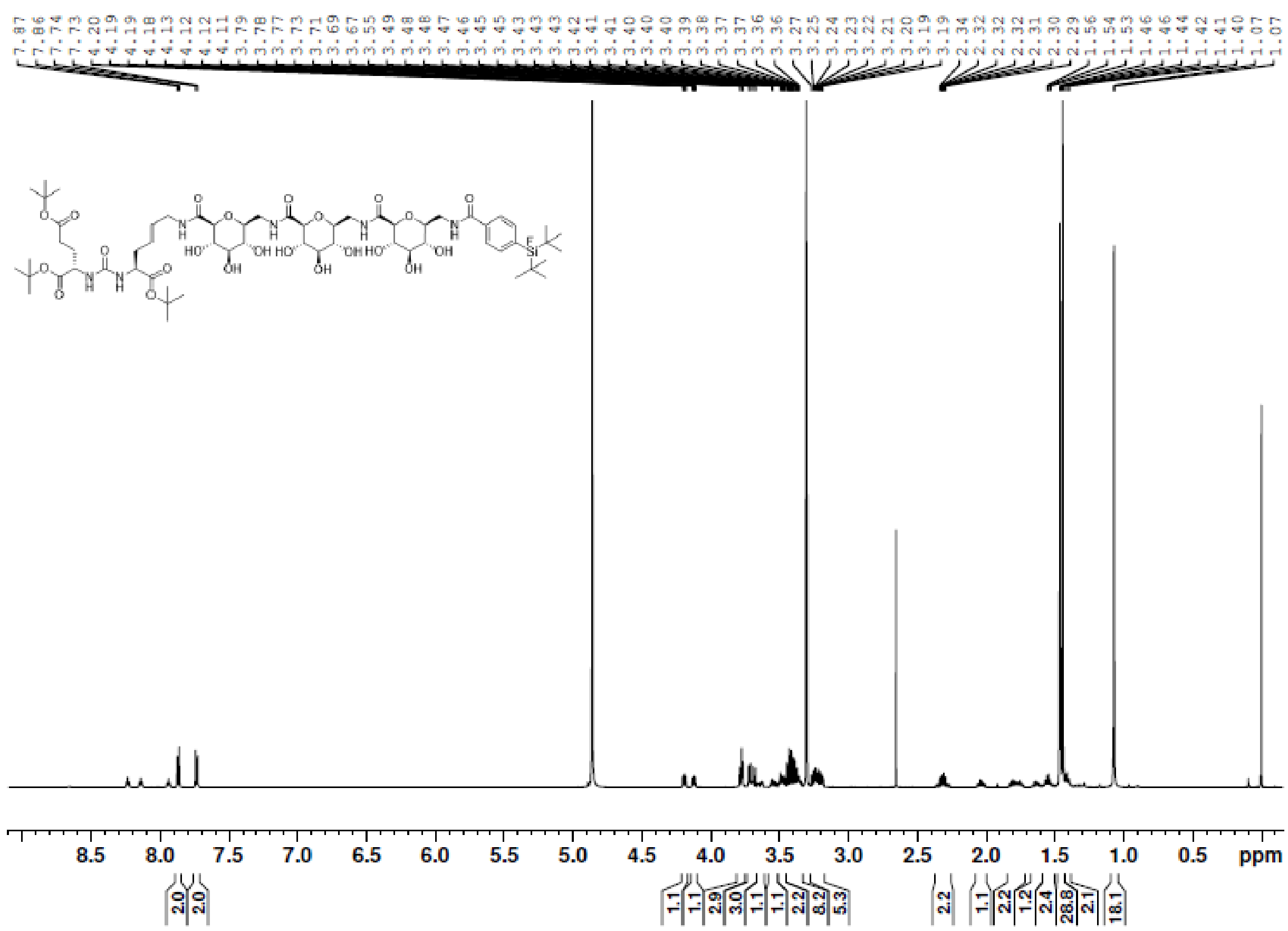

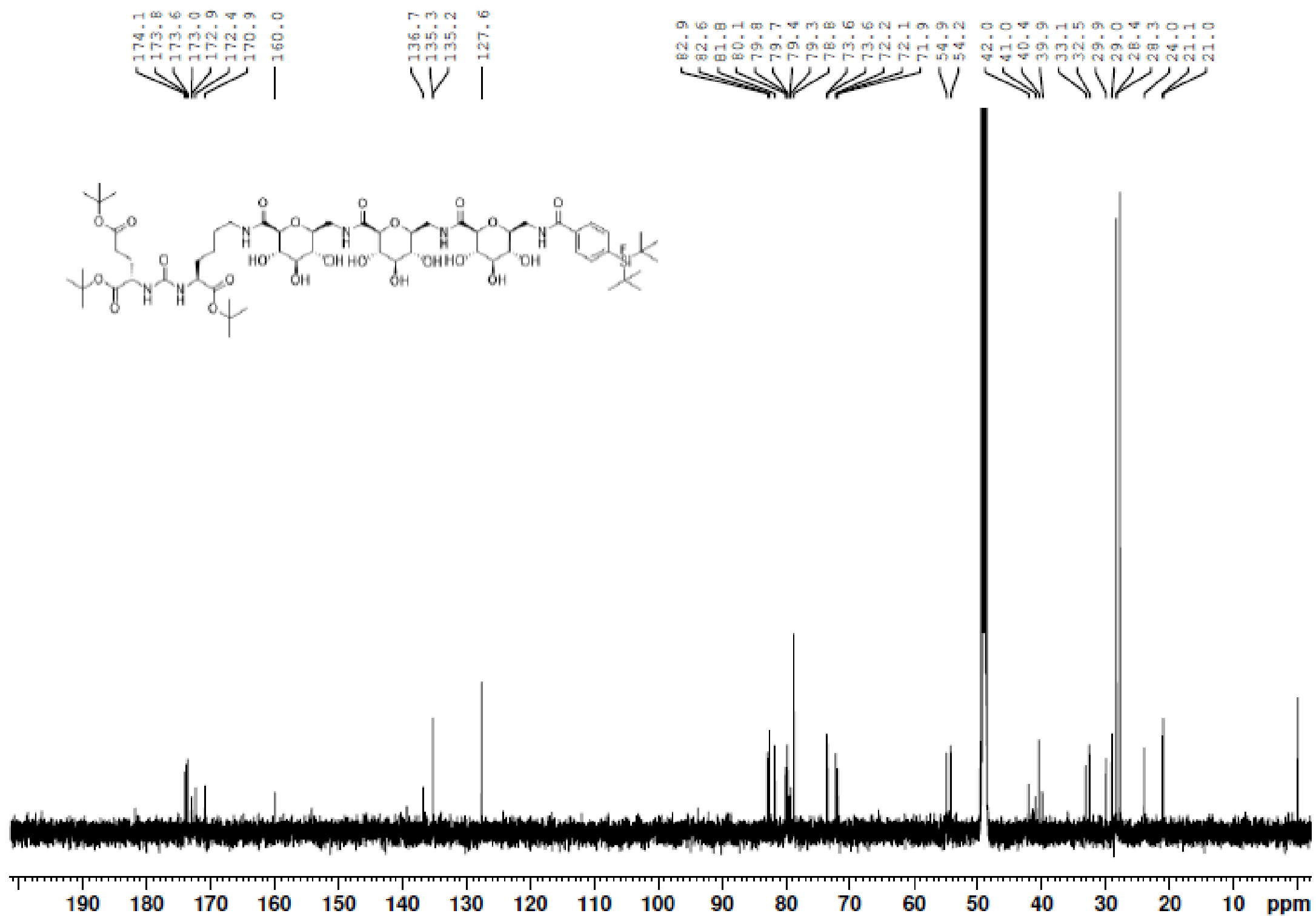

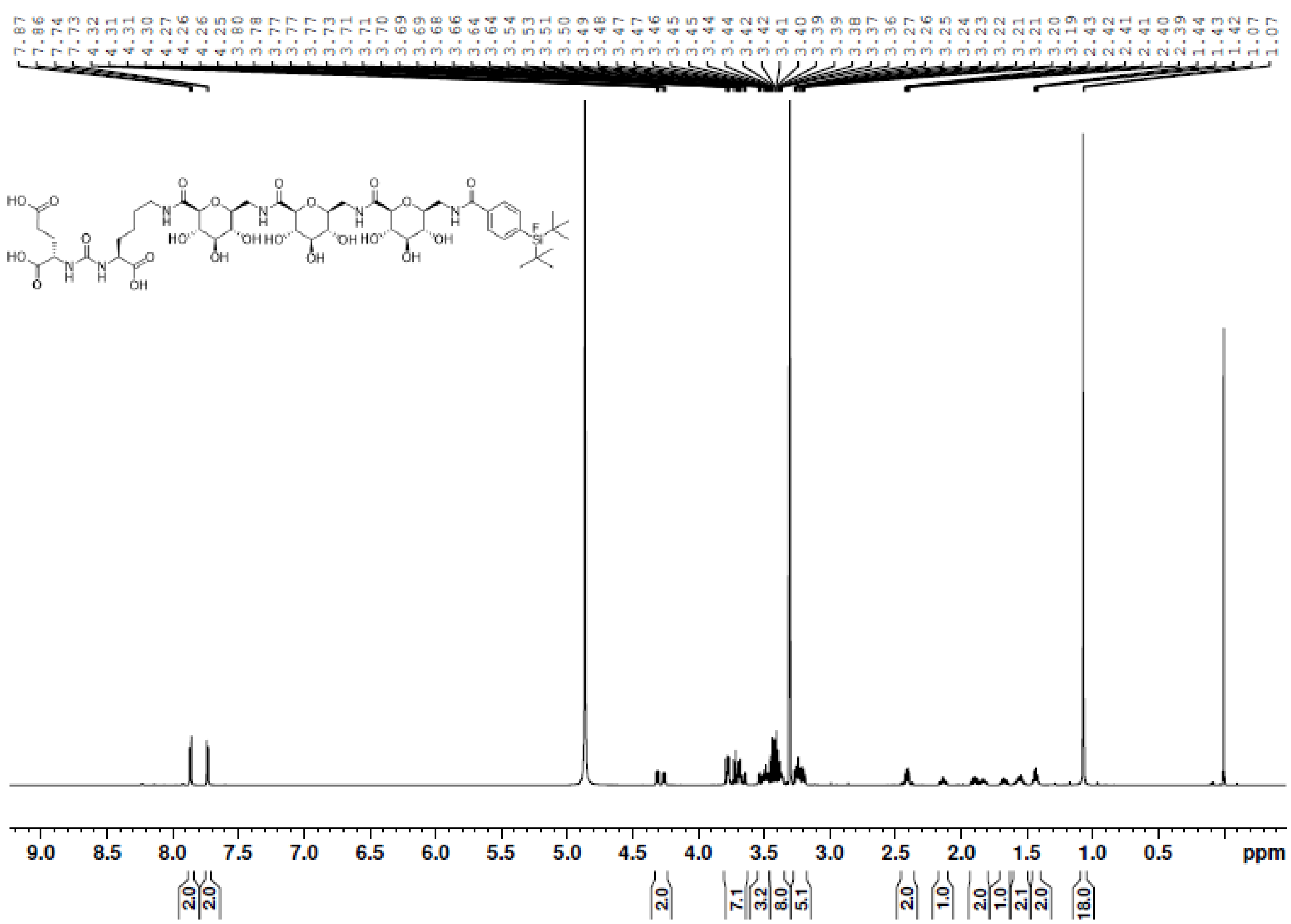

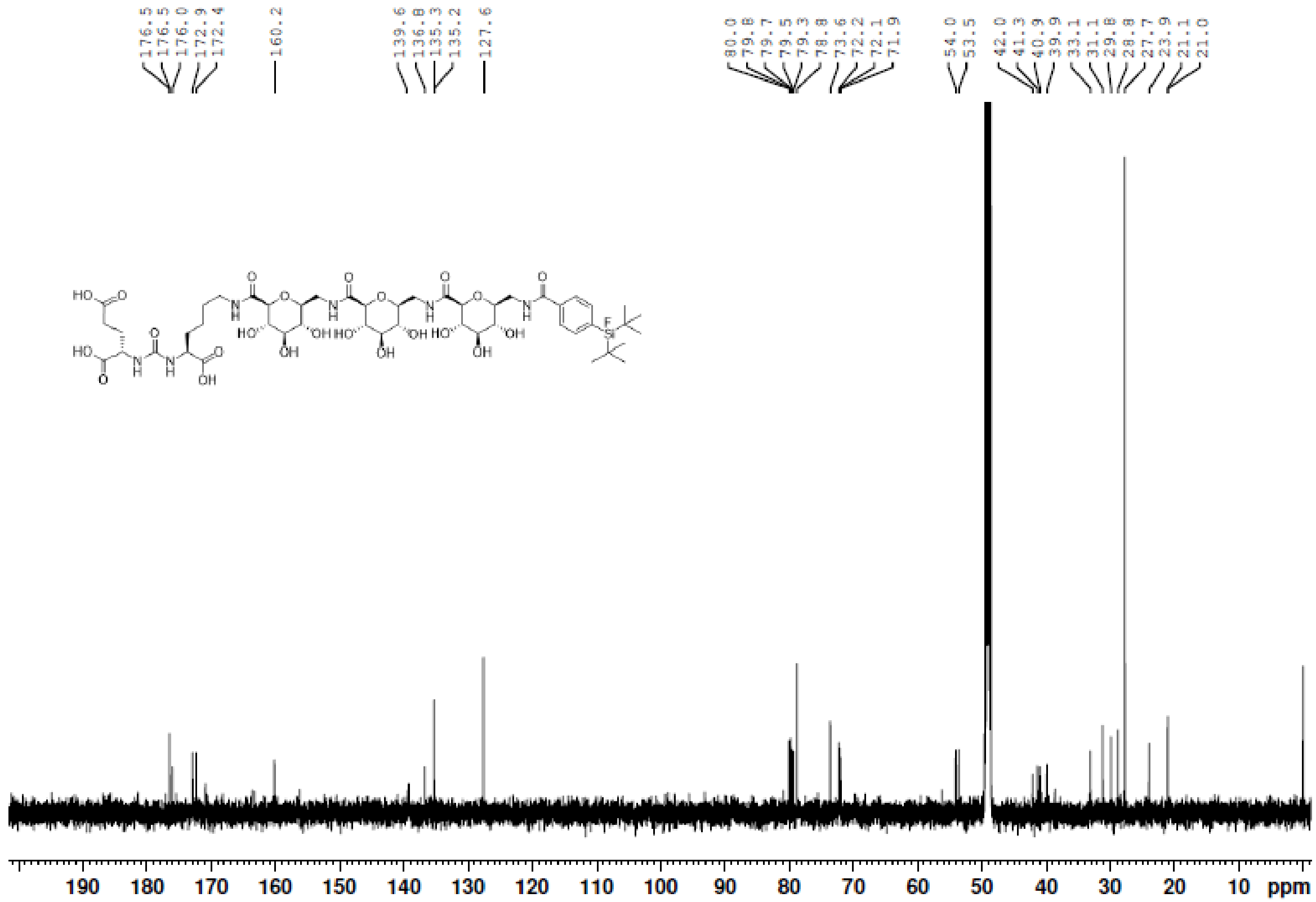

HPLC Quality Control Chromatograms

[^{18}F]19

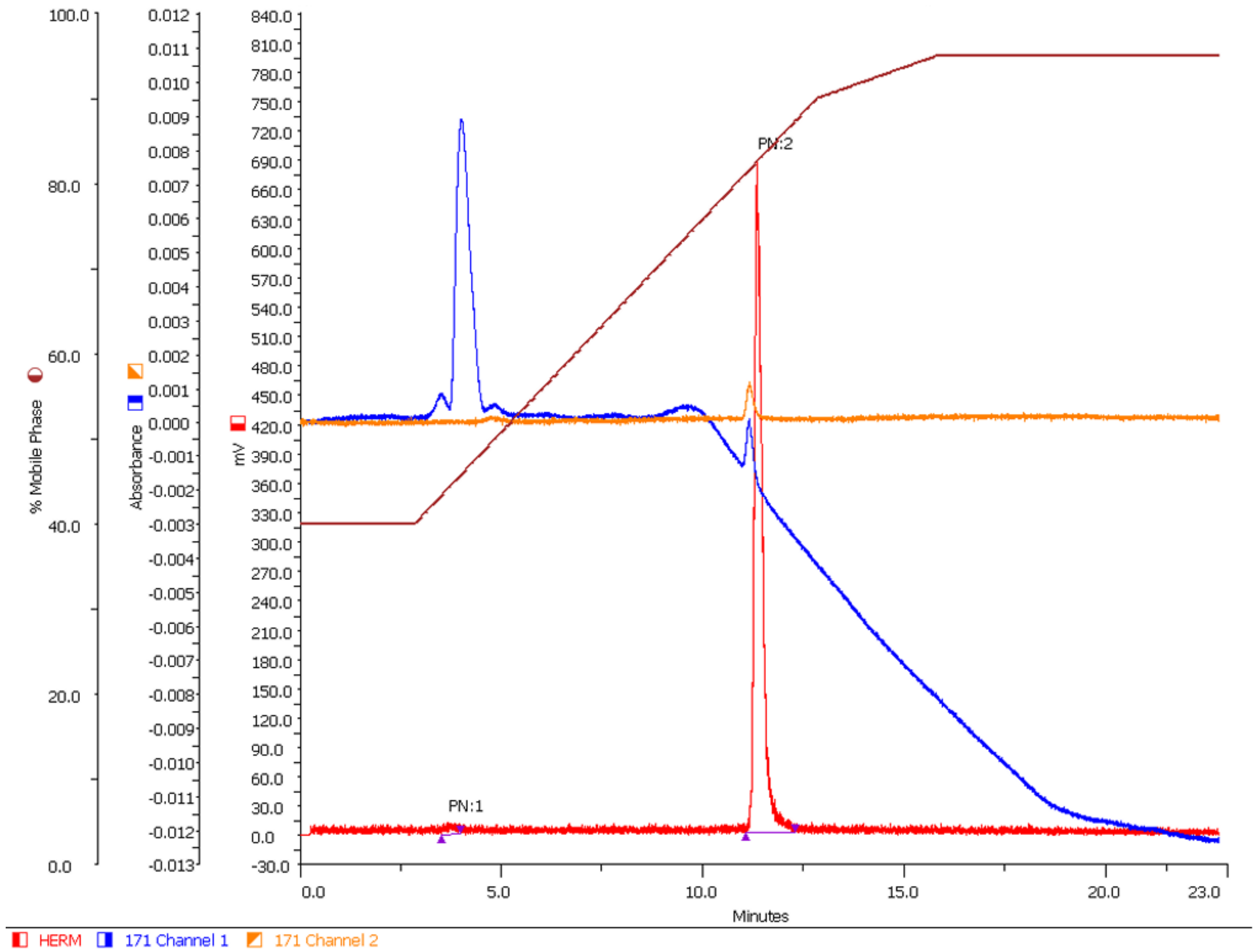

% Mobile Phase
Absorbance
mV
Minutes
PN:1
PN:2
HERM
171 Channel 1
171 Channel 2

[^{18}F]21

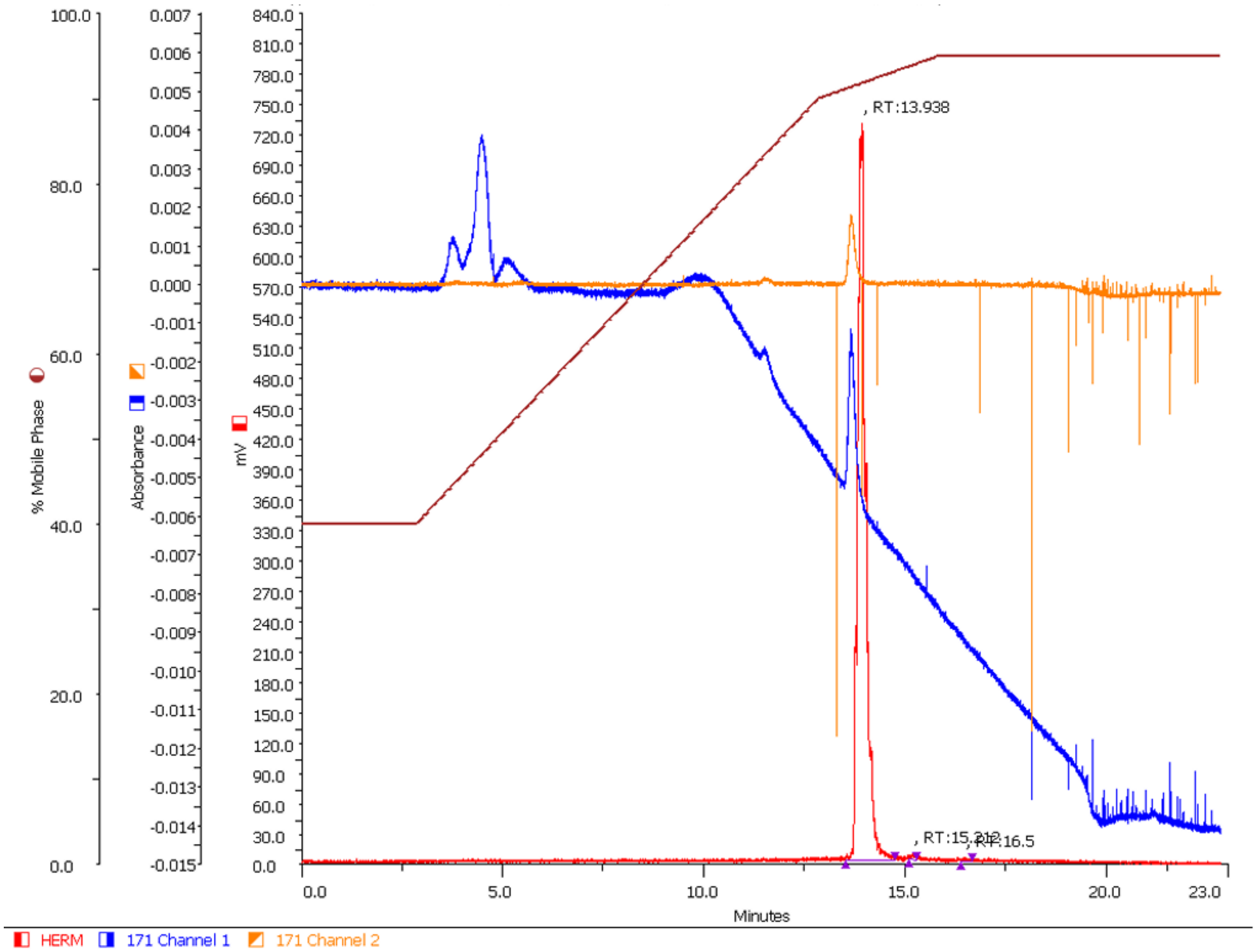

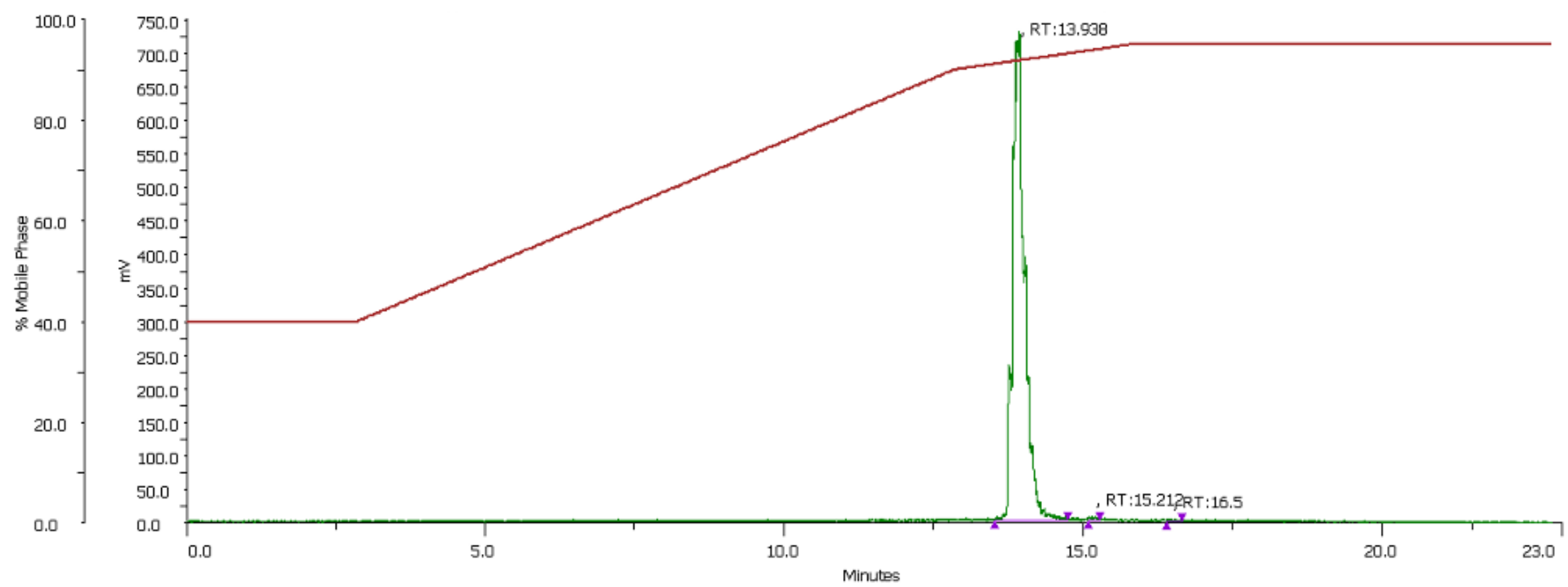

Sample Table

Peak Name	Retention Time (min)	Height (mV)	Area (uVmin x100)	Area %
1	13.938	727.496	17458043.3333	99.425
2	15.212	5.588	56956.25	0.324
3	16.5	4.215	44040	0.251
